# Oxidative Stress in Huntington’s Disease

**DOI:** 10.3390/biom15040527

**Published:** 2025-04-04

**Authors:** Félix Javier Jiménez-Jiménez, Hortensia Alonso-Navarro, Elena García-Martín, Alba Cárcamo-Fonfría, María del Mar Caballero-Muñoz, José A. G. Agúndez

**Affiliations:** 1 Section of Neurology, Hospital Universitario del Sureste, Arganda del Rey, 28500 Madrid, Spain; hortalon@yahoo.es (H.A.-N.); alba.carcamo@salud.madrid.org (A.C.-F.); mcmunoz@salud.madrid.org (M.d.M.C.-M.); 2 University Institute of Molecular Pathology Biomarkers, Universidad de Extremadura, 10071 Cáceres, Spain; elenag@unex.es (E.G.-M.); jagundez@unex.es (J.A.G.A.)

**Keywords:** Huntington’s disease, pathogenesis, oxidative stress, biological markers, animal models

## Abstract

Although the pathogenesis of the neurodegenerative phenomena of Huntington’s disease (HD) is not well known, in the last 30 years, numerous data have been published that suggest a possible role of oxidative stress. The majority of studies regarding this issue were performed in different experimental models of this disease (neurotoxic models such as intraperitoneal injection of 3-nitropropionic acid or intrastriatal injection of quinolinic acid, transgenic animal models for HD, and cell cultures) and, less frequently, in samples of brain tissue, plasma/serum, blood cells, and other tissues from patients with a genetic–molecular diagnosis of presymptomatic and symptomatic HD compared to healthy controls. In this narrative review, we have summarized the data from the main studies in which oxidative stress parameters have been measured both in patients with HD and in experimental models of the same disease, as well as the few studies on gene variants involved in oxidative stress in patients with HD. Most studies addressing this issue in experimental models of HD have shown an increase in markers or oxidative stress, a decrease in antioxidant substances, or both. However, the results of studies on patients with HD have not been conclusive as few studies have been published on the matter. However, a meta-analysis of blood studies on HD patients (including a pool of serum and blood cell studies) has shown an increase in lipid peroxidation markers, OH8dG concentrations, and GPx activity and a decrease in GSH levels. Future prospective and multicenter studies with a long-term follow-up period involving a large number of HD patients and healthy controls are needed to address this topic.

## 1. Introduction

Huntington’s disease (HD), described by George Huntington in 1872, is an infrequent (5–10 cases per 100,000 inhabitants in Western Europe, Venezuela, and the United States, with a lower prevalence in Asian countries) neurodegenerative neuropsychiatric genetic disorder caused by an autosomal dominant mutation consisting of the pathogenic expansion of the trinucleotide repeat cytosine-adenine-guanine (CAG) in the *huntingtin* gene (*HTT* or *IT-15*, chromosome 4p16.3, gene ID 3064, MIM 613004) [1,2,3,4]. From a clinical point of view, HD is characterized by the presence of choreic movements (and, less frequently, other movement disorders, including bradykinesia, rigidity, dystonia, gait disorders, and/or tics), a wide variety of psychiatric symptoms (mainly depression or low mood and, less frequently, anxiety disorders, schizophrenic-like psychosis, emotional lability, or apathy), and progressive cognitive impairment that mainly affects executive attention function [1,2,3,4].

The main histopathological feature of HD is neurodegeneration, which mainly affects the striatum and the cerebral cortex [3]. The previously mentioned pathogenic mutation in the *IT-15* gene leads to the synthesis of abnormal huntingtin (a protein that seems to play an important role in brain development, transcriptional regulation, vesicle transport, and synaptic transmission) [3]. Although the pathogenetic mechanisms of neuronal degeneration are not well understood, it has been suggested that the possible participation of excitotoxicity at cortico-striatal synapses, mitochondrial dysfunction (leading to oxidative stress), GABA dysfunction, abnormal protein fragmentation, neuroinflammation, aberrant immune activation, direct damage/aggregation, reduction in neurotrophic factors (mainly brain-derived neurotrophic factor-BDNF), and altered transcription of essential genes impair astrocyte medium spiny neuron homeostasis [3,5].

The main aim of this narrative review is to summarize the results of studies analyzing the possible role of oxidative stress in the pathogenesis of HD. These studies include a description of oxidative stress marker concentrations in different tissues from patients diagnosed with HD, case–control studies on the possible association of genes related to oxidative stress with the risk for HD, and studies showing the presence of oxidative stress in experimental models of HD. For this purpose, we conducted a literature search using the PubMed Database from 1966 to 27 December 2024, in which we crossed the terms “Huntington’s disease” and “oxidative stress”. The 1378 references retrieved were analyzed manually, one by one, and only those strictly related to the topic were selected.

## 2. Oxidative Stress Markers in Patients with Huntington’s Disease

### 2.1. Oxidative Stress Markers in the Brain

The results and methodology of studies on oxidative stress markers in the brain of patients with HD compared to those of healthy controls (HCs) are described in full detail in Supplementary Table S1 [6,7,8,9,10,11,12,13,14,15,16,17,18,19,20]. Malondialdehyde (MDA) levels were similar in several brain areas from HD and HCs in a study [6], and 4-hydroxynonenal (4-HNE) levels were similar in another [7], while a third study described increased levels of cholesterol oxidation products and reduction in the activities of cholesterol-degrading enzymes in the putamen of HD patients [8]. Protein carbonyl concentrations increased in the striatum and cortex of HD patients in one study [9] but did not differ significantly from those of HCs in another one [6]. Several markers of DNA oxidation were increased in HD [10,11] or similar in HD and HCs in certain brain regions [6].

Glyceraldehyde phosphate dehydrogenase (GADPH) [10,12] and pyruvate kinase activities [12] are similar in the striatum and cerebral cortex of HD patients and HCs, while creatin kinase activity has been found to decrease in the striatum of HD patients [12]. The activities of monoamine oxidases (MAO) B [10,13] and A [13] have been found to increase in basal ganglia structures of HD patients.

Citrate synthase activity is decreased in the putamen [10,12] and increased in the cerebellum of HD patients [10], with contradictory results in the caudate and cortex [10,12]. Mitochondrial complexes II + III decreased in the caudate and putamen [10], complex IV decreased in the putamen [10], and ATP synthase decreased in the striatum and cortex of PD patients [12].

Concerning the mechanisms involved in the defense against oxidative stress, a significant increase in the activity of total superoxide dismutase [9], glutathione peroxidases (GPx) 1 and 6 [9], peroxiredoxins (PRX) 1, 2, and 6 [9], and catalase (CAT) [9] in the striatum and cortex has been described. Also, decreased activity of Cu/Zn-SOD in the parietal cortex and cerebellum decreased oxidized glutathione (GSSG) levels in the caudate [14]; normal Mn-SOD activity in the parietal cortex and cerebellum [10] and normal levels of reduced glutathione (GSH) in the substantia nigra compacta (SNc), caudate, and cerebral cortex [14] have been shown in HD patients.

Iron concentrations are increased in the pallidus [15,16,17,18] and have been reported to be normal [15,18] or increased in the striatum of patients with HD [16,17]. Total ferritin [19] and light chain of ferritin levels were increased [9], and aconitase activity was decreased in the striatum of HD patients [9]. Loeffler et al. [20] described decreased copper levels in the SNc, the hippocampus, and the parietal cortex and increased ceruloplasmin in the SNc, hippocampus, and parietal cortex, while Scholefield et al. [15] reported decreased copper concentrations in the cerebellum. This group described changes in the concentrations of certain metals in diverse brain regions, with a widespread reduction in selenium levels [15], while another group described decreased selenium concentrations only in the SNc [21].

Finally, in comparison to the HCs, pyridoxal kinase (PDXK) activity was decreased in the striatum and cortex [22], glycogen synthase kinase-3β (GSK-3β) activity increased in the hippocampus [23], glycerophosphocholine phosphodiesterase 1 (GPCPD1) expression was decreased in the cortex and striatum [24], and uric acid concentrations were decreased in the prefrontal cortex of HD patients [25].

### 2.2. Oxidative Stress Markers in Plasma/Serum

Supplementary Table S2 summarizes the results and the methodology of studies addressing plasma [26,27,28,29,30,31,32,33,34,35,36,37,38] or serum [39,40] concentrations of diverse oxidative stress markers in patients diagnosed with HD and HCs. Regarding lipid peroxidation markers, plasma/serum concentrations of MDA/TBA/TBARS [26,27,28,29], 4-HNE [26,29], and lipid peroxides [31,32] are increased in HD patients compared to HCs, except for a study by Olsson et al. [30], which described similar plasma MDA levels in HD patients and HCs.

Plasma levels of protein carbonyls increased in HD patients [31,33] and in asymptomatic *HD* gene carriers [31] in two studies and were similar to those of HCs in another [30]. Similarly, plasma levels of advanced oxidation protein products (AOPPs) have been reported to be increased in HD patients [33]. Plasma or serum levels of the main oxidation product of DNA, i.e., 8-hydroxy-deoxyguanosine (OH^8^dG), have been reported to be significantly higher [33,39] or similar [34] to those of HCs, although a longitudinal study with presymptomatic HD patients showed the lowest levels for HCs and the highest levels for subjects at higher risk of developing HD [35]. Plasma global oxidant status was increased, and total antioxidant capacity was decreased in HD patients in a study [33], while another described similar total antioxidant capacity in HD and HCs [29]. Plasma protein thiol concentrations were similar to those of HCs in a study [29].

Plasma levels of superoxide anion [31] and total glutathione [33] and plasma activity of total SOD [29,31] and catalase [29] have been described to be similar in HD and HCs, although one study described a higher Cu/Zn-SOD activity in HD patients [39]. Plasma GPx activity increased in HD patients in one study [29] and was similar between HD patients and HCs in another [31]. Plasma oxidized glutathione levels (GSSG) were increased, and glutathione reductase (GR) activity was decreased in HD patients [33]. Plasma levels of reduced glutathione (GSH) decreased in HD patients [31,33] and in asymptomatic *HD* gene carriers [31] or were similar between HD patients and HCs [29].

Some isolated studies described increased plasma myeloperoxidase (MPO) [36] and aminopeptidase activities [32], increased serum neuron-specific enolase (NSE) [39], increased plasma lactate levels [32], decreased plasma thioredoxin [36] and serum carnitine levels [40], and decreased plasma thioredoxin reductase 1 activity [36] in patients with HD. Mean and acrophase plasma melatonin levels decreased in HD [37], and plasma uric acid levels decreased in females with premanifest HD and symptomatic HD compared to female HCs [25]. Plasma oxyhemoglobin and alpha-1-microglobulin have been found to be similar in HD patients and HCs [30].

Finally, a study measuring plasma levels of trace metals showed increased iron, zinc, selenium, chromium, and arsenic, decreased lead, vanadium, and antimonium, and normal levels of copper and manganese in comparison to HCs [38]. In another study, plasma selenium concentrations did not differ from those of HCs [21].

### 2.3. Oxidative Stress Markers in Blood Cells and Fibroblasts

The results and methodology of studies addressing markers of oxidative stress in leukocytes, peripheral blood mononuclear cells, erythrocytes, and skin fibroblasts of HD patients are described in full detail in Supplementary Table S3 [28,33,36,41,42,43,44,45,46].

A study performed in *leukocytes* found increased levels of the DNA oxidation product OH^8^dG and of deleted and total mitochondrial DNA (mtDNA) copy numbers in patients with HD, while mRNA expression levels of mtDNA-encoded mitochondrial enzymes, and expression levels of NADH dehydrogenase subunit 1 (ND1), cytochrome b (CYTB), and cytochrome c oxidase I (COXI) were similar to those of HC patients [28]. In contrast, another study described decreased total mtDNA copy numbers in patients with HD [41]. PerezGrovas-Saltijeral et al. [47], in a study involving 71 HD patients, 29 asymptomatic carriers of the *HD* gene, and 102 HCs, showed that HD patients have a shorter relative telomeric length in the leukocytes than the other two groups (which were telomere shortening related to DNA damage caused by reactive oxygen species and defective DNA repair mechanism).

A study *on peripheral blood mononuclear cells* showed decreased activity of aconitase-2 (an enzyme involved in the tricarboxylic acid cycle and in iron metabolism) in patients with HD [32].

Studies on *erythrocytes* have shown increased lipid peroxides [33] and protein carbonyl concentrations [33] and GR [43], hexokinase [43], and pyruvate kinase activities [43], decreased GSH [33] and thioredoxin-1 (Trx-1) concentrations [36], decreased Cu/Zn-SOD [28], GPx [28], CAT [43], and thioredoxin reductase 1 (TrRD-1) activities [36], and non-significant differences between HD and HC patients of GSSG [33] and LDH concentrations [43], as well as GADPH [43] and ATPase activities [43]. Total glutathione concentrations have been found to be decreased [43] or similar to those of HCs [33] in two different studies.

Two studies on *skin fibroblasts* have shown non-significant differences between HD patients and HCs regarding Cu/Zn-SoD, GPx, and mitochondrial respiratory chain complexes I–V [44,45]. GR activity increased [44], ATP levels decreased [44], and cytosolic ROS [44], mtO2•− [44], and CoQ_10_ levels [45], as well as mitochondrial membrane potential [44], were similar to those of HCs. Mn-SOD activity increased in HD patients in one study [44] and was similar to that of HCs in another [45]; CAT activity decreased in one study [45] and was similar to that of HCs in another [44]. Finally, Ooi et al. [46] found increased MAO-A mRNA and MAO-A activity in the fibroblasts of HD patients.

A meta-analysis of studies on blood oxidative markers (combining results of plasma, serum, and erythrocytes), involving 375 HD patients and 447 HCs from 12 studies, showed an increase in lipid peroxidation markers and OH^8^dG concentrations and in GPx activity, a decrease in GSH levels, and similar SOD activity in HD patients compared to HCs [48].

### 2.4. Oxidative Stress Markers in Other Body Fluids

Milstien et al. [49] reported a lack of differences between HD patients and HCs in nitrate + nitrite and quinolinic acid levels in the *cerebrospinal fluid.* Olsson et al. [30] found similar MDA and protein carbonyl levels but increased levels of oxyhemoglobin and alpha-1-microglobulinin the *urine* of HD patients and HCs. Finally, Corey-Bloom et al. [25] described decreased *salivary* uric acid concentrations in female pre-HD and manifest HD patients and males with manifest HD in comparison to HCs (Supplementary Table S3).

## 3. Genetic Variants of Genes Related to Oxidative Stress in Patients with Huntington’s Disease

Weydt et al. [50], in a study involving 447 unrelated HD patients, showed an association between two haplotypes in the *PPARG coactivator 1 alpha* gene (*PPARGC1A* or *PGC-1alpha*, chromosome 4p15.2, Gene ID 10891, MIM 604517; this gene encodes a transcriptional coactivator that regulates the genes involved in energy metabolism) and the age at the onset of HD.

Berger et al. [51], studied the nine most common single nucleotide variants (SNVs) in the *8-oxo guanine DNA glycosylase* (*OGG1*, chromosome 3p25.3, gene ID 4968, MIM 601982; this gene encodes the enzyme responsible for the excision of 8-oxo guanine) and *XPC complex subunit*, *DNA damage recognition*, and *repair factor* genes (*XPC*, chromosome 3p25.1, gene ID 7508, MIM 613208; these genes encode a protein involved in global genome nucleotide excision repair) in 299 HD patients and 482 HCs and found an association between two *OGG1/XPC* haplotypes (related to altered protein levels via allele-specific mIR binding and a lower 8-oxoG repair activity) and younger age at the onset, which was independent of the number of CAG repeats within the *IT-15* gene.

Chang et al. [52], in a study involving 16 HD patients, four pre-HD asymptomatic carriers, and 20 HCs, measured the expression levels in the peripheral leukocytes of 17 candidate genes that were differentially expressed in a transgenic HD model. They found downregulation of four genes involved in oxidative stress in pre-HD and HD patients (*S-adenosyl-L-homocisteine hydrolase—AHCsY-*, *aconitase 2-ACO2-*, *3-oxoacid CoA transferase 1-OXCT1-*, and *adenylyl cyclase-associated protein 1-CAP1*) and downregulation of the *Uncoupling protein 2* (*UCP2*) gene in HD patients only.

## 4. Data from Experimental Models of Huntington’s Disease

Most of the data published to date on the possible role of oxidative stress in HD come from studies in different experimental models of this disease. The most important included neurotoxic models (e.g., administration to animals or use in culture media of excitotoxins, whose effects mimic those of HD, primarily 3-nitropropionic acid—3-NPA-, quinolinic acid, or malonic acid) and transgenic rodent models such as R6/1 and R6/2 mice (these carry only a fragment of the mutant human *HTT* gene), BAC-HD mice and rats, YAC-128 mice (artificial chromosome models), and the zQ175 and Hdh mice series (full-length knock-in models). There are also transgenic models of rats and large animals, including minipigs, sheep, and non-human primates, and even HD models in invertebrate animals (see [53,54,55,56,57] for review).

### 4.1. Lipid Peroxidation Markers

The results and methodology of published studies on lipid peroxidation markers in experimental models of HD, including MDA/TBARS, lipid peroxides, 4-hydroxyalkenals, and/or 4-hydroxynonenal (4-HNE) [7,24,58,59,60,61,62,63,64,65,66,67,68,69,70,71,72,73,74,75,76,77,78,79,80,81,82,83,84,85,86,87,88,89,90,91,92,93,94] are described in full detail in Supplementary Table S4. Most of them have shown increased levels of these markers in the (1) brain tissues [59,60,61,62,63,64,65,66,67,69,70,71,72,73,74,75,76,77,78], striatal and cortical synaptosomes [58], and plasma [65] of rats receiving intraperitoneal 3-NPA compared to those receiving vehicle controls; (2) murine neuroblastoma cells in culture [64] or striatal slices [88] incubated with 3-NPA compared to vehicle controls; (3) brain tissue of rats after intrastriatal injection with quinolinic acid [79,80,81,83,84] of malonic acid [82] compared to vehicle controls; (4) brain homogenates, brain synaptic vesicles, or striatal slices exposed to quinolinic acid [85,86,87,88]; (5) brain homogenates of different types of mice transgenic for HD compared to the wild-type cells [7,24,89,90,91]; and (6) cultures of striatal cell lines transgenic for HD compared to wild-type cells [94].

In contrast, a study on brain homogenates from the striatum and cerebellum of rats injected intraperitoneally with 3-NPA [68], one on the cerebellum, cerebral cortex, prefrontal cortex, hippocampus, and striatum in the yeast artificial chromosome 128 (YAC 128) line of transgenic mice [92], and other one on the frontal cortex, basal ganglia, and peripheral blood mononuclear cells (PBMCs) [93] showed similar MDA concentrations regarding their respective controls.

### 4.2. Protein Oxidation Markers

Supplementary Table S4 summarizes the results and methodology of studies addressing protein carbonyl concentrations in experimental models of HD [12,48,65,66,70,71,72,77,83,84,92,95,96,97,98,99,100,101]. Most of these studies have shown increased protein carbonyl levels in the brain tissues [60,65,66,68,70,71,72,77,81], striatal synaptosomes [48,95,96], and plasma [65] of rats injected with intraperitoneal 3-NPA and in the brain tissue of rats after intrastriatal injection of quinolinic acid [83,84,98,99] compared to their respective controls.

Several studies have shown increased protein carbonyl levels in the brain tissues [12,100,101], liver [101], and muscle [101] of different models of mice transgenic for HD.

Souza et al. [97] failed to find increased protein carbonyl concentrations in the striatum of rats treated with intraperitoneal 3-NPA, and Brocardo et al. [92] found similar protein carbonyl levels in homogenates from several brain areas of YAC-128 transgenic mice compared to wild-type controls.

### 4.3. DNA Oxidation Markers

The results and methodology of studies addressing concentrations of DNA oxidation markers, mainly 8-hydroxy-deoxyguanosine (OH^8^dG) and 8-oxo guanine (8-oxoG) in the experimental models of HD, are summarized in Supplementary Table S4 [79,91,93,102,103,104]. OH^8^dG and 8-oxoG have been found to increase in slices from the striatum, cerebral cortex, and cerebellum of mice after intraperitoneal administration of 3-NPA compared to vehicle controls [102]. Transgenic mice for *Sod2* gene mutations were more susceptible to DNA oxidation (assessed by OH^8^dG) than wild-type after 3-NPA.

OH^8^dG has been found to increase in the brain tissue of rats injected intrastriatal with quinolinic acid compared to that of vehicle controls [79] and in different types of transgenic mice models for HD compared to wild-type models [91,102]. Finally, Askeland et al. [93] described increased 8-oxoG in the frontal cortex, basal ganglia, and PBMCs of minipigs transgenic for HD.

### 4.4. Nitrosative and Nitrosidative Stress Markers

Supplementary Table S4 summarizes the results and methodology of studies addressing the concentrations of nitrites or nitrites + nitrates (nitric oxide production) and other markers of nitrosative stress in different experimental models of HD [22,61,62,63,64,66,67,68,69,71,72,73,74,75,80,82,83,84,87,103,105,106,107,108,109]. The majority of studies described increased nitrite concentrations and increased NO production in brain homogenates of mice treated with 3-NPA in comparison to mice treated with vehicle controls [61,62,63,66,67,68,69,71,72,73,74,75], except for Chang et al. [105], who described decreased NO production in brain homogenates from the cortex, striatum, and hippocampus and similar NO production in the plasma of rats treated with 3-NPA compared to those treated with vehicle controls. Tasset et al. [64] found increased nitrite concentrations in murine neuroblastoma cells incubated with 3-NPA compared to those incubated with the vehicle controls. Intrastriatal injections of quinolinic acid or malonic acid in rats caused increased brain nitrite levels [80,81,82,84], nitrotyrosine concentrations [87], and nitric oxide synthase (NOS) activity [87].

Increased production of NO [23] and nitrotyrosine [103] and increased NOS activity have been described in different models of transgenic animals [106]. Finally, NOS mRNA and protein expression increased in the striatum of rats after the intraperitoneal administration of 3-NPA [107] and after intrastriatal injection of quinolinic acid [108] and in striatal slices incubated with 3-NPA [109].

### 4.5. Global Oxidative Stress Markers and Trace Metals and Related Proteins

The results and methodology of studies addressing global stress markers, trace metals, and proteins related to trace metals are described in full detail in Supplementary Table S4 [19,21,40,60,63,68,70,76,79,81,86,88,94,97,99,103,110,111,112,113,114,115,116,117,118,119].

Reactive oxygen substances (ROS) production has been found to increase, compared to vehicle controls, in brain homogenates from rats after the intraperitoneal administration of 3-NPA [63,68,70,76,97] or after intrastriatal injection with quinolinic acid [79,99], as well as in striatal slices of rats incubated with 3-NPA, quinolinic acid or both neurotoxins [88]. These substances also increased in cellular cultures from striatum or striatal cell lines [94,110,112,113,114,115] and chromaffin cells [112] of different mice species transgenic for HD, in striatal progenitor cell lines expressing the *huntingtin* gene incubated with 3-NPA [116], and in rat pheochromocytoma cells expressing the *huntingtin* gene [117]. Superoxide anion production was increased in homogenates from the striatum after intrastriatal injection with quinolinic acid [81] and in heterozygote mice transgenic for the *Sod2* gene after the intraperitoneal administration of 3-NPA [103].

Total radical-trapping antioxidant potential (TRAP) and total antioxidant reactivity decreased in brain homogenates from rats treated previously with 3-NPA [60] and in homogenates from the cerebral cortex of rats incubated with quinolinic acid [86].

Iron [118,119] and ferritin [19] concentrations increased, while transferrin receptor [119], iron response proteins 1 and 2 (IRP-1 and IRP-2) [119], ferroportin [119], and aconitase 2 [42] decreased in brain tissues of several models of mice transgenic for HD compared to wild-type mice, and transferrin did not differ significantly between the two groups [119]. Finally, brain copper levels [118] and plasma selenium levels [21] were increased in mice transgenic for HD compared to wild-type mice.

### 4.6. Mitochondrial Respiratory Chain Complexes

The results and methodology of studies addressing mitochondrial dysfunction and/or the activities of mitochondrial respiratory chain complexes are described in full detail in Supplementary Table S4 [8,60,61,62,63,64,66,69,70,71,72,73,74,77,80,81,82,83,84,87,90,93,97,100,101,103,107,120,121]. The presence of *mitochondrial dysfunction* has been described in brain homogenates from rats treated with 3-NPA [62,63,69,70,71,72,74] or after intrastriatal injection with quinolinic [80,83] or malonic acid [82], in brain homogenates of rats incubated with 3-NPA [120] or quinolinic acid [87,120], and in vehicle controls.

*Nicotine adenine dinucleotide phosphatase* (*NADP*) *dehydrogenase* (*complex I*) activity has been found to decrease significantly compared to vehicle controls in most studies using brain homogenates of rats after the intraperitoneal administration of 3-NPA [61,62,69,70,71,72,73,74] and after intrastriatal injection with quinolinic acid [80,83,84]. In contrast, Sandhir et al. [63] described non-significant differences in rats injected intraperitoneally with 3-NPA, and Kalonia et al. [82] found no significant differences in rats injected intrastriatally with malonic acid [82] compared to vehicle controls. Askeland et al. [93] described similar *complex I activity* in minipigs transgenic for HD compared to wild-type animals.

*Succinate dehydrogenase* (*SDH*, *complex II*) *activity* decreased significantly in brain tissues of rats treated with 3-NPA [48,60,61,62,63,64,66,69,70,71,72,73,74,77,97,105,107,121] and rats receiving an intrastriatal injection of quinolinic [80,83,84] or malonic acid [82] compared to vehicle controls. While Johri et al. [90] reported a significant decrease in SDH in the muscle tissue of transgenic R6/2 mice, other authors described similar SDH activities between wild-type animals and in the brain, liver, and muscle of R6/2 transgenic mice [91] and in the frontal cortex, basal ganglia, and PBMCs of transgenic minipigs [93]. In the latter model, *cytochrome c oxidoreductase* (*complex III*) *activity* was also similar to that in wild-type control animals [93].

Compared to vehicle controls, *cytochrome oxidase* (*complex IV*) *activity* was decreased in brain tissues in rats after the intraperitoneal administration of 3-NPA [62,63,69,70,71,72,73,74] and intrastriatal injection of quinolinic acid [80,83,84] but not after intrastriatal malonic acid administration [82]. The only study addressing complex IV activity in brain tissues from transgenic R6/2 mice did not show significant differences compared to wild-type mice. Finally, *ATPase* (*complex V*) *activity* was decreased in brain homogenates of rats treated with intraperitoneal 3-NPA [69,70,71,72] or after intrastriatal injection of quinolinic acid [83].

### 4.7. Proteins, Enzymes, and Vitamins Protective Against Oxidative Stress

The results and methodology of studies addressing proteins, enzymes, and vitamins protective against oxidative stress processes are summarized in Supplementary Table S4 [12,21,22,23,24,46,47,48,60,61,62,64,65,66,67,68,69,70,71,72,73,74,78,80,82,83,84,85,86,91,93,94,97,98,99,101,105,107,110,111,113,114,117,118,122,123,124,125,126,127,128].

*Total superoxide dismutase* (*SOD*) *activity* was decreased in brain tissues from rats injected with 3-NPA [62,65,66,68,70,73,78,105] and rats injected intrastriatally with quinolinic acid [80,83,84,99,122], malonic acid [82], or 3-NPA [122], while it was found to be similar to in the plasma of rats treated with 3-NPA and that of vehicle controls [105]. In contrast, Túnez et al. [48] described an increased activity of this enzyme in striatal and cortical synaptosomes from rats injected with 3-NPA. Regarding brain homogenates from transgenic R6/1 mice, Santamaría et al. [122] described an increase in total SOD and *Cu/Zn-SOD* (*SOD-1*) activity but not in *Mn-SOD* (*SOD-2*) activity after a 19-week period; they also found a decrease in these factors after a 35-week period. Fox et al. [118] described non-significant differences in total brain SOD activity in R6/2 transgenic mice compared to wild-type controls, and Dominah et al. [94] found an increase in total SOD activity in a mutant striatal cell line, compared to wild-type controls, after incubation with an organophosphate compound. In addition, *SOD-1 activity* was decreased in brain homogenates of rats after striatal injection of quinolinic acid or 3-NPA, while *SOD-2 activity* showed a decrease only after 3-NPA [122]. *SOD-1 mRNA expression* increased in transgenic mice with HD [123], *SOD-2 activity* was increased in the brain, liver, and muscle homogenates from transgenic R6/2 mice [101], and *SOD-2 protein* increased in striatal but not cortical synaptosomes of transgenic R6/2 mice in comparison to wild-type mice [110].

*Glutathione peroxidase* (*GPx*) activity decreased in brain homogenates of rats treated with intraperitoneal 3-NPA [60,65,97], in murine neuroblastoma cell cultures [64], and brain homogenates of rats injected intrastriatally with quinolinic acid [83,84], compared to vehicle controls. In contrast, it has been found to increase in striatal cell lines transgenic for HD compared to wild-type cells [113]. Similarly, *GPx-1 expression* has been found to increase, compared to wild-type cells, in iPSCs from fibroblasts of YAC128 HD transgenic mice [123].

*Catalase* (*CAT*) *activity* in rat brains after intraperitoneal injection of 3-NPA has been found to be decreased [60,62,65,66,67,71,72,73,74,75], similar to vehicle controls [68], and increased in murine neuroblastoma cells after incubation with this toxin [64]. Intrastriatal administration of quinolinic [83,84,99] or malonic acid [84] in rats also caused a decrease in CAT activity.

*Reduced glutathione* (*GSH*) concentrations were decreased in brain homogenates from rats treated with 3-NPA compared to rats treated with vehicle [60,61,64,65,66,67,70,71,72,73,74,75,78], in murine neuroblastoma cells after incubation with this substance [64], in rats after intrastriatal injection with quinolinic [80,83,84,86] or malonic acid [82], and in brain cortex homogenates incubated with quinolinic acid [86]. Brain homogenates [21,91,127] and cellular cultures [94,113,114] of different species of mice transgenic for HD, spontaneously [21,91,113,114,127] or after incubation with an organophosphate compound [94], have shown decreased GSH concentrations compared to wild-type mice, while a study on primary chromaffin cells from adrenal medulla showed similar concentrations [111].

*Oxidized glutathione* (*GSSG*) concentrations were found to increase in the brain of rats treated with an intrastriatal injection of quinolinic [80] and malonic acid [82], in some species of mice transgenic for HD [21,127], and in cultures of striatal cells [113] and chromaffin cells from the adrenal medulla [111] of certain species of mice transgenic for HD in comparison to wild-type mice. The results of studies on *glutathione reductase* (*GR*) *activity* [65,83,97,113], *GR mRNA*, and *proteins* [91] in different experimental models are inconsistent. *Glutathione-S-transferase* (*GST*) *activity* in brain homogenates after 3-NPA is decreased [67] or similar to that of controls [97] and decreased in brain homogenates of rats after intrastriatal injection with quinolinic acid [83]. *Glutathione synthetase* is decreased in cell cultures of striatal cells from mice transgenic for HD [113].

Data from studies addressing the concentrations or activities of other antioxidants (described in full detail in Supplementary Table S4) include the following:

1. Decrease in glucose-6-phosphate dehydrogenase (G6PD) and 6 phosphogluconate dehydrogenase (6PGD) activities in striatal cell lines of transgenic mice for HD [113].
2. Decrease in glyceraldehyde-3-phosphate-dehydrogenase (GADPH) in striatal slices of mice treated with intraperitoneal 3-NPA compared to vehicle controls [107].
3. Decrease in glycerophosphocholine phosphodiesterase 1 (GPCPD1) expression in brain homogenates [24] and in glycogen synthase kinase-3β (GSK-3β) expression in primary neuronal and astrocytic cultures [23] of transgenic R6/2 mice compared to wild-type mice.
4. Decrease in monoamine oxidase A (MAO-A) mRNA and activity in cell cultures of mice transgenic for HD compared to wild-type mice [46].
5. Non-significant differences in thioredoxin reductase (TRR) activity in brain homogenates of rats treated with intraperitoneal 3-NPA compared to vehicle controls [68].
6. Increased expression of peroxiredoxin-1 (Prx-1) in cellular cultures of iPSCs from fibroblasts of YAC128 transgenic mice [123].
7. Significant increase in heme oxygenase-1 (HO-1) mRNA and protein expression and HO-1 activity in rats after intrastriatal injection of quinolinic acid compared to vehicle controls [98] and a significant decrease in HO-1 mRNA and protein expression in mice transgenic for HD compared to wild-type mice [91].
8. Significant increase in NADPH oxidase (NOX) activity in cellular cultures of a transgenic model of HD in mice after incubation with an organophosphate compound, compared to the wild type [94].
9. Significant decrease in pyridoxal kinase (PDXK) [12,22] and pyridoxal-5-phosphate (PLP) [12] activities in the brain of mice transgenic for HD compared to the wild type.
10. Significant decrease in vitamin E concentrations in brain homogenates and plasma from rats following intraperitoneal 3-NPA [65] or in brain homogenates from rats following intrastriatal quinolinic acid injection [83,84] compared to vehicle controls.
11. Significant decrease in vitamin C levels in brain homogenates [65] and plasma [65,105] from rats following intraperitoneal 3-NPA, in brain homogenates of rats after intrastriatal quinolinic acid injection [83,84] compared to vehicle controls, and in transgenic R6/2 mice compared to wild-type mice [128].
12. Significant decrease in total [67] and non-protein thiol [97] concentrations in brain homogenates of rats after intraperitoneal 3-NPA and in non-protein thiol levels in brain homogenates from rats after intrastriatal injection of quinolinic acid [99], compared to vehicle controls.

## 5. Discussion and Conclusions

**Figure 1:**
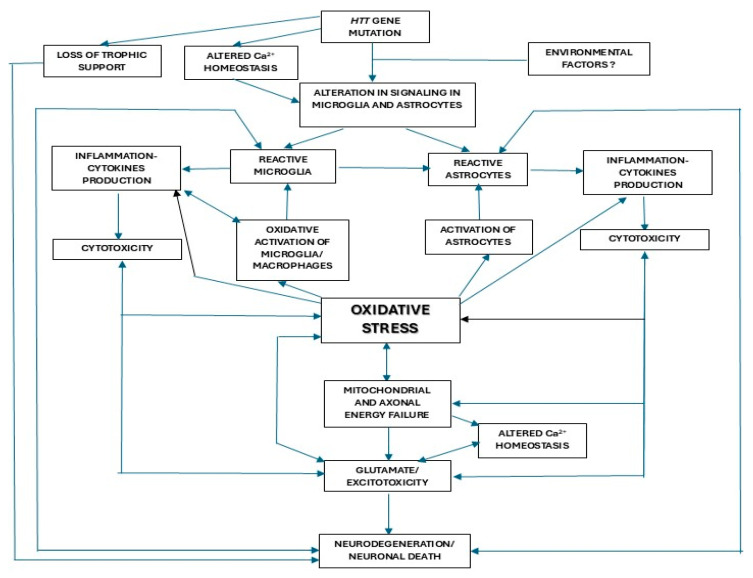
Possible interactions between the different pathogenic mechanisms proposed for Huntington’s disease.

The pathogenesis of HD is not well known but has been suggested to play a role in several factors, including, among others, excitotoxicity, autophagic dysregulations, protein accumulation and aggregation in organelles, loss of trophic support, and oxidative stress [129]. In Figure 1, we demonstrate the possible interactions between the different mechanisms proposed in the etiopathogenesis of HD, including oxidative stress.

The possible role of oxidative stress in the pathogenesis of HD has been suggested by studies in humans with HD (mainly for studies performed in brain samples from autopsies and plasma or serum; surprisingly, studies on CSF are scarce) and, above all, by studies in different experimental models of HD, such as the neurotoxic models of 3-NPA, quinolinic, and malonic acid, animals (especially mice) transgenic for HD, and cellular cultures.

**Figure 2:**
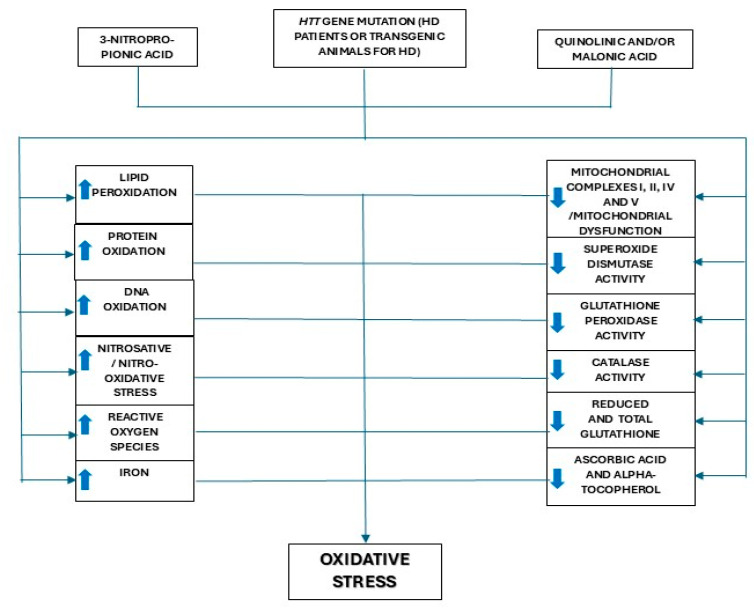
Summary of the most important changes related to oxidative stress reported in Huntington’s disease patients and experimental models of Huntington’s disease.

The most important data obtained in the studies addressing oxidative stress markers in patients with HD or experimental models of this disease, which have been summarized in Figure 2, include the following:

1. Increased levels of markers of lipid peroxidation in most studies performed in experimental models and in the plasma of HD patients (studies in the brain are scarce and not conclusive; lipid peroxidation markers were increased in a meta-analysis involving a large number of patients [48]).
2. Increased levels of markers of protein and DNA oxidation in most studies performed in experimental models of HD, with inconsistent results for brain samples from HD patients (although a meta-analysis showed increased plasma OH^8^dG concentrations [48]).
3. Increased markers of nitrosative and nitro-oxidative stress in most studies performed in experimental models of HD, while in humans with HD, a study showed no differences in CSF concentration of nitrates + nitrites compared to HCs [49].
4. Increased ROS and superoxide anion production and a decrease in global markers of antioxidant status in experimental models of HD.
5. Increased iron levels in the striatum of patients with HD [15,16,17,18] and in transgenic R6/2 mice [119].
6. Presence of mitochondrial dysfunction and decreased brain complexes I, II, IV, and V of the mitochondrial respiratory chain activities in most of the studies performed using experimental models of HD and a limited number of studies on HD patients.
7. Significant decrease in total SOD, GPx, and CAT activities and GSH concentrations in most of the studies using experimental models. Although data obtained in studies with HD brain samples are not conclusive, a meta-analysis of studies on blood oxidative markers of HD patients compared to HCs showed increased GPx activity, similar SOD activity, and decreased GSH levels [48]
8. Significant decrease in brain concentrations of vitamins E and C in experimental models of HD.

## 6. Future Directions

As we have described in the previous sections in sufficient detail, numerous data from studies in different HD models suggest an important role of oxidative stress mechanisms in this disease. However, the data in patients with HD, especially for brain samples, are insufficient as few studies have been published in this regard. On the other hand, and strikingly, despite the easy accessibility of CSF, there are hardly any published studies on markers of oxidative stress in patients with HD.

To better understand the possible role of oxidative stress in HD, it is important to significantly increase the number of studies on oxidative stress markers in patients with HD compared to HCs, for which we propose the following conditions:

1. Design prospective and multicenter studies with a long-term follow-up period (at least 10 years).
2. Ensure the participation of a significant number of patients with a genetic–molecular diagnosis of HD, both symptomatic and presymptomatic, as well as a similar number of healthy individuals, matched by age and sex, who are not carriers of *HTT* gene mutations.
3. The participants of the two study groups (HD patients and HCs) involved in the study should only be included after ruling out situations that could influence the oxidative stress measurement parameters, such as therapy with steroids, diuretics, diphosphonate vitamins, calcium or mineral supplements, or drugs that could affect oxidative stress, obesity, undernutrition, pregnancy, oncologic diseases, acute infectious diseases, liver, kidney, thyroid, or parathyroid disease, a recent history of surgery of traumatisms, and atypical dietary habits (for example, diets consisting exclusively of one type of food, such as vegetables).
4. It would be desirable to collect plasma/serum, blood cells, and CSF for the analysis of multiple oxidative stress biomarkers, both in HD patients and in HCs, at the baseline and after 5 and 10 years of follow-up.
5. Patients with HD should undergo periodic clinical evaluations every 6 months to evaluate the severity and progression of HD, measured according to the Unified Huntington’s Disease Rating Scale [130].
6. A new collection of plasma/serum, CSF, and blood cells should be performed for the analysis of multiple oxidative stress biomarkers at the end of the follow-up period.
7. Finally, it would be desirable, in the event of death, to obtain a brain donation from patients with HD and healthy HCs to be able to examine the different parameters of oxidative stress in them.

## Data Availability

Not applicable.

## Supplementary Materials

The following supporting information can be downloaded at: https://www.mdpi.com/article/10.3390/biom15040527/s1, Table S1: Oxidative stress markers in the brain from patients with Huntington’s disease (HD) and healthy controls (HC); Table S2. Oxidative stress markers in serum/plasma from Huntington’s disease (HD) patients and healthy controls (HC); Table S3. Oxidative stress markers in blood cells, skin fibroblasts, cerebrospinal fluid, urine, and saliva from Huntington’s disease (HD) patients and healthy controls (HC). Table S4. Oxidative stress markers in experimental models of Huntington’s disease (HD).

## References

[1] Walker F.O. (2007). Huntington’s Disease. Lancet.

[2] Roos R.A. (2010). Huntington’s Disease: A Clinical Review. Orphanet J. Rare Dis..

[3] Cepeda C., Tong X.P. (2018). Huntington’s Disease: From Basic Science to Therapeutics. CNS Neurosci. Ther..

[4] Ferreira J.J., Rodrigues F.B., Duarte G.S., Mestre T.A., Bachoud-Levi A.C., Bentivoglio A.R., Burgunder J.M., Cardoso F., Claassen D.O., Landwehrmeyer G.B. (2022). An MDS Evidence-Based Review on Treatments for Huntington’s Disease. Mov. Disord..

[5] Pan L., Feigin A. (2021). Huntington’s Disease: New Frontiers in Therapeutics. Curr. Neurol. Neurosci. Rep..

[6] Alam Z.I., Halliwell B., Jenner P. (2000). No Evidence for Increased Oxidative Damage to Lipids, Proteins, or DNA in Huntington’s Disease. J. Neurochem..

[7] Lee J., Kosaras B., Del Signore S.J., Cormier K., McKee A., Ratan R.R., Kowall N.W., Ryu H. (2011). Modulation of Lipid Peroxidation and Mitochondrial Function Improves Neuropathology in Huntington’s Disease Mice. Acta Neuropathol..

[8] Kreilaus F., Spiro A.S., McLean C.A., Garner B., Jenner A.M. (2016). Evidence for Altered Cholesterol Metabolism in Huntington’s Disease Post Mortem Brain Tissue. Neuropathol. Appl. Neurobiol..

[9] Sorolla M.A., Reverter-Branchat G., Tamarit J., Ferrer I., Ros J., Cabiscol E. (2008). Proteomic and Oxidative Stress Analysis in Human Brain Samples of Huntington Disease. Free Radic. Biol. Med..

[10] Browne S.E., Bowling A.C., MacGarvey U., Baik M.J., Berger S.C., Muqit M.M., Bird E.D., Beal M.F. (1997). Oxidative Damage and Metabolic Dysfunction in Huntington’s Disease: Selective Vulnerability of the Basal Ganglia. Ann. Neurol..

[11] Polidori M.C., Mecocci P., Browne S.E., Senin U., Beal M.F. (1999). Oxidative Damage to Mitochondrial DNA in Huntington’s Disease Parietal Cortex. Neurosci. Lett..

[12] Sorolla M.A., Rodríguez-Colman M.J., Tamarit J., Ortega Z., Lucas J.J., Ferrer I., Ros J., Cabiscol E. (2010). Protein Oxidation in Huntington Disease Affects Energy Production and Vitamin B6 Metabolism. Free Radic. Biol. Med..

[13] Richards G., Messer J., Waldvogel H.J., Gibbons H.M., Dragunow M., Faull R.L., Saura J. (2011). Up-Regulation of the Isoenzymes MAO-A and MAO-B in the Human Basal Ganglia and Pons in Huntington’s Disease Revealed by Quantitative Enzyme Radioautography. Brain Res..

[14] Sian J., Dexter D.T., Lees A.J., Daniel S., Agid Y., Javoy-Agid F., Jenner P., Marsden C.D. (1994). Alterations in Glutathione Levels in Parkinson’s Disease and Other Neurodegenerative Disorders Affecting Basal Ganglia. Ann. Neurol..

[15] Scholefield M., Patassini S., Xu J., Cooper G.J.S. (2023). Widespread Selenium Deficiency in the Brain of Cases with Huntington’s Disease Presents a New Potential Therapeutic Target. EBioMedicine.

[16] Bartzokis G., Cummings J., Perlman S., Hance D.B., Mintz J. (1999). Increased Basal Ganglia Iron Levels in Huntington Disease. Arch. Neurol..

[17] Bartzokis G., Tishler T.A. (2000). MRI Evaluation of Basal Ganglia Ferritin Iron and Neurotoxicity in Alzheimer’s and Huntington’s Disease. Cell. Mol. Biol..

[18] Vymazal J., Klempír J., Jech R., Zidovská J., Syka M., Růzicka E., Roth J. (2007). MR Relaxometry in Huntington’s Disease: Correlation Between Imaging, Genetic and Clinical Parameters. J. Neurol. Sci..

[19] Simmons D.A., Casale M., Alcon B., Pham N., Narayan N., Lynch G. (2007). Ferritin Accumulation in Dystrophic Microglia is an Early Event in the Development of Huntington’s Disease. Glia.

[20] Loeffler D.A., LeWitt P.A., Juneau P.L., Sima P.A., Nguyen H.U., DeMaggio A.J., Brickman C.M., Brewer G.J., Dick R.D., Troyer M.D. (1996). Increased regional brain concentrations of ceruloplasmin in neurodegenerative disorders. Brain Res..

[21] Lu Z., Marks E., Chen J., Moline J., Barrows L., Raisbeck M., Volitakis I., Cherny R.A., Chopra V., Bush A.I. (2014). Altered selenium status in Huntington’s disease: Neuroprotection by selenite in the N171-82Q mouse model. Neurobiol. Dis..

[22] Sorolla M.A., Rodríguez-Colman M.J., Vall-Llaura N., Vived C., Fernández-Nogales M., Lucas J.J., Ferrer I., Cabiscol E. (2016). Impaired PLP-dependent metabolism in brain samples from Huntington disease patients and transgenic R6/1 mice. Metab. Brain Dis..

[23] L’Episcopo F., Drouin-Ouellet J., Tirolo C., Pulvirenti A., Giugno R., Testa N., Caniglia S., Serapide M.F., Cisbani G., Barker R.A. (2016). GSK-3β-induced Tau pathology drives hippocampal neuronal cell death in Huntington’s disease: Involvement of astrocyte-neuron interactions. Cell Death Dis..

[24] Chang K.H., Cheng M.L., Tang H.Y., Lin C.Y., Chen C.M. (2024). Dysregulation of choline metabolism and therapeutic potential of citicoline in Huntington’s disease. Aging Cell.

[25] Corey-Bloom J., Haque A., Aboufadel S., Snell C., Fischer R.S., Granger S.W., Granger D.A., Thomas E.A. (2020). Uric Acid as a Potential Peripheral Biomarker for Disease Features in Huntington’s Patients. Front. Neurosci..

[26] Stoy N., Mackay G.M., Forrest C.M., Christofides J., Egerton M., Stone T.W., Darlington L.G. (2005). Tryptophan metabolism and oxidative stress in patients with Huntington’s disease. J. Neurochem..

[27] Christofides J., Bridel M., Egerton M., Mackay G.M., Forrest C.M., Stoy N., Darlington L.G., Stone T.W. (2006). Blood 5-hydroxytryptamine, 5-hydroxyindoleacetic acid and melatonin levels in patients with either Huntington’s disease or chronic brain injury. J. Neurochem..

[28] Chen C.M., Wu Y.R., Cheng M.L., Liu J.L., Lee Y.M., Lee P.W., Soong B.W., Chiu D.T. (2007). Increased oxidative damage and mitochondrial abnormalities in the peripheral blood of Huntington’s disease patients. Biochem. Biophys. Res. Commun..

[29] Peña-Sánchez M., Riverón-Forment G., Zaldívar-Vaillant T., Soto-Lavastida A., Borrero-Sánchez J., Lara-Fernández G., Esteban-Hernández E.M., Hernández-Díaz Z., González-Quevedo A., Fernández-Almirall I. (2015). Association of status redox with demographic, clinical and imaging parameters in patients with Huntington’s disease. Clin. Biochem..

[30] Olsson M.G., Davidsson S., Muhammad Z.D., Lahiri N., Tabrizi S.J., Akerstrom B., Bjorkqvist M. (2012). Increased levels of hemoglobin and alpha1-microglobulin in Huntington’s disease. Front. Biosci. (Elite Ed.).

[31] Klepac N., Relja M., Klepac R., Hećimović S., Babić T., Trkulja V. (2007). Oxidative stress parameters in plasma of Huntington’s disease patients, asymptomatic Huntington’s disease gene carriers and healthy subjects: A cross-sectional study. J. Neurol..

[32] Duran R., Barrero F.J., Morales B., Luna J.D., Ramirez M., Vives F. (2010). Oxidative stress and plasma aminopeptidase activity in Huntington’s disease. J. Neural Transm..

[33] Túnez I., Sánchez-López F., Agüera E., Fernández-Bolaños R., Sánchez F.M., Tasset-Cuevas I. (2011). Important role of oxidative stress biomarkers in Huntington’s disease. J. Med. Chem..

[34] Biglan K.M., Dorsey E.R., Evans R.V., Ross C.A., Hersch S., Shoulson I., Matson W., Kieburtz K., Huntington Study Group Pre-2CARE Investigators (2012). Plasma 8-hydroxy-2′-deoxyguanosine Levels in Huntington Disease and Healthy Controls Treated with Coenzyme Q10. J. Huntingt. Dis..

[35] Long J.D., Matson W.R., Juhl A.R., Leavitt B.R., Paulsen J.S., PREDICT-HD Investigators and Coordinators of the Huntington Study Group (2012). 8OHdG as a marker for Huntington disease progression. Neurobiol. Dis..

[36] Sánchez-López F., Tasset I., Agüera E., Feijóo M., Fernández-Bolaños R., Sánchez F.M., Ruiz M.C., Cruz A.H., Gascón F., Túnez I. (2012). Oxidative stress and inflammation biomarkers in the blood of patients with Huntington’s disease. Neurol. Res..

[37] Kalliolia E., Silajdžić E., Nambron R., Hill N.R., Doshi A., Frost C., Watt H., Hindmarsh P., Björkqvist M., Warner T.T. (2014). Plasma melatonin is reduced in Huntington’s disease. Mov. Disord..

[38] Squadrone S., Brizio P., Abete M.C., Brusco A. (2020). Trace elements profile in the blood of Huntington’ disease patients. J. Trace Elem. Med. Biol..

[39] Ciancarelli I., De Amicis D., Di Massimo C., Di Scanno C., Pistarini C., D’Orazio N., Tozzi Ciancarelli M.G. (2014). Peripheral biomarkers of oxidative stress and their limited potential in evaluation of clinical features of Huntington’s patients. Biomarkers.

[40] Cuturic M., Abramson R.K., Moran R.R., Hardin J.W., Frank E.M., Sellers A.A. (2013). Serum carnitine levels and levocarnitine supplementation in institutionalized Huntington’s disease patients. Neurol. Sci..

[41] Liu C.S., Cheng W.L., Kuo S.J., Li J.Y., Soong B.W., Wei Y.H. (2008). Depletion of mitochondrial DNA in leukocytes of patients with poly-Q diseases. J. Neurol. Sci..

[42] Chen C.M., Wu Y.R., Chang K.H. (2017). Altered Aconitase 2 Activity in Huntington’s Disease Peripheral Blood Cells and Mouse Model Striatum. Int. J. Mol. Sci..

[43] Zanella A., Izzo C., Meola G., Mariani M., Colotti M.T., Silani V., Pellegata G., Scarlato G. (1980). Metabolic impairment and membrane abnormality in red cells from Huntington’s disease. J. Neurol. Sci..

[44] Jędrak P., Mozolewski P., Węgrzyn G., Więckowski M.R. (2018). Mitochondrial alterations accompanied by oxidative stress conditions in skin fibroblasts of Huntington’s disease patients. Metab. Brain Dis..

[45] del Hoyo P., García-Redondo A., de Bustos F., Molina J.A., Sayed Y., Alonso-Navarro H., Caballero L., Arenas J., Jiménez-Jiménez F.J. (2006). Oxidative stress in skin fibroblasts cultures of patients with Huntington’s disease. Neurochem. Res..

[46] Ooi J., Hayden M.R., Pouladi M.A. (2015). Inhibition of Excessive Monoamine Oxidase A/B Activity Protects Against Stress-induced Neuronal Death in Huntington Disease. Mol. Neurobiol..

[47] PerezGrovas-Saltijeral A., Ochoa-Morales A., Miranda-Duarte A., Martínez-Ruano L., Jara-Prado A., Camacho-Molina A., Hidalgo-Bravo A. (2020). Telomere length analysis on leukocytes derived from patients with Huntington Disease. Mech. Ageing Dev..

[48] Tang Q., Liu H., Shi X.J., Cheng Y. (2020). Blood Oxidative Stress Marker Aberrations in Patients with Huntington’s Disease: A Meta-Analysis Study. Oxid. Med. Cell. Longev..

[49] Milstien S., Sakai N., Brew B.J., Krieger C., Vickers J.H., Saito K., Heyes M.P. (1994). cerebrospinal fluid nitrite/nitrate levels in neurologic diseases. J. Neurochem..

[50] Weydt P., Soyal S.M., Gellera C., Didonato S., Weidinger C., Oberkofler H., Landwehrmeyer G.B., Patsch W. (2009). The gene coding for PGC-1alpha modifies age at onset in Huntington’s Disease. Mol. Neurodegener..

[51] Berger F., Vaslin L., Belin L., Asselain B., Forlani S., Humbert S., Durr A., Hall J. (2013). The impact of single-nucleotide polymorphisms (SNPs) in OGG1 and XPC on the age at onset of Huntington disease. Mutat. Res..

[52] Chang K.H., Chen Y.C., Wu Y.R., Lee W.F., Chen C.M. (2012). Downregulation of genes involved in metabolism and oxidative stress in the peripheral leukocytes of Huntington’s disease patients. PLoS ONE.

[53] Upadhayay S., Jamwal S., Kumar P. (2023). Animal models of Huntington’s disease and their applicability to novel drug discovery and development. Expert Opin. Drug Discov..

[54] Morton A.J. (2023). Sleep and Circadian Rhythm Dysfunction in Animal Models of Huntington’s Disease. J. Huntingt. Dis..

[55] Rana N., Kapil L., Singh C., Singh A. (2024). Modeling Huntington’s disease: An insight on in-vitro and in-vivo models. Behav. Brain Res..

[56] Nittari G., Roy P., Martinelli I., Bellitto V., Tomassoni D., Traini E., Tayebati S.K., Amenta F. (2023). Rodent Models of Huntington’s Disease: An Overview. Biomedicines.

[57] Han B., Liang W., Li X.J., Li S., Yan S., Tu Z. (2024). Large animal models for Huntington’s disease research. Zool. Res..

[58] Túnez I., Montilla P., Del Carmen Muñoz M., Feijóo M., Salcedo M. (2004). Protective effect of melatonin on 3-nitropropionic acid-induced oxidative stress in synaptosomes in an animal model of Huntington’s disease. J. Pineal Res..

[59] Yang L., Calingasan N.Y., Chen J., Ley J.J., Becker D.A., Beal M.F. (2005). A novel azulenyl nitrone antioxidant protects against MPTP and 3-nitropropionic acid neurotoxicities. Exp. Neurol..

[60] Túnez I., Drucker-Colín R., Jimena I., Medina F.J., Muñoz M.C., Peña J., Montilla P. (2006). Transcranial magnetic stimulation attenuates cell loss and oxidative damage in the striatum induced in the 3-nitropropionic model of Huntington’s disease. J. Neurochem..

[61] Kumar P., Padi S.S., Naidu P.S., Kumar A. (2006). Effect of resveratrol on 3-nitropropionic acid-induced biochemical and behavioural changes: Possible neuroprotective mechanisms. Behav. Pharmacol..

[62] Kumar P., Kumar A. (2009). Possible role of sertraline against 3-nitropropionic acid induced behavioral, oxidative stress and mitochondrial dysfunctions in rat brain. Prog. Neuropsychopharmacol. Biol. Psychiatry.

[63] Sandhir R., Mehrotra A., Kamboj S.S. (2010). Lycopene prevents 3-nitropropionic acid-induced mitochondrial oxidative stress and dysfunctions in nervous system. Neurochem. Int..

[64] Tasset I., Pontes A.J., Hinojosa A.J., de la Torre R., Túnez I. (2011). Olive oil reduces oxidative damage in a 3-nitropropionic acid-induced Huntington’s disease-like rat model. Nutr. Neurosci..

[65] Gopinath K., Prakash D., Sudhandiran G. (2011). Neuroprotective effect of naringin, a dietary flavonoid against 3-nitropropionic acid-induced neuronal apoptosis. Neurochem. Int..

[66] Bhateja D.K., Dhull D.K., Gill A., Sidhu A., Sharma S., Reddy B.V., Padi S.S. (2012). Peroxisome proliferator-activated receptor-α activation attenuates 3-nitropropionic acid induced behavioral and biochemical alterations in rats: Possible neuroprotective mechanisms. Eur. J. Pharmacol..

[67] Shivasharan B.D., Nagakannan P., Thippeswamy B.S., Veerapur V.P., Bansal P., Unnikrishnan M.K. (2013). Protective effect of *Calendula officinalis* Linn. flowers against 3-nitropropionic acid induced experimental Huntington’s disease in rats. Drug Chem. Toxicol..

[68] Denny Joseph K.M., Muralidhara (2013). Enhanced neuroprotective effect of fish oil in combination with quercetin against 3-nitropropionic acid induced oxidative stress in rat brain. Prog. Neuropsychopharmacol. Biol. Psychiatry.

[69] Binawade Y., Jagtap A. (2013). Neuroprotective effect of lutein against 3-nitropropionic acid-induced Huntington’s disease-like symptoms: Possible behavioral, biochemical, and cellular alterations. J. Med. Food.

[70] Sandhir R., Yadav A., Mehrotra A., Sunkaria A., Singh A., Sharma S. (2014). Curcumin nanoparticles attenuate neurochemical and neurobehavioral deficits in experimental model of Huntington’s disease. Neuromol. Med..

[71] Thangarajan S., Deivasigamani A., Natarajan S.S., Krishnan P., Mohanan S.K. (2014). Neuroprotective Activity of L-Theanine on 3-Nitropropionic Acid-Induced Neurotoxicity in Rat Striatum. Int. J. Neurosci..

[72] Thangarajan S., Ramachandran S., Krishnamurthy P. (2016). Chrysin Exerts Neuroprotective Effects against 3-Nitropropionic Acid Induced Behavioral Despair-Mitochondrial Dysfunction and Striatal Apoptosis via Upregulating Bcl-2 Gene and Downregulating Bax-Bad Genes in Male Wistar Rats. Biomed. Pharmacother..

[73] Gupta S., Sharma B. (2014). Pharmacological Benefit of I(1)-Imidazoline Receptors Activation and Nuclear Factor Kappa-B (NF-κB) Modulation in Experimental Huntington’s Disease. Brain Res. Bull..

[74] Hariharan A., Shetty S., Shirole T., Jagtap A.G. (2014). Potential of Protease Inhibitor in 3-Nitropropionic Acid Induced Huntington’s Disease Like Symptoms: Mitochondrial Dysfunction and Neurodegeneration. Neurotoxicology.

[75] Khan A., Jamwal S., Bijjem K.R., Prakash A., Kumar P. (2015). Neuroprotective Effect of Hemeoxygenase-1/Glycogen Synthase Kinase-3β Modulators in 3-Nitropropionic Acid-Induced Neurotoxicity in Rats. Neuroscience.

[76] Courtes A.A., Arantes L.P., Barcelos R.P., da Silva I.K., Boligon A.A., Athayde M.L., Puntel R.L., Soares F.A. (2015). Protective Effects of Aqueous Extract of *Luehea divaricata* against Behavioral and Oxidative Changes Induced by 3-Nitropropionic Acid in Rats. Evid.-Based Complement. Altern. Med..

[77] Silva-Palacios A., Colín-González A.L., López-Cervantes S.P., Zazueta C., Luna-López A., Santamaría A., Königsberg M. (2017). *Tert*-Buthylhydroquinone Pre-Conditioning Exerts Dual Effects in Old Female Rats Exposed to 3-Nitropropionic Acid. Redox Biol..

[78] Badini F., Bayrami A., Mirshekar M.A., Shahraki S., Fanaei H. (2024). Levothyroxine Attenuates Behavioral Impairment and Improves Oxidative Stress and Histological Alteration 3-Nitropropionic Acid Induced Experimental Huntington’s Disease in Rats. Behav. Brain Res..

[79] Ryu J.K., Choi H.B., McLarnon J.G. (2006). Combined Minocycline Plus Pyruvate Treatment Enhances Effects of Each Agent to Inhibit Inflammation, Oxidative Damage, and Neuronal Loss in an Excitotoxic Animal Model of Huntington’s Disease. Neuroscience.

[80] Kalonia H., Kumar P., Nehru B., Kumar A. (2009). Neuroprotective Effect of MK-801 against Intra-Striatal Quinolinic Acid Induced Behavioral, Oxidative Stress and Cellular Alterations in Rats. Indian J. Exp. Biol..

[81] Maldonado P.D., Molina-Jijón E., Villeda-Hernández J., Galván-Arzate S., Santamaría A., Pedraza-Chaverrí J. (2010). NAD(P)H Oxidase Contributes to Neurotoxicity in an Excitotoxic/Prooxidant Model of Huntington’s Disease in Rats: Protective Role of Apocynin. J. Neurosci. Res..

[82] Kalonia H., Kumar P., Kumar A. (2010). Targeting Oxidative Stress Attenuates Malonic Acid Induced Huntington Like Behavioral and Mitochondrial Alterations in Rats. Eur. J. Pharmacol..

[83] Sumathi T., Vedagiri A., Ramachandran S., Purushothaman B. (2018). Quinolinic Acid-Induced Huntington Disease-Like Symptoms Mitigated by Potent Free Radical Scavenger Edaravone—A Pilot Study on Neurobehavioral, Biochemical, and Histological Approach in Male Wistar Rats. J. Mol. Neurosci..

[84] Purushothaman B., Sumathi T. (2022). 5,6,7-Trihydroxy Flavone Armoured Neurodegeneration Caused by Quinolinic Acid Induced Huntington’s Like Disease in Rat Striatum—Reinstating the Level of Brain Neurotrophins with Special Reference to Cognitive-Socio Behaviour, Biochemical and Histopathological Aspects. Neurosci. Res..

[85] Pérez-De La Cruz V., González-Cortés C., Galván-Arzate S., Medina-Campos O.N., Pérez-Severiano F., Ali S.F., Pedraza-Chaverrí J., Santamaría A. (2005). Excitotoxic Brain Damage Involves Early Peroxynitrite Formation in a Model of Huntington’s Disease in Rats: Protective Role of Iron Porphyrinate 5,10,15,20-Tetrakis (4-Sulfonatophenyl)Porphyrinate Iron (III). Neuroscience.

[86] Leipnitz G., Schumacher C., Scussiato K., Dalcin K.B., Wannmacher C.M., Wyse A.T., Dutra-Filho C.S., Wajner M., Latini A. (2005). Quinolinic Acid Reduces the Antioxidant Defenses in Cerebral Cortex of Young Rats. Int. J. Dev. Neurosci..

[87] Pérez-De La Cruz V., González-Cortés C., Pedraza-Chaverrí J., Maldonado P.D., Andrés-Martínez L., Santamaría A. (2006). Protective Effect of S-Allylcysteine on 3-Nitropropionic Acid-Induced Lipid Peroxidation and Mitochondrial Dysfunction in Rat Brain Synaptosomes. Brain Res. Bull..

[88] Colle D., Hartwig J.M., Soares F.A., Farina M. (2012). Probucol Modulates Oxidative Stress and Excitotoxicity in Huntington’s Disease Models in vitro. Brain Res. Bull..

[89] Pérez-Severiano F., Ríos C., Segovia J. (2000). Striatal Oxidative Damage Parallels the Expression of a Neurological Phenotype in Mice Transgenic for the Mutation of Huntington’s Disease. Brain Res..

[90] Johri A., Calingasan N.Y., Hennessey T.M., Sharma A., Yang L., Wille E., Chandra A., Beal M.F. (2012). Pharmacologic Activation of Mitochondrial Biogenesis Exerts Widespread Beneficial Effects in a Transgenic Mouse Model of Huntington’s Disease. Hum. Mol. Genet..

[91] Chandra A., Sharma A., Calingasan N.Y., White J.M., Shurubor Y., Yang X.W., Beal M.F., Johri A. (2016). Enhanced Mitochondrial Biogenesis Ameliorates Disease Phenotype in a Full-Length Mouse Model of Huntington’s Disease. Hum. Mol. Genet..

[92] Brocardo P.S., McGinnis E., Christie B.R., Gil-Mohapel J. (2016). Time-Course Analysis of Protein and Lipid Oxidation in the Brains of Yac128 Huntington’s Disease Transgenic Mice. Rejuvenation Res..

[93] Askeland G., Rodinova M., Štufková H., Dosoudilova Z., Baxa M., Smatlikova P., Bohuslavova B., Klempir J., Nguyen T.D., Kuśnierczyk A. (2018). A Transgenic Minipig Model of Huntington’s Disease Shows Early Signs of Behavioral and Molecular Pathologies. Dis. Model. Mech..

[94] Dominah G.A., McMinimy R.A., Kallon S., Kwakye G.F. (2017). Acute Exposure to Chlorpyrifos Caused NADPH Oxidase Mediated Oxidative Stress and Neurotoxicity in a Striatal Cell Model of Huntington’s Disease. Neurotoxicology.

[95] La Fontaine M.A., Geddes J.W., Banks A., Butterfield D.A. (2000). 3-Nitropropionic Acid Induced in vivo Protein Oxidation in Striatal and Cortical Synaptosomes: Insights into Huntington’s Disease. Brain Res..

[96] Fontaine M.A., Geddes J.W., Banks A., Butterfield D.A. (2000). Effect of Exogenous and Endogenous Antioxidants on 3-Nitropionic Acid-Induced in vivo Oxidative Stress and Striatal Lesions: Insights into Huntington’s Disease. J. Neurochem..

[97] Souza L.C., Wilhelm E.A., Bortolatto C.F., Nogueira C.W., Boeira S.P., Jesse C.R. (2014). Involvement of mGlu5 receptor in 3-nitropropionic acid-induced oxidative stress in rat striatum. Neurol. Res..

[98] Colín-González A.L., Orozco-Ibarra M., Chánez-Cárdenas M.E., Rangel-López E., Santamaría A., Pedraza-Chaverri J., Barrera-Oviedo D., Maldonado P.D. (2013). Heme oxygenase-1 (HO-1) upregulation delays morphological and oxidative damage induced in an excitotoxic/pro-oxidant model in the rat striatum. Neuroscience.

[99] Antunes Wilhelm E., Ricardo Jesse C., Folharini Bortolatto C., Wayne Nogueira C. (2013). Correlations between behavioural and oxidative parameters in a rat quinolinic acid model of Huntington’s disease: Protective effect of melatonin. Eur. J. Pharmacol..

[100] Lou S., Lepak V.C., Eberly L.E., Roth B., Cui W., Zhu X.H., Öz G., Dubinsky J.M. (2016). Oxygen consumption deficit in Huntington disease mouse brain under metabolic stress. Hum. Mol. Genet..

[101] Pinho B.R., Duarte A.I., Canas P.M., Moreira P.I., Murphy M.P., Oliveira J.M.A. (2020). The interplay between redox signalling and proteostasis in neurodegeneration: In vivo effects of a mitochondria-targeted antioxidant in Huntington’s disease mice. Free Radic. Biol. Med..

[102] Acevedo-Torres K., Berríos L., Rosario N., Dufault V., Skatchkov S., Eaton M.J., Torres-Ramos C.A., Ayala-Torres S. (2009). Mitochondrial DNA damage is a hallmark of chemically induced and the R6/2 transgenic model of Huntington’s disease. DNA Repair.

[103] Kim G.W., Chan P.H. (2002). Involvement of superoxide in excitotoxicity and DNA fragmentation in striatal vulnerability in mice after treatment with the mitochondrial toxin, 3-nitropropionic acid. J. Cereb. Blood Flow Metab..

[104] Bogdanov M.B., Andreassen O.A., Dedeoglu A., Ferrante R.J., Beal M.F. (2001). Increased oxidative damage to DNA in a transgenic mouse model of Huntington’s disease. J. Neurochem..

[105] Chang K.L., New L.S., Mal M., Goh C.W., Aw C.C., Browne E.R., Chan E.C. (2011). Metabolic profiling of 3-nitropropionic acid early-stage Huntington’s disease rat model using gas chromatography time-of-flight mass spectrometry. J. Proteome Res..

[106] Pérez-Severiano F., Escalante B., Vergara P., Ríos C., Segovia J. (2002). Age-dependent changes in nitric oxide synthase activity and protein expression in striata of mice transgenic for the Huntington’s disease mutation. Brain Res..

[107] Jang M., Cho I.H. (2016). Sulforaphane Ameliorates 3-Nitropropionic Acid-Induced Striatal Toxicity by Activating the Keap1-Nrf2-ARE Pathway and Inhibiting the MAPKs and NF-κB Pathways. Mol. Neurobiol..

[108] Aguilera P., Chánez-Cárdenas M.E., Floriano-Sánchez E., Barrera D., Santamaría A., Sánchez-González D.J., Pérez-Severiano F., Pedraza-Chaverrí J., Jiménez P.D. (2007). Time-related changes in constitutive and inducible nitric oxide synthases in the rat striatum in a model of Huntington’s disease. Neurotoxicology.

[109] Napolitano M., Zei D., Centonze D., Palermo R., Bernardi G., Vacca A., Calabresi P., Gulino A. (2008). NF-kB/NOS cross-talk induced by mitochondrial complex II inhibition: Implications for Huntington’s disease. Neurosci. Lett..

[110] Petersen M.H., Willert C.W., Andersen J.V., Madsen M., Waagepetersen H.S., Skotte N.H., Nørremølle A. (2022). Progressive Mitochondrial Dysfunction of Striatal Synapses in R6/2 Mouse Model of Huntington’s Disease. J. Huntingt. Dis..

[111] Fernández A., Martínez-Ramírez C., Gómez A., de Diego A.M.G., Gandía L., Casarejos M.J., García A.G. (2023). Mitochondrial dysfunction in chromaffin cells from the R6/1 mouse model of Huntington’s disease: Impact on exocytosis and calcium current regulation. Neurobiol. Dis..

[112] Lim D., Fedrizzi L., Tartari M., Zuccato C., Cattaneo E., Brini M., Carafoli E. (2008). Calcium homeostasis and mitochondrial dysfunction in striatal neurons of Huntington disease. J. Biol. Chem..

[113] Ribeiro M., Rosenstock T.R., Cunha-Oliveira T., Ferreira I.L., Oliveira C.R., Rego A.C. (2012). Glutathione redox cycle dysregulation in Huntington’s disease knock-in striatal cells. Free Radic. Biol. Med..

[114] Frederick N.M., Bertho J., Patel K.K., Petr G.T., Bakradze E., Smith S.B., Rosenberg P.A. (2014). Dysregulation of system xc(-) expression induced by mutant huntingtin in a striatal neuronal cell line and in R6/2 mice. Neurochem. Int..

[115] Wang J.Q., Chen Q., Wang X., Wang Q.C., Wang Y., Cheng H.P., Guo C., Sun Q., Chen Q., Tang T.S. (2013). Dysregulation of mitochondrial calcium signaling and superoxide flashes cause mitochondrial genomic DNA damage in Huntington disease. J. Biol. Chem..

[116] Choi Y.J., Om J.Y., Kim N.H., Chang J.E., Park J.H., Kim J.Y., Lee H.J., Kim S.S., Chun W. (2013). Heat Shock Transcription Factor-1 Suppresses Apoptotic Cell Death and ROS Generation in 3-Nitropropionic Acid-Stimulated Striatal Cells. Mol. Cell. Biochem..

[117] Pruccoli L., Breda C., Teti G., Falconi M., Giorgini F., Tarozzi A. (2021). Esculetin Provides Neuroprotection against Mutant Huntingtin-Induced Toxicity in Huntington’s Disease Models. Pharmaceuticals.

[118] Fox J.H., Kama J.A., Lieberman G., Chopra R., Dorsey K., Chopra V., Volitakis I., Cherny R.A., Bush A.I., Hersch S. (2007). Mechanisms of Copper Ion-Mediated Huntington’s Disease Progression. PLoS ONE.

[119] Chen J., Marks E., Lai B., Zhang Z., Duce J.A., Lam L.Q., Volitakis I., Bush A.I., Hersch S., Fox J.H. (2013). Iron Accumulates in Huntington’s Disease Neurons: Protection by Deferoxamine. PLoS ONE.

[120] Colle D., Santos D.B., Hartwig J.M., Godoi M., Braga A.L., Farina M. (2013). Succinobucol versus Probucol: Higher Efficiency of Succinobucol in Mitigating 3-NP-Induced Brain Mitochondrial Dysfunction and Oxidative Stress in Vitro. Mitochondrion.

[121] Rosenstock T.R., Abílio V.C., Frussa-Filho R., Kiyomoto B.H., Smaili S.S. (2009). Old Mice Present Increased Levels of Succinate Dehydrogenase Activity and Lower Vulnerability to Dyskinetic Effects of 3-Nitropropionic Acid. Pharmacol. Biochem. Behav..

[122] Santamaría A., Pérez-Severiano F., Rodríguez-Martínez E., Maldonado P.D., Pedraza-Chaverri J., Ríos C., Segovia J. (2001). Comparative Analysis of Superoxide Dismutase Activity between Acute Pharmacological Models and a Transgenic Mouse Model of Huntington’s Disease. Neurochem. Res..

[123] Szlachcic W.J., Switonski P.M., Krzyzosiak W.J., Figlerowicz M., Figiel M. (2015). Huntington Disease iPSCs Show Early Molecular Changes in Intracellular Signaling, the Expression of Oxidative Stress Proteins and the p53 Pathway. Dis. Model. Mech..

[124] Maksimović I.D., Jovanović M.D., Colić M., Mihajlović R., Mićić D., Selaković V., Ninković M., Malicević Z., Rusić-Stojiljković M., Jovicić A. (2001). Oxidative Damage and Metabolic Dysfunction in Experimental Huntington’s Disease: Selective Vulnerability of the Striatum and Hippocampus. Vojnosanit. Pregl..

[125] Tkác I., Keene C.D., Pfeuffer J., Low W.C., Gruetter R. (2001). Metabolic Changes in Quinolinic Acid-Lesioned Rat Striatum Detected Non-Invasively by In Vivo ¹H NMR Spectroscopy. J. Neurosci. Res..

[126] Choo Y.S., Mao Z., Johnson G.V., Lesort M. (2005). Increased Glutathione Levels in Cortical and Striatal Mitochondria of the R6/2 Huntington’s Disease Mouse Model. Neurosci. Lett..

[127] Hong C., Seo H., Kwak M., Jeon J., Jang J., Jeong E.M., Myeong J., Hwang Y.J., Ha K., Kang M.J. (2015). Increased TRPC5 Glutathionylation Contributes to Striatal Neuron Loss in Huntington’s Disease. Brain.

[128] Rebec G.V., Barton S.J., Ennis M.D. (2002). Dysregulation of Ascorbate Release in the Striatum of Behaving Mice Expressing the Huntington’s Disease Gene. J. Neurosci..

[129] Shafie A., Ashour A.A., Anwar S., Anjum F., Hassan M.I. (2024). Exploring molecular mechanisms, therapeutic strategies, and clinical manifestations of Huntington’s disease. Arch. Pharm. Res..

[130] Huntington Study Group (1996). Unified Huntington’s Disease Rating Scale: Reliability and Consistency. Mov. Disord..

