# Supplementary material for: Oxidative Stress in Huntington’s Disease
Source: Biomolecules. 2025 Apr 4;15(4):527. doi: 10.3390/biom15040527 (PMC12025275; doi:10.3390/biom15040527)
Supplement: Supplementary file 1 [file biomolecules-15-00527-s001.zip › biomolecules-3541684-supplementary.pdf]

biomolecules

MDPI

Review

OXIDATIVE STRESS IN HUNTINGTON'S DISEASE – SUPPLEMENTARY MATERIAL

Félix Javier Jiménez-Jiménez MD PhD (1), Hortensia Alonso-Navarro MD PhD (1), Elena García-Martín MD PhD (2), Alba Cárcamo-Fonfría MD (1), María del Mar Caballero-Muñoz MD (1) and José A.G. Agúndez MD PhD (2).

1. Section of Neurology, Hospital Universitario del Sureste, Arganda del Rey, Madrid. E-mails fjavier.jimenez@salud.madrid.org (F.J.J-J), hortalon@yahoo.es (H. A-N), alba.carcamo@salud.madrid.org (A. C-F) and mcmunoz@salud.madrid.org (M.M. C-M)
2. Universidad de Extremadura, University Institute of Molecular Pathology Biomarkers, Cáceres. SPAIN. E-mails elenag@unex.es (E.G-M) and jagundez@unex.es (J.A.G-A)

ORCID IDs:
Félix Javier Jiménez-Jiménez 0000-0002-7558-7323
Hortensia Alonso-Navarro 0000-0001-8644-2210
Elena García-Martín 0000-0002-8094-046X
Alba Cárcamo-Fonfría 0000-0002-9928-6293
José A.G. Agúndez 0000-0001-6895-9160

* Correspondence: Félix Javier Jiménez-Jiménez, Section of Neurology, Hospital Universitario del Sureste, Ronda del Sur 10, E28500 Arganda del Rey (Madrid, SPAIN) TEL +34636968395 FAX +34913280704 , E-Mails fjavier.jimenez@salud.madrid.org; felix.jimenez@sen.es

Supplementary Table S1. Oxidative stress markers in the brain from patients with Huntington's disease (HD) and healthy controls (HC). ATP adenosine-triphosphate, CAT catalase; CYP46A1 cytochrome P450 46A1; DHCR 24 delta(24)sterol reductase; DNA deoxyribonucleic acid; F female; FDRI field-dependent relaxation rate; GADPH Gliceraldehyde-phosphate-dehydrogenase; GP globus pallidus; GPCPD1 Glycerophosphocholine phosphodiesterase 1; GPx glutathione peroxidase; GSH reduced glutathione; GSK-3β Glycogen synthase kinase-3β; GSSG oxidized glutathione; HC healthy controls; HD Huntington's disease; 4-HNE 4-hydroxynonenal, HPLC high performance liquid chromatography M male; MDA malonyldialdehyde; MAO-A monoamine oxidase A; MAO-B monoamine oxidase B; MRI magnetic resonance imaging; OHC hydroxycholesterol; OH^{8}dG 8-hydroxy-deoxyguanosine; PDXK Pyridoxal kinase; PLP pyridoxal 5'-phosphate; PRX peroxiredoxin; qPCR quantitative polymerase chain reaction; SNc susbsantia nigra compacta; SOD superoxide dismutase.

PARAMETER	AUTHOR, YEAR [REF]	COUNTRY	METHOD	STUDY SUBJECTS	MAIN FINDINGS
MDA	Alam et al., 2000 [6]	United Kingdom and Singapore	HPLC	10 HD (M:F 6:4, 58.1 ± 11.6 years) and 10 HC (M:F 7:3, 55.2 ± 9.6 years)	Non-significant difference between HD and HC in the caudate, putamen, and frontal cortex
4-hydroxy-nonenal (4-HNE)	Lee et al., 2011 [7]	United States of America	Immunohistochemistry	5 HD patients (M:F 5:0, 49-80 years) and 5 HC (M:F 3:2, 51-87 years)	Increased immunoreactivity in the striatum of HD patients
Total cholesterol, cholesterol oxidation products, and precursors	Kreilaus et al., 2016 [8]	Australia	Electrospray ionization mass spectrometry and gas chromatography-mass spectrometry	8 HD patients (M:F 8:5, 51-77 years) and 13 HC (M:F 8:5, 59-81 years)	60% decrease in 24(S)-hydroxycholesterol (OHC), 30% increase in 27-OHC and 100-200% increase in synthetic precursors (lathosterol, zymosterol, and desmosterol) in the putamen of HD. 3-fold reduction of 24-OHC in the caudate of HD. Increase in cholesterol oxidation products in the putamen of HD. No other significant changes in the caudate cerebellum, and frontal cortex
Cholesterol 24-hydroxylase (CYP46A1) and delta(24)-sterol reductase (DHCR24)	Kreilaus et al., 2016 [8]	Australia	Western blotting and qPCR	8 HD patients (M:F 8:5, 51-77 years) and 13 HC (M:F 8:5, 59-81 years)	10-fold reduction in the putamen of HD patients
Protein Carbonyls	Alam et al., 2000 [6]	United Kingdom and Singapore	Colorimetric method with dinitrophenyl-hydrazine	10 HD (M:F 6:4, 58.1 ± 11.6 years) and 10 HC (M:F 7:3, 55.2 ± 9.6 years)	Non-significant difference between HD and HC in the caudate, putamen, and frontal cortex
Protein Carbonyls	Sorolla et al., 2008 [9]	Spain	Colorimetric method with dinitrophenyl-hydrazine	8 HD patients (M:F 4:4, 28-72 years) and 8 HC (M:F 4:4, 24-73years)	Increased concentrations in the striatum and cortex of HD patients
Modified DNA bases, including 8-OH-guanine, xanthine, and hypoxanthine	Alam et al., 2000 [6]	United Kingdom and Singapore	Gas chromatography-mass spectrometry	10 HD (M:F 6:4, 58.1 ± 11.6 years) and 10 HC (M:F 7:3, 55.2 ± 9.6 years)	Non-significant difference between HD and HC in the caudate, putamen, and frontal cortex
8-hydroxy-2'-deoxyguano-sine (OH^{8}dG) in nuclear DNA	Browne et al., 1997 [10]	United States of America	HPLC with colorimetric detection	18 HD (M:F 8:8, mean age 64 years) and 29 HC (M:F 15:14, mean age 71 years).	Significant increase in the caudate of HD patients and non-significant differences compared to controls in the putamen, frontal and parietal cortices, and cerebellum

8-hydroxy-2′-deoxyguanosine (OH^8dG) in mitochondrial DNA	Polidori et al., 1999 [11]	Italy and the United States of America	HPLC with electrochemical detection	17 HD (64.0 ± 1.2 years) and 10 HC (76.6 ± 3.7 years) for parietal cortex, 22 HD (66.0 ± 2.1 years) and 15 HC (72.8 ± 3.0 years) for parietal cortex, and 6 HD (67.6 ± 1.7 years) and 5 HC (72.3 ± 6.5 years) for cerebellum. Sex not given	Significant increase in the parietal cortex and non-significant differences in the frontal cortex and cerebellum of HD patients compared to controls
Glyceraldehyde-phosphate-dehydrogenase (GADPH) activity	Browne et al., 1997 [10]	United States of America	Spectrophotometry	18 HD (M:F 8:8, mean age 64 years) and 29 HC (M:F 15:14, mean age 71 years).	Non-significant differences between HD and HC in caudate, putamen, cerebellum, and frontal and parietal cortices
Glyceraldehyde-phosphate-dehydrogenase (GADPH) activity	Sorolla et al., 2008 [12]	Spain	Spectrophotometry	8 HD patients (M:F 4:4, 28-72 years) and 8 HC (M:F 4:4, 24-73years)	Non-significant differences between HD and HC in the striatum and cortex
Piruvate kinase activity	Sorolla et al., 2008 [12]	Spain	Spectrophotometry	8 HD patients (M:F 4:4, 28-72 years) and 8 HC (M:F 4:4, 24-73years)	Non-significant differences between HD and HC in the striatum and cortex
Creatine kinase activity	Sorolla et al., 2008 [12]	Spain	Spectrophotometry	8 HD patients (M:F 4:4, 28-72 years) and 8 HC (M:F 4:4, 24-73years)	Significant decrease in the striatum and cortex of HD patients
MAO B activity	Browne et al., 1997 [10]	United States of America	Spectrophotometry	18 HD (M:F 8:8, mean age 64 years) and 29 HC (M:F 15:14, mean age 71 years).	Significant increase in the caudate, putamen, and frontal cortex of HD patients, and non-significant differences compared to HD in the parietal cortex
MAO B activity	Richards et al., 2011 [13]	Switzerland, New Zealand, and Spain	Quantitative radioautography	5 HD (M:F 2:3, 41-78 years) and 29 HC (M:F 15:14, mean age 71 years).	Significant increase in the putamen, ventral striatum, globus pallidus externus, and internus of the basal ganglia and in the insular cortex of HD patients. Correlation with the grade of disease severity
MAO-A activity	Richards et al., 2011 [13]	Switzerland, New Zealand, and Spain	Quantitative radioautography	5 HD (M:F 2:3, 41-78 years) and 29 HC (M:F 15:14, mean age 71 years).	Significant increase in the putamen and SNc, and the pons. Correlation with the grade of disease severity.
Mitochondrial enzymatic complexes I-IV	Browne et al., 1997 [10]	United States of America	Spectrophotometry	18 HD (M:F 8:8, mean age 64 years) and 29 HC (M:F 4:1, 48-67 years).	Significant decrease of complex II+III corrected by citrate synthase in the caudate and putamen, and of complex IV in the putamen of HD patients. Non-significant differences compared to HC in frontal and parietal cortices and cerebellum
Citrate synthase activity	Browne et al., 1997 [10]	United States of America	Spectrophotometry	18 HD (M:F 8:8, mean age 64 years) and 29 HC (M:F 15:14, mean age 71 years).	Significant decrease in the putamen and increase in the cerebellum of HD patients. Non-significant differences to HC in caudate and in frontal and parietal cortices
Citrate synthase activity	Sorolla et al., 2008 [12]	Spain	Spectrophotometry	8 HD patients (M:F 4:4, 28-72 years) and 8 HC (M:F 4:4, 24-73years)	Significant decrease in the striatum and cortex of HD patients
F0F1 ATP synthase activity	Sorolla et al., 2008 [12]	Spain	Spectrophotometry	8 HD patients (M:F 4:4, 28-72 years) and 8 HC (M:F 4:4, 24-73years)	Significant decrease in the striatum and cortex of HD patients

Total SOD	Sorolla et al., 2008 [9]	Spain	Spectrophotometry	8 HD patients (M:F 4:4, 28-72 years) and 8 HC (M:F 4:4, 24-73years)	Increased activity in the striatum and cortex of HD patients
Cu/Zn SOD	Browne et al., 1997 [10]	United States of America	Spectrophotometry	18 HD (M:F 8:8, mean age 64 years) and 29 HC (M:F 15:14, mean age 71 years).	Significant decrease in the parietal cortex and cerebellum of HD patients and non-significant differences compared to controls in the caudate, putamen, and frontal cortex
Mn-SOD	Browne et al., 1997 [10]	United States of America	Spectrophotometry	18 HD patients (M:F 8:8, mean age 64 years) and 29 HC (M:F 15:14, mean age 71 years).	Non-significant differences compared to controls in the parietal cortex and cerebellum
GPx 1 and GPx6	Sorolla et al., 2008 [9]	Spain	Spectrophotometry	8 HD patients (M:F 4:4, 28-72 years) and 8 HC (M:F 4:4, 24-73years)	Increased activity in the striatum and cortex of HD patients
CAT	Sorolla et al., 2008 [9]	Spain	Spectrophotometry	8 HD patients (M:F 4:4, 28-72 years) and 8 HC (M:F 4:4, 24-73years)	Increased activity in the striatum and cortex of HD patients
Reduced glutathione (GSH)	Sian et al., 1994 [14]	United Kingdom	HPLC	10 HD patients (M:F 7:3, age 52.9 ± 3.5 years) and 10 HC (H.M 9:1, age 50.1 ± 4.7 years)	Non-significant differences between HD and HC in the SNc, caudate nucleus and cerebral cortex
Oxidized glutathione (GSSG)	Sian et al., 1994 [14]	United Kingdom	Enzymatic spectrophotometry	10 HD patients (M:F 7:3, age 52.9 ± 3.5 years) and 10 HC (H.M 9:1, age 50.1 ± 4.7 years)	Significant reduction in the caudate (50%), and non-significant trend towards reduction of in the cerebral cortex of HD patients
PRX1, 2 and 6	Sorolla et al., 2008 [9]	Spain	Spectrophotometry	8 HD patients (M:F 4:4, 28-72 years) and 8 HC (M:F 4:4, 24-73years)	Increased activity in the striatum and cortex of HD patients
Iron	Scholefield et al., 2023 [15]	United Kingdom and New Zealand	Inductively-coupled plasma mass spectrometry	9 HD patients (M:F 6:3, mean 67.4 years) and 9 HC (M:F 6:3, mean 65.2 years)	Increased in pallidus of HD, and non-significant differences between HD and HC in the caudate, putamen, SNc, motor, sensory and entorhinal cortex, middle frontal and middle temporal gyrus, cingulate gyrus, hippocampus, and cerebellum
Iron	Bartzokis et al., 1999 [16] and 2000 [17]	United States of America	Iron concentration measurements "in vivo" by MRI (field-dependent relaxation rate - R_2 increase ór FDRI)	11 HD patients (M:F 7:4, 46.2 ± 13.1 years) and 27 HC (M:F 23:4, 44.8 ± 16,2 years). Sex not given	Increased in the caudate, putamen, and pallidus, and decreased in the white matter of patients with HD compared with HC
Iron	Vymazal et al., 2007 [18]	Czech Republic	Iron concentration measurements "in vivo" by MRI relaxometry with double-echo proton density and T2-weighted turbo spin echo sequence	34 HD patients (M:F 24:10, 50.1± 11.8 years) and 34 HC (M:F 24:10, 49.6± 13.3 years)	Shortening in T2 relaxation time in the pallidus of HD patients, suggesting an increase in iron-bound ferritin (which was correlated with the number of CAG triplets in left pallidus and left caudate) Increased T2 relaxation time in the frontal white matter
Ferritin	Simmons et al., 2007 [19]	United States of America	Double immunofluorescence labeling	17 HD patients (M:F 11:6, 55.0± 15.0 years) and 34 HC (M:F 6:2, 55.0 ± 19.0 years)	Significant increase in the microglia of striatum and cortex compared to HC

Ferritin (light chain)	Sorolla et al., 2008 [9]	Spain	Mass spectrometry	8 HD patients (M:F 4:4, 28-72 years) and 8 HC (M:F 4:4, 24-73 years)	Increased concentrations in the striatum and cortex of HD patients
Aconitase	Sorolla et al., 2008 [9]	Spain	Mass spectrometry	8 HD patients (M:F 4:4, 28-72 years) and 8 HC (M:F 4:4, 24-73 years)	Decreased activity in the striatum and cortex of HD patients
Copper	Loeffler et al., 1996 [20]	United States of America	Atomic absorption spectrophotometry	11 HD patients (67.5 ± 3.1 years) and 7 HC (75.7 ± 2.8years). Sex not given	Significant reduction in caudate and SNc in HD patients, and similar levels in the putamen
Copper	Scholefield et al., 2023 [15]	United Kingdom and New Zealand	Inductively-coupled plasma mass spectrometry	9 HD patients (M:F 6:3, mean 67.4 years) and 9 HC (M:F 6:3, mean 65.2 years)	Decreased in the cerebellum of HD, and non-significant differences between HD and HC in the putamen, SNc, motor, sensory and entorhinal cortex, middle frontal and middle temporal gyrus, cingulate gyrus, hippocampus, and cerebellum
Ceruloplasmin	Loeffler et al., 1996 [20]	United States of America	Immunocytochemical staining	11 HD patients (67.5 ± 3.1 years) and 7 HC (75.7 ± 2.8years). Sex not given	Significant increase in SNc, hippocampus, and parietal cortex of HD, and non-significant differences with controls in caudate, putamen, cerebellum, frontal cortex, and temporal cortex
Sodium	Scholefield et al., 2023 [15]	United Kingdom and New Zealand	Inductively-coupled plasma mass spectrometry	9 HD patients (M:F 6:3, mean 67.4 years) and 9 HC (M:F 6:3, mean 65.2 years)	Decreased in the pallidus, putamen, motor, sensory and entorhinal cortex, middle frontal and middle temporal gyrus, hippocampus, and cingulate gyrus of HD, and non-significant differences between HD and HC in SNc, and cerebellum
Potassium	Scholefield et al., 2023 [15]	United Kingdom and New Zealand	Inductively-coupled plasma mass spectrometry	9 HD patients (M:F 6:3, mean 67.4 years) and 9 HC (M:F 6:3, mean 65.2 years)	Decreased in the putamen, SNc, sensory cortex, cingulate gyrus and cerebellum, and cingulate gyrus of HD, and non-significant differences between HD and HC in the pallidus, motor cortex, middle frontal and middle temporal gyrus, and entorhinal cortex
Magnesium	Scholefield et al., 2023 [15]	United Kingdom and New Zealand	Inductively-coupled plasma mass spectrometry	9 HD patients (M:F 6:3, mean 67.4 years) and 9 HC (M:F 6:3, mean 65.2 years)	Decreased in the putamen of HD, and non-significant differences between HD and HC in the pallidus, SNc, motor, sensory and entorhinal cortex, middle frontal and middle temporal gyrus, cingulate gyrus, hippocampus, and cerebellum
Calcium	Scholefield et al., 2023 [15]	United Kingdom and New Zealand	Inductively-coupled plasma mass spectrometry	9 HD patients (M:F 6:3, mean 67.4 years) and 9 HC (M:F 6:3, mean 65.2 years)	Decreased in the putamen, motor cortex, frontal and middle temporal gyrus, and cingulate gyrus of HD, and non-significant differences between HD and HC in the pallidus, SNc, sensory and entorhinal cortex, hippocampus, and cerebellum
Zinc	Scholefield et al., 2023 [15]	United Kingdom and New Zealand	Inductively-coupled plasma mass spectrometry	9 HD patients (M:F 6:3, mean 67.4 years) and 9 HC (M:F 6:3, mean 65.2 years)	Decreased in the putamen, pallidus, middle frontal, and middle temporal gyrus of HD, and non-significant differences between HD and HC in the caudate, pallidus, SNc, motor, sensory and entorhinal cortex cingulate, gyrus, hippocampus, and cerebellum
Manganese	Scholefield et al., 2023 [15]	United Kingdom and New Zealand	Inductively-coupled plasma mass spectrometry	9 HD patients (M:F 6:3, mean 67.4 years) and 9 HC (M:F 6:3, mean 65.2 years)	Decreased in the SNc of HD, and non-significant differences between HD and HC in the putamen, pallidus, motor, sensory and entorhinal cortex, middle frontal and middle temporal gyrus, cingulate gyrus, hippocampus, and cerebellum

Selenium	Lu et al., 2014 [21]	United States of America and Australia	Inductively-coupled plasma mass spectrometry	25 HD patients (M:F 15:10, 59.0 ± 12.0 year) and 12 HC (M:F 5:7, 62.0 ± 12.0 years)	Decreased in putamen, dorsolateral prefrontal cortex, primary visual cortex, cingulate gyrus and cerebellum of HD. Non-significant differences compared to controls in SNc and GP
Selenium	Scholefield et al., 2023 [15]	United Kingdom and New Zealand	Inductively-coupled plasma mass spectrometry	9 HD patients (M:F 6:3, mean 67.4 years) and 9 HC (M:F 6:3, mean 65.2 years)	Decreased in the putamen, pallidus, SNc, motor, sensory and entorhinal cortex, middle frontal and middle temporal gyrus, cingulate gyrus, hippocampus, and cerebellum, of HD patients
Pyridoxal 5′-phosphate (PLP) and pyridoxal kinase (PDXK)	Sorolla et al., 2016 [22]	Spain	Western blot and HPLC coupled to an electrospray ionization quadrupole time-of-flight mass spectrometry	8 HD patients (M:F 4:4, 28-72 years) and 8 HC (M:F 4:4, 24-73years)	Significant increase of pyridoxal and decrease of PDXK in the striatum and cortex of HD patients
Glycogen synthase kinase-3β (GSK-3β) expression	L'Episcopo et al., 2016 [23]	Italy, Sweden, Canada, and the United Kingdom	Real-time quantitative reverse transcription PCRs (qPCRs) and immunohistochemistry	23 HD patients (M:F 18:5, 40-78 years) and 11 HC (M:F 8:3,56-75 years)	Increased expression of GSK-3β and its active metabolite pGSK-3β-Tyr216 in the hippocampus of HD patients
Glycerophosphocholine phosphodiesterase 1 (GPCPD1) expression	Chang et al., 2024 [24]	Taiwan	Real-time quantitative PCRs (qPCRs) and immunohistochemistry	10 HD patients (M:F 5:5, 48-69 years) and 11 HC (M:F 4:3,58-74 years)	Significant decrease in HD patients (cortex-Brodmann area 6 - and striatum)
Uric acid	Corey-Bloom et al., 2020 [25]	United States of America	Colorimetric enzymatic assay	10 HD patients (M:F 3:7, 59.6 ± 4.33 years) and 10 HC (M:F 3:7, 60.0 ± 4.32 years)	Significant decrease in HD patients (prefrontal cortex)

Supplementary Table S2. Oxidative stress markers in serum/plasma from Huntington's disease (HD) patients and healthy controls (HC). Advanced oxidation protein products; CAT catalase; DNA deoxyribonucleic acid; F female; GOS global oxidant status; GPx glutathione peroxidase; GSH glutathione; GSSG oxidized glutathione; GST glutathione transferase; HC healthy controls; 4-HNE 4-hydroxy-2-nonenal; M male; MDA malonyldialdehyde; NO nitric oxide; NSE neuronal specific enolase; OH-dG 8-hydroxy-deoxyguanosine; SOD superoxide-dismutase; TAC total antioxidant capacity; TBA thiobarbituric acid; TBARS thiobarbituric acid reactive substances.

PLASMA					
**PARAMETER**	**AUTHOR, YEAR [REF]**	**COUNTRY**	**METHOD**	**STUDY SUBJECTS**	**MAIN FINDINGS**
MDA/TBA	Stoy et al., 2005 [26] Christofides et al. 2006 [27]	United Kingdom	Spectrophotometry	11 HD patients (M:F 3:8) and 15 HC (M:F 4:15), age not given	Significant increase in HD patients
MDA/TBA	Chen et al., 2007 [28]	Taiwan	HPLC with fluorescence detection	16 HD patients (M:F 9:7, 4-69 years) and 36 HC (M:F 19:17, 38-65 years)	Significant increase in HD patients. Correlation with HD severity
MDA/TBA	Peña-Sánchez et al., 2007 [29]	Cuba	Colorimetric method	14 HD patients (M:F 6:8, 48.4±14.1 years) and 29 HC (M:F 17:12, 53.0±9.2 years)	Significant increase in HD patients
MDA/TBA	Olsson et al., 2021 [30]	Sweden and the United Kingdom	Spectrophotometry	113 HD patients (M:F 67:46, 23-79 years, 32 with premanifest HD) and 84 HC (M:F 36:48, 21-74 years)	Non-significant differences between HD patients and HC
4-hydroxynonenal (4-HNE)	Stoy et al., 2005 [26] Christofides et al. 2006 [27]	United Kingdom	Spectrophotometry	11 HD patients (M:F 3:8) and 15 HC (M:F 4:15), sex not given	Significant increase in HD patients
Lipid peroxides	Klepac et al., 2007 [31]	Croatia	Spectrophotometry	19 HD patients (M:F 14:5, 18-58 years) and 47 HC (M:F 31:16, 21-58 years). 11 HD gene asymptomatic carriers (M:F 6:5, 17-30 years) and 22 HC (M:F 12:10, 17- 31 years).	Significant increase in HD patients and in HD gene carriers compared to their respective controls. Lack of correlation with the number of CAG triplets
Lipid peroxides	Durán et al., 2010 [32]	Spain	Enzymatic bioanalysis	24 HD patients (M:F 10:14, 48.4 ± 2.4 years), 7 HD gene asymptomatic carriers (M:F 2:5, 34.4 ± 2.6 years) and 60 HC (M:F 30:30, 48.0 ± 2.4 years).	Significant increase in HD patients but not in asymptomatic HD carriers.
Protein carbonyls	Klepac et al., 2007 [31]	Croatia	Spectrophotometry	19 HD patients (M:F 14:5, 18-58 years) and 47 HC (M:F 31:16, 21-58 years). 11 HD gene asymptomatic carriers (M:F 6:5, 17-30 years) and 22 HC (M:F 12:10, 17- 31 years).	Significant increase in HD patients and in HD gene carriers compared to their respective controls. Lack of correlation with the number of CAG triplets
Protein carbonyls	Túnez et al., 2011 [33]	Spain	HPLC gel filtration	19 HD patients (M:F 9:10, 43.50 ± 10.62 years) and 19 HC (M:F 8:11, 42.23 ± 9.34 years)	Significant increase in HD patients

Protein carbonyls	Olsson et al., 2021 [30]	Sweden and the United Kingdom	Spectrophotometry	113 HD patients (M:F 67:46, 23-79 years, 32 with premanifest HD) and 84 HC (M:F 36:48, 21-74 years)	Non-significant differences between HD patients and HC
Advanced oxidation protein products (AOPP)	Peña-Sánchez et al., 2007 [29]	Cuba	Spectrophotometry	14 HD patients (M:F 6:8, 48.4±14.1 years) and 29 HC (M:F 17:12, 53.0±9.2 years)	Significant increase in HD patients. Correlation with age at onset and severity of HD.
8-hydroxy-deoxyguano-sine (OH^8dG)	Túnez et al., 2011 [33]	Spain	ELISA	19 HD patients (M:F 9:10, 43.50 ± 10.62 years) and 19 HC (M:F 8:11, 42.23 ± 9.34 21-58 years)	Significant increase in HD patients
8-hydroxy-deoxyguano-sine (OH^8dG)	Biglan et al., 2012 [34]	United States of America	HPLC with electrochemical detection	14 HD patients (M:F 8:6, 52.65 ± 8.61 years) and 6 HC (M:F 4:2, 51.57 ± 8,64 years)	Non-significant differences between HD patients and HC
8-hydroxy-deoxyguano-sine (OH^8dG)	Long et al., 2012 [35]	United States of America and Canada	Liquid Chromatography. Electrochemical Array	80 prodromal HD patients participating in the PREDICT_HD study, classified as low, middle, and high risk for HD, and controls (genetically negative) (N not given)	In a longitudinal follow-up, OH^8dG levels were lowest for the control group and highest for the high-risk group. The rate of increase of OH^8dG levels varied similarly.
Global oxidant status (GOS)	Túnez et al., 2011 [33]	Spain	Calculated from 7 oxidative stress parameters	19 HD patients (M:F 9:10, 43.50 ± 10.62 years) and 19 HC (M:F 8:11, 42.23 ± 9.34 21-58 years)	Significant increase in HD patients
Total antioxidant capacity (FRAP)	Peña-Sánchez et al., 2007 [29]	Cuba	Spectrophotometry	14 HD patients (M:F 6:8, 48.4±14.1 years) and 29 HC (M:F 17:12, 53.0±9.2 years)	Non-significant differences between HD patients and HC. Correlation with age at onset of HD
Total antioxidant capacity (TAC)	Túnez et al., 2011 [33]	Spain	ELISA	19 HD patients (M:F 9:10, 43.50 ± 10.62 years) and 19 HC (M:F 8:11, 42.23 ± 9.34 21-58 years)	Significant decrease in HD patients
Protein thiols	Peña-Sánchez et al., 2007 [29]	Cuba	Spectrophotometry	14 HD patients (M:F 6:8, 48.4±14.1 years) and 29 HC (M:F 17:12, 53.0±9.2 years)	Non-significant differences between HD patients and HC
Superoxide anion (O2*)	Klepac et al., 2007 [31]	Croatia	Spectrophotometry	19 HD patients (M:F 14:5, 18-58 years) and 47 HC (M:F 31:16, 21-58 years). 11 HD gene asymptomatic carriers (M:F 6:5, 17-30 years) and 22 HC (M:F 12:10, 17- 31 years).	Non-significant differences between HD patients and HD gene carriers compared to their respective controls. Lack of correlation with the number of CAG triplets
Total SOD activity	Peña-Sánchez et al., 2007 [29]	Cuba	Spectrophotometry	14 HD patients (M:F 6:8, 48.4±14.1 years) and 29 HC (M:F 17:12, 53.0±9.2 years)	Non-significant differences between HD patients and HC
Total SOD activity	Klepac et al., 2007 [31]	Croatia	Spectrophotometry	19 HD patients (M:F 14:5, 18-58 years) and 47 HC (M:F 31:16, 21-58 years). 11 HD gene asymptomatic	Non-significant differences between HD patients and HD gene carriers compared to their respective controls. Lack of correlation with the number of

				carriers (M:F 6:5, 17-30 years) and 22 HC (M:F 12:10, 17- 31 years).	CAG triplets
GPx activity	Peña-Sánchez et al., 2007 [29]	Cuba	Spectrophotometry	14 HD patients (M:F 6:8, 48.4±14.1 years) and 29 HC (M:F 17:12, 53.0±9.2 years)	Significant increase in HD patients
GPx activity	Klepac et al., 2007 [31]	Croatia	Spectrophotometry	19 HD patients (M:F 14:5, 18-58 years) and 47 HC (M:F 31:16, 21-58 years). 11 HD gene asymptomatic carriers (M:F 6:5, 17-30 years) and 22 HC (M:F 12:10, 17- 31 years).	Non-significant differences between HD patients and HD gene carriers compared to their respective controls. Lack of correlation with the number of CAG triplets
GR activity	Peña-Sánchez et al., 2007 [29]	Cuba	Spectrophotometry	14 HD patients (M:F 6:8, 48.4±14.1 years) and 29 HC (M:F 17:12, 53.0±9.2 years)	Significant decrease in HD patients
Total glutathione	Túnez et al., 2011 [33]	Spain	Enzymatic assay	19 HD patients (M:F 9:10, 43.50 ± 10.62 years) and 19 HC (M:F 8:11, 42.23 ± 9.34 21-58 years)	Non-significant differences between HD patients and HC.
GSH	Peña-Sánchez et al., 2007 [29]	Cuba	Spectrophotometry	14 HD patients (M:F 6:8, 48.4±14.1 years) and 29 HC (M:F 17:12, 53.0±9.2 years)	Non-significant differences between HD patients and HC. Correlation with caudate atrophy
GSH	Klepac et al., 2007 [31]	Croatia	Spectrophotometry	19 HD patients (M:F 14:5, 18-58 years) and 47 HC (M:F 31:16, 21-58 years). 11 HD gene asymptomatic carriers (M:F 6:5, 17-30 years) and 22 HC (M:F 12:10, 17- 31 years).	Significant decrease in HD patients and in HD gene carriers compared to their respective controls. Lack of correlation with the number of CAG triplets
GSH	Túnez et al., 2011 [33]	Spain	Enzymatic assay	19 HD patients (M:F 9:10, 43.50 ± 10.62 years) and 19 HC (M:F 8:11, 42.23 ± 9.34 21-58 years)	Significant decrease in HD patients
GSSG	Túnez et al., 2011 [33]	Spain	Enzymatic assay	19 HD patients (M:F 9:10, 43.50 ± 10.62 years) and 19 HC (M:F 8:11, 42.23 ± 9.34 21-58 years)	Significant increase in HD patients
CAT activity	Peña-Sánchez et al., 2007 [29]	Cuba	Spectrophotometry	14 HD patients (M:F 6:8, 48.4±14.1 years) and 29 HC (M:F 17:12, 53.0±9.2 years)	Non-significant differences between HD patients and HC
Myeloperoxidase (MPO) activity	Sánchez-López et al., 2012 [36]	Spain	ELISA	13 HD patients (M:F 6:8, 42.0±10.65 years) and 29 HC (M:F 4:6, 39.0±12.51 years)	Increased in MPO/WBC ratio in HD patients
Thioredoxin-1 (Trx-1)	Sánchez-López et al., 2012 [36]	Spain	ELISA	13 HD patients (M:F 6:8, 42.0±10.65 years) and 29 HC (M:F 4:6, 39.0±12.51 years)	Decreased in HD patients
Thioredoxin-reductase 1 (TrRD-1) activity	Sánchez-López et al., 2012 [36]	Spain	ELISA	13 HD patients (M:F 6:8, 42.0±10.65 years) and 29 HC (M:F 4:6, 39.0±12.51 years)	Decreased in HD patients

Lactate	Durán et al., 2010 [32]	Spain	Enzymatic bioanalysis	24 HD patients (M:F 10:14, 48.4 ± 2.4 years), 7 HD gene asymptomatic carriers (M:F 2:5, 34.4 ± 2.6 years) and 60 HC (M:F 30:30, 48.0 ± 2.4 years).	Significant increase in HD patients but not in asymptomatic HD carriers.
Aminopepti-dases activities	Durán et al., 2010 [32]	Spain	Fluorometric method	24 HD patients (M:F 10:14, 48.4 ± 2.4 years), 7 HD gene asymptomatic carriers (M:F 2:5, 34.4 ± 2.6 years) and 60 HC (M:F 30:30, 48.0 ± 2.4 years).	Significant increase in HD patients and in asymptomatic HD carriers.
Melatonin	Kalliolia et al., 2014 [37]	United Kingdom and Sweden	Radioimmunoanal ysis	13 HD patients (M:F 18:5, 42-70 years), 14 HD gene asymptomatic carriers (M:F 5:9, 39-58 years) and 15 HC (M:F 9:6, 29-69 years).	Significant decrease of mean and acrophase melatonin in HD, and a non-significant trend toward a decrease in presymptomatic HD patients
Oxyhemo-globin	Olsson et al., 2021 [30]	Sweden and the United Kingdom	ELISA	113 HD patients (M:F 67:46, 23-79 years, 32 with premanifest HD) and 84 HC (M:F 36:48, 21-74 years)	Non-significant differences between HD patients and HC
Alpha-1-microglobulin	Olsson et al., 2021 [30]	Sweden and the United Kingdom	Radioimmunoanal ysis	113 HD patients (M:F 67:46, 23-79 years, 32 with premanifest HD) and 84 HC (M:F 36:48, 21-74 years)	Non-significant differences between HD patients and HC
Uric acid	Corey-Bloom et al., 2020 [25]	United States of America	Colorimetric enzymatic assay	69 HD patients (M:F 29:40, 52.4 ± 13.5 years, 31 presymptomatic) and 84 HC (M:F 43:41,52.9 ± 16.0 years)	Significantly lower in female pre-HD and manifest HD patients compared to HC
Iron	Squadrone et al., 2020 [38]	Italy	Inductively coupled plasma mass spectrometry (ICP-MS	18 HD patients (M:F 10:8) and 18 HC (M:F 10:8). Age not given	Increased in HD patients
Copper	Squadrone et al., 2020 [38]	Italy	Inductively coupled plasma mass spectrometry (ICP-MS	18 HD patients (M:F 10:8) and 18 HC (M:F 10:8). Age not given	Non-significant differences between HD and HC
Manganese	Squadrone et al., 2020 [38]	Italy	Inductively coupled plasma mass spectrometry (ICP-MS	18 HD patients (M:F 10:8) and 18 HC (M:F 10:8). Age not given	Non-significant differences between HD and HC
Zinc	Squadrone et al., 2020 [38]	Italy	Inductively coupled plasma mass spectrometry (ICP-MS	18 HD patients (M:F 10:8) and 18 HC (M:F 10:8). Age not given	Increased in HD patients
Selenium	Lu et al., 2014 [21]	United States of America and Australia	Inductively-coupled plasma mass spectrometry	20 HD patients (M:F 10:10, 44.5 ± 2.50 years) and 22 HC (M:F 12:10, 47.0 ± 3.4 years)	Non-significant differences between HD and HC
Selenium	Squadrone et al., 2020 [38]	Italy	Inductively coupled plasma mass spectrometry (ICP-MS	18 HD patients (M:F 10:8) and 18 HC (M:F 10:8). Age not given	Increased in HD patients

Chromium	Squadrone et al., 2020 [38]	Italy	Inductively coupled plasma mass spectrometry (ICP-MS	18 HD patients (M:F 10:8) and 18 HC (M:F 10:8). Age not given	Increased in HD patients
Arsenic	Squadrone et al., 2020 [38]	Italy	Inductively coupled plasma mass spectrometry (ICP-MS	18 HD patients (M:F 10:8) and 18 HC (M:F 10:8). Age not given	Increased in HD patients
Lead	Squadrone et al., 2020 [38]	Italy	Inductively coupled plasma mass spectrometry (ICP-MS	18 HD patients (M:F 10:8) and 18 HC (M:F 10:8). Age not given	Decreased in HD patients
Vanadium	Squadrone et al., 2020 [38]	Italy	Inductively coupled plasma mass spectrometry (ICP-MS	18 HD patients (M:F 10:8) and 18 HC (M:F 10:8). Age not given	Decreased in HD patients
Antimony	Squadrone et al., 2020 [38]	Italy	Inductively coupled plasma mass spectrometry (ICP-MS	18 HD patients (M:F 10:8) and 18 HC (M:F 10:8). Age not given	Decreased in HD patients
Cadmium	Squadrone et al., 2020 [38]	Italy	Inductively coupled plasma mass spectrometry (ICP-MS	18 HD patients (M:F 10:8) and 18 HC (M:F 10:8). Age not given	Below the detection limit
Cobalt	Squadrone et al., 2020 [38]	Italy	Inductively coupled plasma mass spectrometry (ICP-MS	18 HD patients (M:F 10:8) and 18 HC (M:F 10:8). Age not given	Below the detection limit
Molybdenum	Squadrone et al., 2020 [38]	Italy	Inductively coupled plasma mass spectrometry (ICP-MS	18 HD patients (M:F 10:8) and 18 HC (M:F 10:8). Age not given	Below the detection limit
Nickel	Squadrone et al., 2020 [38]	Italy	Inductively coupled plasma mass spectrometry (ICP-MS	18 HD patients (M:F 10:8) and 18 HC (M:F 10:8). Age not given	Below the detection limit
Tin	Squadrone et al., 2020 [38]	Italy	Inductively coupled plasma mass spectrometry (ICP-MS	18 HD patients (M:F 10:8) and 18 HC (M:F 10:8). Age not given	Below the detection limit

SERUM

**PARAMETER**	**AUTHOR, YEAR [REF]**	**COUNTRY**	**METHOD**	**STUDY SUBJECTS**	**MAIN FINDINGS**
8-hydroxydeoxy-guanosine (OH^8dG)	Ciancarelli et al., 2014 [39]	Italy	Fast and sensitive immunoassay with an ELISA kit	13 HD patients (M:F 8:5, 51.1 ± 2.81 years) and 10 HC (M:F 4:6, 50.0 ± 3.6 years)	Increased in HD patients. Lack of correlation with age at onset, duration of HD, and functional disability
Cu/Zn SOD	Ciancarelli et al., 2014 [39]	Italy	ELISA kit	13 HD patients (M:F 8:5, 51.1 ± 2.81 years) and 10 HC (M:F 4:6, 50.0 ± 3.6 years)	Increased in HD patients. Lack of correlation with age at onset, duration of HD, and functional disability
Neuron specific enolase (NSE)	Ciancarelli et al., 2014 [39]	Italy	ELISA kit	13 HD patients (M:F 8:5, 51.1 ± 2.81 years) and 10 HC (M:F 4:6, 50.0 ± 3.6 years)	Increased in HD patients. Lack of correlation with age at onset, duration of HD, and functional disability

Carnitine	Cuturic et al., 2013 [40]	United States of America	Not specified	23 HD patients (M:F 15:8) compared with reference values. Age not given	26.1% of HD patients showed low serum carnitine levels

Supplementary Table S3. Oxidative stress markers in blood cells, skin fibroblasts, cerebrospinal fluid, urine, and saliva from Huntington's disease (HD) patients and healthy controls (HC)
ATP adenosin-tryphosphate; CAT catalase; CoQ_{10} coenzyme Q_{10}; DNA deoxyribonucleic acid; F female; GADPH Gliceraldehyde-phosphate-dehydrogenase; GPx glutathione peroxidase; GR glutathione reductase; GSH glutathione; GSSG oxidized glutathione; GST glutathione transferase; HC healthy controls; 4-HNE 4-hydroxy-2-nonenal; LDH lactic dehydrogenase; M male; MDA malonyldialdehyde; MAO-A monoamine oxidase A; mtDNA mitochondrial DNA; OH-dG 8-hydroxy-deoxyguanosine; ROS reactive oxygen species; SOD superoxide-dismutase; TrRD-1 Thioredoxin-reductase 1 (TrRD-1); Trx-1 Tioredoxin- 1; TBA thiobarbituric acid

LEUKOCYTES

PARAMETER	AUTHOR, YEAR [REF]	COUNTRY	METHOD	STUDY SUBJECTS	MAIN FINDINGS
8-hydroxydeoxy-guanosine (OH^8dG)	Chen et al., 2007 [28]	Taiwan	HPLC with electrochemical detection	16 HD patients (M:F 9:7, 48.4±14.1 years) and 36 HC (M:F 19:17, 53.0±9.2 years)	Significant increase in HD patients
Deleted and total mitochondrial DNA (mtDNA) copy numbers	Chen et al., 2007 [28]	Taiwan	Real-time Quantitative PCR (RTQ-PCR)	16 HD patients (M:F 9:7, 48.4±14.1 years) and 36 HC (M:F 19:17, 53.0±9.2 years)	Significant increase in HD patients
Total mitochondrial DNA (mtDNA) copy numbers	Liu et al., 2008 [41]	Taiwan	Fluorescence-based Quantitative PCR	17 HD patients (M:F 8:5, 47.8±8.13 years) and 50 HC (M:F not given, 46.0±9.0 years)	Significant decrease in HD patients
mRNA expression levels of mtDNA-encoded mitochondrial enzymes	Chen et al., 2007 [28]	Taiwan	Real-time Quantitative PCR (RTQ-PCR)	16 HD patients (M:F 9:7, 48.4±14.1 years) and 36 HC (M:F 19:17, 53.0±9.2 years)	Non-significant differences between HD patients and HC
Expression levels of NADH dehydrogenase subunit 1 (ND1), cytochrome b (CYTB), and cytochrome c oxidase I (COXI)	Chen et al., 2007 [28]	Taiwan	Real-time Quantitative PCR (RTQ-PCR)	16 HD patients (M:F 9:7, 48.4±14.1 years) and 36 HC (M:F 19:17, 53.0±9.2 years)	Non-significant differences between HD patients and HC

PERIPHERAL BLOOD MONONUCLEAR CELLS

PARAMETER	AUTHOR, YEAR [REF]	COUNTRY	METHOD	STUDY SUBJECTS	MAIN FINDINGS
Aconitase 2	Chen et al., 2017 [42]	Taiwan	Spectrophotometry	19 HD patients (M:F 13:6, 48.7 ± 2.6 years), 6 HD gene asymptomatic carriers (M:F 4:2, 46.5 ± 7.8.6years), and 25 HC (M:F 15:10, 48.1. ± 2.4 years).	Significant decrease in HD patients and asymptomatic carriers. Correlation with motor score, independence scale, and functional capacity of the UHDRS and disease duration.

ERYTHROCYTES

PARAMETER	AUTHOR, YEAR [REF]	COUNTRY	METHOD	STUDY SUBJECTS	MAIN FINDINGS
Lipid peroxides (MDA + 4-hydroxyalkenals)	Túnez et al., 2011 [33]	Spain	Colorimetric chromogenic assay	19 HD patients (M:F 9:10, 43.50 ± 10.62 years) and 19 HC (M:F 8:11, 42.23 ± 9.34 21-58 years)	Significant increase in HD patients

Protein carbonyl	Túnez et al., 2011 [33]	Spain	HPLC gel filtration	19 HD patients (M:F 9:10, 43.50 ± 10.62 years) and 19 HC (M:F 8:11, 42.23 ± 9.34 21-58 years)	Significant increase in HD patients
Cu/Zn-Total SOD activity	Chen et al., 2007 [28]	Taiwan	Spectrophotometry with the use of RANSOD kit	16 HD patients (M:F 9:7, 48.4±14.1 years) and 36 HC (M:F 19:17, 53.0±9.2 years)	Significant decrease in HD patients
GPx activity	Chen et al., 2007 [28]	Taiwan	Spectrophotometry	16 HD patients (M:F 9:7, 48.4±14.1 years) and 36 HC (M:F 19:17, 53.0±9.2 years)	Significant decrease in HD patients
Glutathione reductase (GR) activity	Zanella et al., 1980 [43]	Italy	Microspectrofluoro-metry	10 HD (M:F 5:5, 30-80 years) and 10 HC (age- and sex-matched, data not given)	Significant increase in HD patients
Total glutathione	Zanella et al., 1980 [43]	Italy	Microspectrofluoro-metry	10 HD (M:F 5:5, 30-80 years) and 10 HC (age- and sex-matched, data not given)	Significant decrease in HD patients
Total glutathione	Túnez et al., 2011 [33]	Spain	Enzymatic assay	19 HD patients (M:F 9:10, 43.50 ± 10.62 years) and 19 HC (M:F 8:11, 42.23 ± 9.34 21-58 years)	Non-significant differences between HD patients and HC.
GSH	Túnez et al., 2011 [33]	Spain	Enzymatic assay	19 HD patients (M:F 9:10, 43.50 ± 10.62 years) and 19 HC (M:F 8:11, 42.23 ± 9.34 21-58 years)	Significant decrease in HD patients
GSSG	Túnez et al., 2011 [33]	Spain	Enzymatic assay	19 HD patients (M:F 9:10, 43.50 ± 10.62 years) and 19 HC (M:F 8:11, 42.23 ± 9.34 21-58 years)	Non-significant differences between HD patients and HC.
CAT	Zanella et al., 1980 [43]	Italy	Microspectrofluoro-metry	10 HD (M:F 5:5, 30-80 years) and 10 HC (age- and sex-matched, data not given)	Significant decrease in HD patients
LDH	Zanella et al., 1980 [43]	Italy	Microspectrofluoro-metry	10 HD (M:F 5:5, 30-80 years) and 10 HC (age- and sex-matched, data not given)	Non-significant differences between HD patients and HC
GADPH	Zanella et al., 1980 [43]	Italy	Microspectrofluoro-metry	10 HD (M:F 5:5, 30-80 years) and 10 HC (age- and sex-matched, data not given)	Non-significant differences between HD patients and HC
ATPase	Zanella et al., 1980 [43]	Italy	Microspectrofluoro-metry	10 HD (M:F 5:5, 30-80 years) and 10 HC (age- and sex-matched, data not given)	Non-significant differences between HD patients and HC
Hexokinase	Zanella et al., 1980 [43]	Italy	Microspectrofluoro-metry	10 HD (M:F 5:5, 30-80 years) and 10 HC (age- and sex-matched, data not given)	Significant increase in HD patients
Pyruvate-kinase	Zanella et al., 1980 [43]	Italy	Microspectrofluoro-metry	10 HD (M:F 5:5, 30-80 years) and 10 HC (age- and sex-matched, data not given)	Significant increase in HD patients
Thioredoxin-1 (Trx-1)	Sánchez-López et al., 2012 [36]	Spain	ELISA	13 HD patients (M:F 6:8, 42.0±10.65 years) and 29 HC (M:F 4:6, 39.0±12.51 years)	Decreased in HD patients
Thioredoxin-reductase 1 (TrRD-1) activity	Sánchez-López et al., 2012 [36]	Spain	ELISA	13 HD patients (M:F 6:8, 42.0±10.65 years) and 29 HC (M:F 4:6, 39.0±12.51 years)	Decreased in HD patients

SKIN FIBROBLASTS

PARAMETER	AUTHOR, YEAR [REF]	COUNTRY	METHOD	STUDY SUBJECTS	MAIN FINDINGS
Cytosolic ROS	Jędrak et al., 2018 [44]	Poland	ROS-sensitive fluorescent probe	8 HD patients (M:F 6:2, 41-65 years) and 7 HC (M:F 4:3, 41-65 years)	Non-significant differences between HD patients and HC
mtO2•-	Jędrak et al., 2018 [44]	Poland	ROS-sensitive fluorescent probe	8 HD patients (M:F 6:2, 41-65 years) and 7 HC (M:F 4:3, 41-	Non-significant differences between HD patients and HC

				65 years)	
Cu/Zn-SOD	Del Hoyo et al., 2006 [45]	Spain	Ultraviolet spectrophotometry	13 HD patients (M:F 8:5, 47.8±8.13 years) and 13 HC (M:F 6:7, 49.5±11.8 years)	Non-significant differences between HD patients and HC
Cu/Zn-SOD	Jędrak et al., 2018 [44]	Poland	Fluorescence antibodies labeling ¿Immunofluorescence?	8 HD patients (M:F 6:2, 41-65 years) and 7 HC (M:F 4:3, 41-65 years)	Non-significant differences between HD patients and HC
Mn-SOD	Del Hoyo et al., 2006 [45]	Spain	Ultraviolet spectrophotometry	13 HD patients (M:F 8:5, 47.8±8.13 years) and 13 HC (M:F 6:7, 49.5±11.8 years)	Non-significant differences between HD patients and HC
Mn-SOD	Jędrak et al., 2018 [44]	Poland	Immunofluorescence	8 HD patients (M:F 6:2, 41-65 years) and 7 HC (M:F 4:3, 41-65 years)	Significant increase in HD patients
GPx	Del Hoyo et al., 2006 [45]	Spain	Ultraviolet spectrophotometry	13 HD patients (M:F 8:5, 47.8±8.13 years) and 13 HC (M:F 6:7, 49.5±11.8 years)	Non-significant differences between HD patients and HC
GPx	Jędrak et al., 2018 [44]	Poland	Immunofluorescence	8 HD patients (M:F 6:2, 41-65 years) and 7 HC (M:F 4:3, 41-65 years)	Non-significant differences between HD patients and HC
CAT	Del Hoyo et al., 2006 [45]	Spain	Ultraviolet spectrophotometry	13 HD patients (M:F 8:5, 47.8±8.13 years) and 13 HC (M:F 6:7, 49.5±11.8 years)	Significant decrease in HD patients
CAT	Jędrak et al., 2018 [44]	Poland	Immunofluorescence	8 HD patients (M:F 6:2, 41-65 years) and 7 HC (M:F 4:3, 41-65 years)	Non-significant differences between HD patients and HC
GR	Jędrak et al., 2018 [44]	Poland	Immunofluorescence	8 HD patients (M:F 6:2, 41-65 years) and 7 HC (M:F 4:3, 41-65 years)	Significant increase in HD patients
Mitochondrial complexes I-V	Del Hoyo et al., 2006 [45]	Spain	Spectrophotometry	13 HD patients (M:F 8:5, 47.8±8.13 years) and 13 HC (M:F 6:7, 49.5±11.8 years)	Non-significant differences between HD patients and HC (both correcting for proteins and citrate-synthase)
Mitochondrial complexes I-V	Jędrak et al., 2018 [44]	Poland	Immunofluorescence	8 HD patients (M:F 6:2, 41-65 years) and 7 HC (M:F 4:3, 41-65 years)	Non-significant differences between HD patients and HC
Mitochondrial membrane potential	Jędrak et al., 2018 [44]	Poland	Immunofluorescence	8 HD patients (M:F 6:2, 41-65 years) and 7 HC (M:F 4:3, 41-65 years)	Non-significant differences between HD patients and HC
ATP	Jędrak et al., 2018 [44]	Poland	Luminescent Cell Viability Assay USA), based on the luciferase reaction	8 HD patients (M:F 6:2, 41-65 years) and 7 HC (M:F 4:3, 41-65 years)	Decreased in HD patients
CoQ_{10}	Del Hoyo et al., 2006 [45]	Spain	HPLC with electrochemical detection	13 HD patients (M:F 7:10, 46.0±13.0 years) and 13 HC (M:F 6:7, 49.5±11.8 years)	Non-significant differences between HD patients and HC
MAO-A mRNA and activity	Ooi et al., 2015 [46]	Singapore and Canada	Luciferase assay and luminescence	8 HD patients (M:F 3:5, 59.0±16.0 years) and 8 HC (M:F 2:6, 46.0±23.6 years)	Significant increase in HD neural cells

CEREBROSPINAL FLUID

PARAMETER	AUTHOR, YEAR [REF]	COUNTRY	METHOD	STUDY SUBJECTS	MAIN FINDINGS
Nitrates+ nitrites	Milstien et al., 1994 [49]	Canada and the United States of America	Colorimetric assay	33 HD patients and 16 HC (age and sex not given)	Non-significant differences between HD patients and HC

Quinolinic acid	Milstien et al., 1994 [49]	Canada and the United States of America	Colorimetric assay	33 HD patients and 16 HC (age and sex not given)	Non-significant differences between HD patients and HC

URINE

PARAMETER	AUTHOR, YEAR [REF]	COUNTRY	METHOD	STUDY SUBJECTS	MAIN FINDINGS
MDA/TBA	Olsson et al., 2021 [30]	Sweden and the United Kingdom	Spectrophotometry	118 HD patients (M:F 48:70, 22-79 years, 34 with premanifest HD) and 85 HC (M:F 36:49, 21-74 years)	Non-significant differences between HD patients and HC
Carbonyl proteins	Olsson et al., 2021 [30]	Sweden and the United Kingdom	Spectrophotometry	118 HD patients (M:F 48:70, 22-79 years, 34 with premanifest HD) and 85 HC (M:F 36:49, 21-74 years)	Non-significant differences between HD patients and HC
Oxyhemoglobin	Olsson et al., 2021 [30]	Sweden and the United Kingdom	ELISA	118 HD patients (M:F 48:70, 22-79 years, 34 with premanifest HD) and 85 HC (M:F 36:49, 21-74 years)	Significant increase in HD patients. Correlation with HD severity
Alpha-1-microglobulin	Olsson et al., 2021 [30]	Sweden and the United Kingdom	Radioimmuno-analyisis	118 HD patients (M:F 48:70, 22-79 years, 34 with premanifest HD) and 85 HC (M:F 36:49, 21-74 years)	Significant increase in HD patients

SALIVA

PARAMETER	AUTHOR, YEAR [REF]	COUNTRY	METHOD	STUDY SUBJECTS	MAIN FINDINGS
Uric acid	Corey-Bloom et al., 2020 [25]	United States of America	Colorimetric enzymatic assay	94 HD patients (M:F 39:55, 50.4 $\pm$ 13.8 years, 49 presymptomatic) and 84 HC (M:F 43:41,52.9 $\pm$ 16.0 years)	Significantly lower in female pre-HD and manifest HD patients, and male manifest HD, compared to HC

Supplementary Table S4. Oxidative stress markers in experimental models of Huntington's disease (HD). ATP adenosine-triphosphate, CAT catalase; CPD chlorpyrifos; DNA deoxyribonucleic acid; GADPH Gliceraldehyde-phosphate-dehydrogenase; GPCPD1 Glycerophosphocholine phosphodiesterase 1; G6PD Glucose-6-phosphate dehydrogenase; GPx glutathione peroxidase; GR glutathione reductase; GSH reduced glutathione; GSK-3β Glycogen synthase kinase-3β; GSSG oxidized glutathione; GST glutathione-S-transferase; HD Huntington's disease; 4-HNE 4-hydroxynonenal, HPLC high performance liquid chromatograph; IRP iron response protein; MDA malonyldialdehyde; MAO-A monoamine oxidase A; MTT 4,5-dimethyl-thiazol-2-yl)-2,5-diphenyl-tetrazolium bromide; NADH nicotin-adenin-dinucleotide; NADPH nicotin-adenin-dinucleotide-phosphate; NOS nitric oxide synthase; NOX NADPH oxidase; 3-NPA 3-nitropropionic acid; OH^8dG 8-hydroxy-deoxyguanosine; PBMC peripheral blood mononuclear cells; PDXK Pyridoxal kinase; 6PGD 6-phosphogluconate dihydrogen-ase; PLP pyridoxal 5′-phosphate; Prx peroxiredoxin; QCCR cytochrome c oxidoreductase; ROS reactive oxygen species; RT-PCR real time-quantitative polymerase chain reaction; SDH Succinate dehydrogenase; SOD superoxide dismutase; STH Striatal cells expressing wild-type Htt; TBARS thiobarbituric acid reactive substances; TAR total antioxidant reactivity; TRAP Total radical-trapping antioxidant potential; TRR thioredoxin reductase; WT wild-type.

LIPID PEROXIDATION MARKERS						
**PARAMETER**	**AUTHOR, YEAR [REF]**	**COUNTRY**	**EXPERIMENTAL MODEL**	**TISSUE**	**METHOD**	**MAIN FINDINGS**
Lipid peroxides (MDA + 4-hydroxyalkenals)	Túnez et al., 2004 [58]	Spain	3-month-old male Wistar rats. Intraperitoneal administration of 3-nitro propionic acid (3-NPA)	Striatal and cortical synaptosomes	Spectrophotometry	Significant increase compared to vehicle controls
MDA	Yang et al., 2005 [59]	United States of America	3-month-old male C57 black mice. Intraperitoneal administration of 3-NPA	Brain homogenates from the striatum, cortex, and cerebellum	HPLC	Significant increase compared to vehicle controls
Lipid peroxides (MDA + 4-hydroxyalkenals)	Túnez et al., 2006 [60]	Spain and Mexico	3-month-old male Wistar rats. Intraperitoneal administration of 3-NPA	Whole brain homogenates	Spectrophotometry	Significant increase compared to vehicle controls
MDA	Kumar et al., 2006 [61]	India	Male Wistar rats weighing 180-250 g. Intraperitoneal administration of 3-NPA	Whole brain homogenates	Spectrophotometry	Significant increase compared to vehicle controls
MDA	Kumar & Kumar 2009 [62]	India	Male Wistar rats weighing 250-300 g. Intraperitoneal administration of 3-NPA	Brain homogenates from the striatum, cortex, and hippocampus	Spectrophotometry	Significant increase compared to vehicle controls
MDA	Sandhir et al., 2010 [63]	India	Female Wistar rats weighing 200-250 g. Intraperitoneal administration of 3-NPA	Brain homogenates from the striatum.	Spectrophotometry	Significant increase compared to vehicle controls
Lipid peroxides (MDA + 4-hydroxyalkenals)	Tasset et al., 2011 [64]	Spain	3-month-old male Wistar rats weighing 220-250 g. Intraperitoneal administration of 3-	Striatum and the rest of the brain homogenates	Spectrophotometry	Significant increase compared to vehicle controls

			NPA			
MDA	Gopinath et al., 2011 [65]	India	Male Wistar rats weighing 250-300 g. Intraperitoneal administration of 3-NPA	Brain homogenates from the striatum and plasma	Spectrophotometry	Significant increase compared to vehicle controls
MDA	Bhateha et al., 2012 [66]	India	Male Wistar rats weighing 180-220 g. Intraperitoneal administration of 3-NPA	Whole brain homogenates	Spectrophotometry	Significant increase compared to vehicle controls
MDA	Shivasharan et al., 2012 [67]	India	Adult female Wistar rats weighing 190-200 g. Intraperitoneal administration of 3-NPA	Whole brain homogenates	Spectrophotometry	Significant increase compared to vehicle controls
MDA	Denny-Joseph & Muralidhara, 2013 [68]	India	4-week-old male Wistar rats. Intraperitoneal administration of 3-NPA	Brain homogenates from the striatum and cerebellum	Fluorescence. Spectrophotometry	Non.-significant differences compared to vehicle controls
Hydroper-oxides	Denny-Joseph & Muralidhara, 2013 [68]	India	4-week-old male Wistar rats. Intraperitoneal administration of 3-NPA	Brain homogenates from the striatum and cerebellum	Fluorescence. Spectrophotometry	Significant increase compared to vehicle controls
MDA	Binawade & Jagtap, 2013 [69]	India	Female Sprague-Dawley rats weighing 200-250. Intraperitoneal administration of 3-NPA	Whole brain homogenates	Spectrophotometry	Significant increase compared to vehicle controls
Lipid peroxides	Sandhir et al., 2014 [70]	India	Female Wistar rats weighing 180-200 g. Intraperitoneal administration of 3-NPA	Brain homogenates from the striatum.	Spectrophotometry	Significant increase compared to vehicle controls
MDA	Thangarajan et al., 2014 [71] and 2016 [72]	India	Male Wistar rats weighing 200-250 or male albino rats weighing 250-300. Intraperitoneal administration of 3-NPA	Brain homogenates from the striatum	Spectrophotometry	Significant increase compared to vehicle controls
MDA-TBARS	Gupta & Sharma, 2014 [73]	India	Adult albino Wistar rats 3-5 months old weighing 200-250 g. Intraperitoneal administration of 3-NPA	Brain homogenates from the striatum	Spectrophotometry	Significant increase compared to vehicle controls
MDA-TBARS	Hariharan et al., 2014 [74]	India	Adult female Wistar rats weighing 200-250 g. Intraperitoneal administration of 3-NPA	Whole brain homogenates (except cerebellum)	Spectrophotometry	Significant increase compared to vehicle controls

MDA	Khan et al., 2015 [75]	India	Adult female Wistar rats weighing 250-300 g. Intraperitoneal administration of 3-NPA	Brain homogenates from the striatum	Spectrophotometry	Significant increase compared to vehicle controls
TBARS	Courtes et al., 2015 [76]	Brazil	Adult female Wistar rats weighing 200-250 g. Intraperitoneal administration of 3-NPA	Brain homogenates from the striatum and cortex	Spectrophotometry	Significant increase compared to vehicle controls
MDA-TBARS	Silva-Palacios et al., 2017 [77]	Mexico	Adult (9 months-old) and old (24 months-old) albino Wistar female Rats. Intraperitoneal administration of 3-NPA	Brain homogenates from the striatum	Spectrophotometry	Significant increase compared to vehicle controls only in the old-group
MDA	Badini et al., 2024 [78]	Iran	Male Wistar rats weighing 220-250 g. Intraperitoneal administration of 3-NPA	Whole brain homogenates	Colorimetric method	Significant decrease compared to vehicle controls
Lipid peroxides (MDA + 4-hydroxyalkenals)	Tasset et al., 2011 [64]	Spain	Murine neuroblastoma N1E-115 cells incubated with 3-NPA	Murine neuroblastoma N1E-115 cells incubated with 3-NPA	Spectrophotometry	Significant increase compared to vehicle controls
4-hydroxynonenal (4-HNE)	Ryu et al., 2006 [79]	Canada	Male Sprague–Dawley rats weighting 280–300 g. Intrastriatal injection of quinolinic acid	Striatum slices	Immuno-histoche-mistry	Significant increase compared to vehicle controls
MDA	Kalonia et al., 2009 [80]	India	Male Wistar rats weighing 220-250 g. Intrastriatal injection with quinolinic acid	Brain homogenates from the cortex and striatum	Spectrophotometry	Significant increase compared to vehicle controls
Lipid peroxides and MDA	Maldonado et al., 2010 [81]	Mexico	Male Wistar weighing 270–320 g. Intrastriatal injection of quinolinic acid	Striatal homogenates	Fluores-cence spectro-metry	Significant increase compared to vehicle controls
MDA	Kalonia et al., 2010 [82]	India	Male Wistar rats weighing 250-300 g. Intrastriatal injection with malonic acid	Brain homogenates from the striatum	Spectrophotometry	Significant increase compared to vehicle controls
MDA	Sumathi et al., 2018 [83]	India	Male albino rats weighing 250-300. Intrastriatal injection with quinolinic acid	Brain homogenates from the striatum	Spectrophotometry	Significant increase compared to vehicle controls

Lipid peroxides	Purushothaman & Sumathi, 2022 [84]	India	Male albino rats weighing 250-300. Intrastriatal injection with quinolinic acid	Brain homogenates from the striatum	Spectrophotometry	Significant increase compared to vehicle controls
Lipid peroxides	Pérez-de la Cruz, 2005 [85]	Mexico and the United States of America	Male Wistar weighing 270–300 g	Whole brain homogenates. Brain synaptic vesicles exposed to quinolinic acid.	Spectrophotometry	Significant increase compared to vehicle controls
TBARS	Leipnitz et al. [86]	Brazil	30-days Wistar rats	Homogenates from the cerebral cortex incubated with quinolinic acid	Spectrophotometry	Significant increase compared to vehicle controls
Lipid peroxides	Pérez-de la Cruz, 2005 [87]	Mexico and the United States of America	Male Wistar weighing 270–300 g	Whole brain homogenates. Brain synaptic vesicles exposed to quinolinic acid.	Spectrophotometry	Significant increase compared to vehicle controls
MDA	Colle et al., 2012 [88]	Brazil	Male Wistar rats weighing 180-220 g.	Striatal slices incubated with 3-NPA, quinolinic acid, or both	Spectrophotometry	Significant increase compared to vehicle controls
Lipid peroxides	Pérez-Severiano et al. 2000 [89]	Mexico	R6/1 mice (transgenic for the HD mutation)	Striatum	Fluorometric method	Significant increase compared to wild-type mice
4-hydroxynonenal (4-HNE)	Lee et al., 2011 [7]	United States of America	Male transgenic HD (R6/2 strain), CAG140 and N171-82Q mice	Striatum	Immunohistochemistry	Significant increase compared to wild-type mice
MDA	Johri et al., 2012 [90]	United States of America	Transgenic R6/2 mice and their wild-type littermates	Brain homogenates from the striatum	HPLC	Significant increase compared to wild-type
MDA	Chang et al., 2024 [24]	Taiwan	Heterozygous R6/2 transgenic mice and female control mice (B6CBAFI/J)	Brain homogenates from the striatum and cortex	Immunohistochemistry	Significant increase compared to wild-type
MDA	Chandra et al., 2016 [91]	United States of America	BACHD founder mice expressing expanded human Htt with 97 mixed CAA–CAG repeats	Striatum	HPLC with fluorescence detection	Significant increase compared to wild-type mice
TBARS	Brocardo et al., 2016 [92]	Canada	Yeast artificial chromosome 128 (YAC 128) line of transgenic mice	Homogenates from cerebellum, cerebral cortex, prefrontal cortex, hippocampus, and striatum	Spectrophotometry	Similar concentrations than in wild-type mice
MDA	Askeland et al., 2018 [93]	Norway and the Czech Republic	Transgenic minipigs (Sus scrofa domesticus, Linnaeus) with the N-terminal part of human mutated huntingtin and their WT siblings	Frontal cortex, basal ganglia, and PBMC	Fluorometric method	Non-significant differences compared to wild-type mice

MDA	Dominah et al., 2017 [94]	United States of America	*STHdhQ7/Q7 and STHdhQ111/Q111* striatal cell lines. Incubation with chlorpyrifos (CPF)	Cellular cultures	Fluoro-metric method	Significant increase compared to wild-type
**PROTEIN OXIDATION MARKERS**						
**PARAMETER**	**AUTHOR, YEAR [REF]**	**COUNTRY**	**EXPERIMENTAL MODEL**	**TISSUE**	**METHOD**	**MAIN FINDINGS**
Protein carbonyls	La Fontaine et al, 2000 [95, 96]	United States of America	4-month-old Sprague-Dawley rats. Intraperitoneal administration of 3-nitro propionic acid (3-NPA)	Striatal and cortical synaptosomes	Immuno-chemical detection	Significant increase compared to vehicle controls
Protein carbonyls	Túnez et al., 2004 [48]	Spain	3-month-old male Wistar rats). Intraperitoneal administration of 3-NPA	Striatal and cortical synaptosomes	Spectrophotometry	Significant increase compared to vehicle controls
Protein carbonyls	Túnez et al., 2006 [60]	Spain and Mexico	3-month-old male Wistar rats). Intraperitoneal administration of 3-NPA	Whole brain homogenates	Spectrophotometry	Significant increase compared to vehicle controls
Protein carbonyls	Maldonado et al., 2010 [81]	Mexico	Male Wistar weighing 270–320 g. Intrastriatal injection of quinolinic acid	Striatal homogenates	Fluorescence spectrometry	Significant increase compared to vehicle controls
Protein carbonyls	Gopinath et al., 2011 [65]	India	Male Wistar rats weighing 250-300 g. Intraperitoneal administration of 3-NPA	Brain homogenates from the striatum and plasma	Spectrophotometry	Significant increase compared to vehicle controls
Protein carbonyls	Bhateha et al., 2012 [66]	India	Male Wistar rats weighing 180-220 g. Intraperitoneal administration of 3-NPA	Whole brain homogenates	Spectrophotometry	Significant increase compared to vehicle controls
Protein carbonyls	Denny-Joseph & Muralidhara, 2013 [68]	India	4-week-old male Wistar Intraperitoneal administration of 3-NPA	Brain homogenates from the striatum and cerebellum	Fluores-cence s spectrometry	Significant increase compared to vehicle controls
Protein carbonyls	Sandhir et al., 2014 [70]	India	Female Wistar rats weighing 180-200 g. Intraperitoneal administration of 3-NPA	Brain homogenates from the striatum	Spectrophotometry	Significant increase compared to vehicle controls
Protein carbonyls	Souza et al., 2014 [97]	Brazil	Male Wistar rats weighing 200-250 g. Intraperitoneal administration of 3-NPA	Brain homogenates from the striatum	Spectrophotometry	Non-significant increase compared to vehicle controls
Protein carbonyls	Thangarajan et al., 2014 [71] and 2016 [72]	India	Male albino rats weighing 250-300. Intraperitoneal administration of 3-	Brain homogenates from the striatum	Spectrophotometry	Significant increase compared to vehicle controls

NPA						
Protein carbonyls	Silva-Palacios et al., 2017 [77]	Mexico	Adult (9 months-old) and old (24 months-old) albino Wistar female Rats. Intraperitoneal administration of 3-NPA	Brain homogenates from the striatum	Immuno-blotting	Significant increase compared to vehicle controls only in the old-group
Protein carbonyls	Colin-González et al., 2013 [98]	Mexico	Male Wistar rats weighing 270–310 g. Intrastriatal injection with quinolinic acid	Brain homogenates from the striatum	Spectrophotometry	Significant increase compared to vehicle controls
Protein carbonyls	Antunes-Wilhelm et al, 2013 [99]	Brazil	Male Wistar rats weighing 200–250 g. Intrastriatal injection with quinolinic acid	Brain homogenates from the striatum	Spectrophotometry	Significant increase compared to vehicle controls
Protein carbonyls	Sumathi et al., 2018 [83]	India	Male albino rats weighing 250-300. Intrastriatal injection with quinolinic acid	Brain homogenates from the striatum	Spectrophotometry	Significant increase compared to vehicle controls
Protein carbonyls	Purushothaman & Sumathi, 2022 [84]	India	Male albino rats weighing 250-300. Intrastriatal injection with quinolinic acid	Brain homogenates from the striatum	Spectrophotometry	Significant increase compared to vehicle controls
Protein carbonyls	Sorolla et al., 2008 [12]	Spain	20-week-old transgenic Tet/HD94 mice and wilt-type littermates	Brain homogenates from the cortex, striatum, and cerebellum	Spectrophotometry	Significant increase compared to wild-type mice
Protein carbonyls	Brocardo et al., 2016 [92]	Canada	Yeast artificial chromosome 128 (YAC 128) line of transgenic mice	Homogenates from cerebellum, cerebral cortex, prefrontal cortex, hippocampus, and striatum	Spectrophotometry	Similar concentrations than in wild-type mice
Protein carbonyls	Lou et al., 2016 [100]	United States of America	9 week-old R6/2 mice (transgenic for the HD mutation)	Brain homogenates from the cortex and striatum	Immuno-chemical detection	Significant increase in the cortex compared to wild-type mice
Protein carbonyls	Pinho et al., 2020 [101]	Portugal and the United Kingdom	11-week male transgenic R6/2 mice (B6CBA-Tg(HDexon1)62Gpb/3J) , and male wild-type (WT) B6CBAF1/J mice	Whole brain homogenates, liver and muscle homogenates	Mass spectro-metry	Significant increase in liver and muscle, but not in brain, compared to wild-type mice
**DNA OXIDATION MARKERS**						
**PARAMETER**	**AUTHOR, YEAR [REF]**	**COUNTRY**	**EXPERIMENTAL MODEL**	**TISSUE**	**METHOD**	**MAIN FINDINGS**
OH^8Dg	Acevedo-Torres et al., 2009 [102]	Puerto Rico (USA)	5-26 weeks-old male C57BL/6 mice. Intraperitoneal injection of 3-NPA	Slices from the striatum, cerebral cortex and	Immuno-cytochemistry	Significant increase compared to vehicle controls

				cerebellum		
OH8Dg	Kim et al., 2002 [103]	United States of America and South Korea	Heterozygotes Sod2 −/+ mice and the Wt littermates. Intraperitonal injection of 3-NPA	Striatum	Immuno-histoche-mistry	Increased in the Sod2 −/+ mice compared with the wild-type mice
OH8Dg	Ryu et al., 2006 [79]	Canada	Male Sprague–Dawley rats weighting 280–300 g. Intrastriatal injection of quinolinic acid.	Striatum	Immuno-histoche-mistry	Significant increase compared to vehicle controls
OH8Dg	Bogdanov et al., 2001 [104]	United States of America	Male transgenic HD (R6/2 strain) mice and female B6CBAF1/J mice	Microdialysis from the striatum, nuclear DNA from brain tissue, plasma, and urine	HPLC with electroche mical detection. Immuno-histoche-mistry	Significant increase compared to wild-type mice
OH8Dg	Acevedo-Torres et al., 2009 [102]	Puerto Rico (USA)	Male transgenic HD (R6/2 strain) and littermate controls (7, 10 and 12 week-old)	Slices from the striatum and cerebral cortex	Immuno-cytochemis try	Significant increase compared to wild-type mice
OH8Dg	Chandra et al..2016 [91]	United States of America	BACHD founder mice expressing expanded human Htt with 97 mixed CAA–CAG repeats	Striatum	HPLC with electroche mical detection	Significant increase compared to wild-type mice
8-oxo guanine (8-oxoG)	Acevedo-Torres et al., 2009 [102]	Puerto Rico (USA)	5-26 weeks-old male C57BL/6. Intraperitoneal injection of 3-NPA	Slices from the striatum, cerebral cortex and cerebellum	Immuno-cytochemis try	Significant increase compared to vehicle controls
8-oxoguanine (8-oxoG)	Acevedo-Torres et al., 2009 [102]	Puerto Rico (USA)	Male transgenic HD (R6/2 strain) and littermate controls (7, 10 and 12 week-old)	Slices from the striatum and cerebral cortex	Immuno-cytochemis try	Significant increase compared to wild-type mice
8-oxo guanine (8-oxoG)	Askeland et al., 2018 [93]	Norway and the Czech Republic	Transgenic minipigs (Sus scrofa domesticus, Linnaeus) with the N-terminal part of human mutated huntingtin and their WT siblings	Frontal cortex, basal ganglia, and PBMC	Fluorometr ic method	Significant increase compared to wild-type minipigs
**NITROSATIVE AND NITROXIDATIVE STRESS MARKERS**						
**PARAMETER**	**AUTHOR, YEAR [REF]**	**COUNTRY**	**EXPERIMENTAL MODEL**	**TISSUE**	**METHOD**	**MAIN FINDINGS**
Nitrites	Kumar et al., 2006 [61]	India	Male Wistar rats weighing 180-250 g. Intraperitoneal administration of 3-NPA	Whole brain homogenates	Colorimetr ic method and spectropho tometry	Significant increase compared to vehicle controls
Nitrites	Kumar & Kumar 2009 [62]	India	Male Wistar rats weighing 250-300 g. Intraperitoneal administration of 3-NPA	Brain homogenates from the striatum, cortex, and hippocampus	Colorimetr ic method and spectropho tometry	Significant increase compared to vehicle controls

Nitrites	Sandhir et al., 2010 [63]	India	Female Wistar rats weighing 200-250 g. Intraperitoneal administration of 3-NPA	Brain homogenates from the striatum.	Spectrophotometry	Significant increase compared to vehicle controls
NO production (nitrates + nitrites)	Chang et al, 2011 [105]	Singapore	16-week-old male Sprague-Dawley rats weighing 290-350 g. Intraperitoneal administration of 3-NPA	Brain homogenates from striatum, cortex and hippocampus, and plasma	Colorimetric method and spectrophotometry	Significant decrease in brain homogenates compared to vehicle controls. Non-significant differences in plasma
Nitrites	Bhateha et al., 2012 [66]	India	Male Wistar rats weighing 180-220 g. Intraperitoneal administration of 3-NPA	Whole brain homogenates	Colorimetric method and spectrophotometry	Significant increase compared to vehicle controls
Nitrites	Shivasharan et al., 2012 [67]	India	Adult female Wistar rats weighing 190-200 g. Intraperitoneal administration of 3-NPA	Whole brain homogenates	Spectrophotometry	Significant increase compared to vehicle controls
Nitrites	Denny-Joseph & Muralidhara, 2013 [68]	India	4-week-old male Wistar Intraperitoneal administration of 3-NPA	Brain homogenates from the striatum and cerebellum	Fluores-cence. Spectrophotometry	Significant increase compared to vehicle controls
Nitrites	Binawade & Jagtap, 2013 [69]	India	Female Sprague-Dawley rats weighing 200-250. Intraperitoneal administration of 3-NPA	Whole brain homogenates	Spectrophotometry	Significant increase compared to vehicle controls
Nitrites	Thangarajan et al., 2014 [71] and 2016 [72]	India	Male albino rats weighing 250-300. Intraperitoneal administration of 3-NPA	Brain homogenates from the striatum	Spectrophotometry	Significant increase compared to vehicle controls
Nitrites	Gupta & Sharma, 2014 [73]	India	Adult albino Wistar rats 3-5 months old weighing 200-250 g. Intraperitoneal administration of 3-NPA	Brain homogenates from the striatum	Spectrophotometry	Significant increase compared to vehicle controls
Nitrites	Hariharan et al., 2014 [74]	India	Adult female Wistar rats weighing 200-250 g. Intraperitoneal administration of 3-NPA	Whole brain homogenates (except cerebellum)	Colorimetric method and spectrophotometry	Significant increase compared to vehicle controls
Nitrites	Khan et al., 2015 [75]	India	Adult female Wistar rats weighing 250-300 g. Intraperitoneal administration of 3-NPA	Brain homogenates from the striatum	Colorimetric method and spectrophotometry	Significant increase compared to vehicle controls
Nitrites	Tasset et al., 2011 [64]	Spain	Murine neuroblastoma N1E-115 cells incubated with 3-NPA	Murine neuroblastoma N1E-115 cells	Colorimetric method	Significant increase compared to vehicle controls

				incubated with 3-NPA		
Nitrites	Kalonia et al., 2009 [80]	India	Male Wistar rats weighing 220-250 g. Intrastriatal injection with quinolinic acid	Brain homogenates from the cortex and striatum	Spectrophotometry	Significant increase compared to vehicle controls
Nitrites	Kalonia et al., 2010 [82]	India	Male Wistar rats weighing 250-300 g. Intrastriatal injection with malonic acid	Brain homogenates from the striatum	Spectrophotometry	Significant increase compared to vehicle controls
Nitrites	Sumathi et al., 2018 [83]	India	Male albino rats weighing 250-300. Intrastriatal injection with quinolinic acid	Brain homogenates from the striatum	Spectrophotometry	Significant increase compared to vehicle controls
Nitrites	Purushothaman & Sumathi, 2022 [84]	India	Male albino rats weighing 250-300. Intrastriatal injection with quinolinic acid	Brain homogenates from the striatum	Spectrophotometry	Significant increase compared to vehicle controls
NO production (nitrates + nitrites)	L'Episcopo et al., 2016 [23]	Italy, Sweden, Canada, and the United Kingdom	2.5–3 weeks and 48 weeks-old R6/2 transgenic mice and wild.type littermates.	Primary neuronal and primary astrocytic cultures	Colorimetric method	Increased NO production compared to wild-type mice
Peroxynitrite	Pérez-de la Cruz et al., 2005 [87]	Mexico and the United States of America	Male Wistar weighing 270–300 g. Intrastriatal injection of quinolinic acid.	Brain homogenates from the striatum	HPLC with electrochemical detection	Significant increase compared to vehicle controls
Nitrotyrosine	Kim et al., 2002 [103]	United States of America and South Korea	Heterozygotes Sod2 −/+ mice and the Wt littermates. Intraperitonal injection of 3-NPA	Brain homogenates from the striatum	Immuno-histoche-mistry	Increased in the Sod2 −/+ mice compared with the wild-type mice a
NOS activity	Pérez-de la Cruz et al.,, 2005 [87]	Mexico and United States of America	Male Wistar weighing 270–300 g. Intrastriatal injection of quinolinic acid.	Brain homogenates from the striatum	Stoichiometric method	Significant increase compared to vehicle controls
NOS activity	Pérez-Severiano et al., 2002 [106]	Mexico	11-, 19- and 35-week-old R6/1 and nontransgenic littermates	Brain homogenates from the striatum	Stoichio-metric method	Significant increase compared to wild-type at 19 weeks, and decrease at 39 weeks, compared to wild-type
NOS mRNA and protein expression	Jang and Cho, 2016 [107]	South Korea	MaleC57BL/6 mice weighing 23-25 g). Intraperitoneal administration of 3-NPA	Striatal slices	Immuno-histochemistry	Significant increase of iNOS compared to vehicle controls
NOS mRNA and protein expression	Aguilera et al., 2007 [108]	Mexico	Male Wistar weighing 270–320 g. Intrastriatal injection of quinolinic acid.	Striatal sections	RT-PCR and immu-nohistochemistry	Significant transitory increase of eNOS, iNOS, and nNOS compared to vehicle controls
NOS mRNA and protein expression	Napolitano et al., [109]	Italy	2-3 months Wistar rats for striatal slices SK-N-MC human	Striatal slices or SK-N-MC cells incubated	Immuno-blotting and	Significant increase of iNOS and eNOS compared to vehicle

			neuroblastoma cells	with 3-NPA	chemiluminescence	controls
**GLOBAL OXIDATIVE STRESS MARKERS AND TRACE METALS**						
**PARAMETER**	**AUTHOR, YEAR [REF]**	**COUNTRY**	**EXPERIMENTAL MODEL**	**TISSUE**	**METHOD**	**MAIN FINDINGS**
ROS production	Sandhir et al., 2010 [63]	India	Female Wistar rats weighing 200-250 g. Intraperitoneal administration of 3-NPA	Brain homogenates from the striatum.	Spectrophotometry	Significant increase compared to vehicle controls
ROS production	Denny-Joseph & Muralidhara, 2013 [68]	India	4-week-old male Wistar Intraperitoneal administration of 3-NPA	Brain homogenates from the striatum and cerebellum	Fluores-cence. Spectrophotometry	Significant increase compared to vehicle controls
ROS production	Sandhir et al., 2014 [70]	India	Female Wistar rats weighing 180-200 g. Intraperitoneal administration of 3-NPA	Brain homogenates from the striatum.	Spectrophotometry	Significant increase compared to vehicle controls
ROS production	Souza et al., 2014 [97]	Brazil	Male Wistar rats weighing 200-250 g. Intraperitoneal administration of 3-NPA	Brain homogenates from the striatum	Spectrofluorometric method	Significant increase compared to vehicle controls
ROS production	Courtes et al., 2015 [76]	Brazil	Adult female Wistar rats weighing 200-250 g. Intraperitoneal administration of 3-NPA	Brain homogenates from the striatum and cortex	Fluorescence method	Significant increase compared to vehicle controls
ROS production	Ryu et al., 2006 [79]	Canada	Male Sprague–Dawley rats weighing 280–300 g. Intrastriatal injection of quinolinic acid.	Striatum	Fluorescence method	Significant increase compared to vehicle controls
ROS production	Antunes-Wilhelm et al, 2013 [99]	Brazil	Male Wistar rats weighing 200–250 g. Intrastriatal injection with quinolinic acid	Brain homogenates from the striatum	Fluores-cence-Spectrophotometry	Significant increase compared to vehicle controls
ROS production	Colle et al., 2012 [88]	Brazil	Male Wistar rats weighing 180-220 g.	Striatal slices incubated with 3-NPA, quinolinic acid, or both	Spectrophotometry	Significant increase compared to vehicle controls
ROS production	Peterson et al., 2022 [110]	Denmark	11 week male transgenic R6/2 mice, and young R6/2 males and young R62 mice backcrossed with healthy CBAxC57BL-6 hybrid females	Striatal and cortical synaptosomes	Fluores-cence method	Significant increase compared to wild-type mice in cortical, but a non-significant trend towards an increase in cortical synaptosomes
ROS production	Fernández et al., 2023 [111]	Spain	2-months and 7-months R6/1 transgenic HC mice and wild-type controls	Primary cultures of chromaffin cells from the adrenal medulla	Fluores-cence-Spectrophotometry	Significant increase compared to age-matched wild-type mice, and increase with age in both groups.

ROS production	Lim et al., 2008 [112]	Italy	E14 striatal primor dia of KI-HdhQ111 (mutant for HTT) and WT-HdhQ7 littermate mouse embryos	Cellular cultures	Fluores-cence-spectrophotometry	Significant increase compared to wild-type cells
ROS production	Ribeiro et al., 2012 [113]	Portugal	Striatal cells expressing wild-type Htt (STHdhQ7/Q7 or wild-type cells; clone 2aA5) or homozygous mutant cells derived from knock-in mice, expressing FL-mHtt with 111 glutamines (STHdhQ111/ Q111cells; clone 109-1A),	Cellular cultures.	Fluores-cence-spectrophotometry	Significant increase compared to wild-type cells
ROS production	Frederick et al., 2014 [114]	France, the United States of America, and Georgia	*STHdhQ7/Q7 and STHdhQ111/Q111* striatal cell lines, derived from neuronal precursor cells isolated from knock-in mice	Cellular cultures	Spectrophotometry	Significant increase compared to wild-type cells
ROS production	Dominah et al., 2017 [94]	United States of America	*STHdhQ7/Q7 and STHdhQ111/Q111* striatal cell lines. Incubation with chlorpyrifos (CPF)	Cellular cultures	Fluoro-metric method	Significant increase compared to wild-type
ROS production	Wang et al., 2013 [115]	China	YAC128 HD transgenic mice	Cellular cultures of YAC128 HD mice embryonic fibroblast (MEF) and primary medium spiny neurons (MSN)	Fluores-cence-spectrophotometry	Significant increase compared to wild-type cells
ROS production	Choi et al., 2013 [116]	South Korea	Immortalized striatal progenitor cell line (STHdhQ7), which expresses endogenous wild-type huntingtin	Cellular cultures. Incubation with 3-NPA	Fluores-cence-spectrophotometry	Significant increase compared to vehicle controls
ROS production	Pruccoli et al., 2021 [117]	Italy and the United Kingdom	Inducible rat pheochromocytoma (PC12) cells expressing the HTT gene with 23 or 74 glutamine repeats (HD-Q23 or HD-Q74)	Cellular cultures	Fluores-cence method with a multilabel plate reader	Significant increase in HD-Q74 but not in HD-Q23 cells
Total radical-trapping antioxidant potential (TRAP)	Túnez et al., 2006 [60]	Spain and Mexico	3-month-old male Wistar rats). Intraperitoneal administration of 3-nitropropionic acid	Whole brain homogenates	Chemiluminescence method	Significant decrease compared to vehicle controls

Total radical-trapping antioxidant potential (TRAP)	Leipnitz et al. [86]	Brazil	30-days Wistar rats	Homogenates from the cerebral cortex incubated with quinolinic acid	Chemiluminescence method	Significant decrease compared to vehicle controls
Total antioxidant reactivity (TAR)	Leipnitz et al. [86]	Brazil	30-days Wistar rats	Homogenates from the cerebral cortex incubated with quinolinic acid	Chemiluminescence method	Significant decrease compared to vehicle controls
Superoxide anion (O2*) production	Maldonado et al., 2010 [81]	Mexico	Male Wistar weighing 270–320 g. Intrastriatal injection of quinolinic acid	Striatal homogenates	Fluores-cence spectro-metry	Significant increase compared to vehicle controls
Superoxide anion (O2*) production	Kim et al., 2002 [103]	United States of America and South Korea	Heterozygotes Sod2 −/+ mice and the Wt littermates. Intraperitonal injection of 3-NPA	Striatum	Immuno-histoche-mistry	Increased in the Sod2 −/+ mice compared with the wild-type mice a
Iron	Fox et al., 2007 [118]	United States of America and Australia	12-week transgenic R6/2 mice	Brain homogenates from the cortex and striatum	Inductively - coupled plasma mass spectroscopy	Significant increase compared to wild-type mice
Iron	Chen et al., 2013 [119]	United States of America	Male transgenic R6/2 mice backcrossed with female B6/CBA mice, and N171-82Q HD mice bakcrossed with female B6/C3H mice	Brain sections from the cortex and striatum	Inductively - coupled plasma mass spectroscopy	Significant increase of Fe2+ compared to wild-type mice (initially in the cortex and later in the striatum)
Ferritin	Simmons et al., 2007 [19]	United States of America	Transgenic R6/2 mice	Brain homogenates from cortex, striatum, and hippocampus	Double immunofluorescence labeling	Significant increase in the microglia compared to age-matched wild-type mice
Transferrin	Chen et al., 2013 [119]	United States of America	Male transgenic R6/2 mice backcrossed with female B6/CBA mice, and N171-82Q HD mice bakcrossed with female B6/C3H mice	Brain sections from the cortex and striatum	Immunofluorescence	Non-significant changes compared to wild-type mice
Transferrin receptor	Chen et al., 2013 [119]	United States of America	Male transgenic R6/2 mice backcrossed with female B6/CBA mice, and N171-82Q HD mice bakcrossed with female B6/C3H mice	Brain sections from the cortex and striatum	Immunofluorescence	Significant decrease in striatum and cortex compared to wild-type mice
Iron response proteins 1 and 2 (IRP-1 and IRP-2)	Chen et al., 2013 [119]	United States of America	Male transgenic R6/2 mice backcrossed with female B6/CBA mice, and N171-82Q HD mice bakcrossed with female B6/C3H mice	Brain sections from the cortex and striatum	Immunofluorescence	Significant decrease of IRP-1 in striatum and cortex and of IRP-2 in striatum compared to wild-type mice

Ferroportin	Chen et al., 2013 [119]	United States of America	Male transgenic R6/2 mice backcrossed with female B6/CBA mice, and N171-82Q HD mice bakcrossed with female B6/C3H mice	Brain sections from the cortex and striatum	Immunofluorescence	Significant decrease of IRP-1 in striatum and cortex and of IRP-2 in striatum compared to wild-type mice
Aconitase 2	Chen et al., 2017 [42]	Taiwan	Male knock-in HD mice (Hdh(CAG)150, male transgenic R6/2 mice and female control mice (B6CBAFI/J)	Brain sections from the striatum	Spectrophotometry	Significant decrease in the two mice models of HD compared to controls
Copper	Fox et al., 2007 [118]	United States of America and Australia	12-week transgenic R6/2 mice	Brain homogenates from the cortex and striatum	Inductively - coupled plasma mass spectroscopy	Significant increase compared to wild-type mice
Selenium	Lu et al., 2014 [21]	United States of America and Australia	14-week female N171-82Q HD mice and wild-type controls	Plasma	Inductively - coupled plasma mass spectroscopy	Significant increase compared to wild-type mice
**MITOCHONDRIAL RESPIRATORY CHAIN ACTIVITY**						
**PARAMETER**	**AUTHOR, YEAR [REF]**	**COUNTRY**	**EXPERIMENTAL MODEL**	**TISSUE**	**METHOD**	**MAIN FINDINGS**
Mitochondrial dysfunction	Kumar & Kumar 2009 [62]	India	Male Wistar rats weighing 250-300 g. Intraperitoneal administration of 3-NPA	Brain homogenates from the striatum, cortex, and hippocampus	ELISA	Significant increase compared to vehicle controls
Mitochondrial dysfunction	Sandhir et al., 2010 [63]	India	Female Wistar rats weighing 200-250 g. Intraperitoneal administration of 3-NPA	Brain homogenates from the striatum.	Spectrophotometry	Significant increase compared to vehicle controls
Mitochondrial dysfunction	Sandhir et al., 2014 [70]	India	Female Wistar rats weighing 180-200 g. Intraperitoneal administration of 3-NPA	Brain homogenates from the striatum.	Spectrophotometry	Significant increase compared to vehicle controls
Mitochondrial dysfunction	Binawade & Jagtap, 2013 [69]	India	Female Sprague-Dawley rats weighing 200-250. Intraperitoneal administration of 3-NPA	Whole brain homogenates	Spectrophotometry	Significant increase compared to vehicle controls
Mitochondrial dysfunction	Thangarajan et al., 2014 [71] and 2016 [72]	India	Male albino rats weighing 250-300. Intraperitoneal administration of 3-NPA	Brain homogenates from the striatum	4,5-dimethyl-thiazol-2-yl)-2,5-diphenyl-tetrazolium bromide -MTT- colorime-tric assay	Significant increase compared to vehicle controls

Mitochondrial dysfunction	Hariharan et al., 2014 [74]	India	Adult female Wistar rats weighing 200-250 g. Intraperitoneal administration of 3-NPA	Whole brain homogenates (except cerebellum)	Spectrophotometry	Significant increase compared to vehicle controls
Mitochondrial dysfunction	Kalonia et al., 2009 [80]	India	Male Wistar rats weighing 220-250 g. Intrastriatal injection with quinolinic acid	Brain homogenates from the cortex and striatum	Spectrophotometry	Significant increase compared to vehicle controls
Mitochondrial dysfunction	Kalonia et al., 2010 [82]	India	Male Wistar rats weighing 250-300 g. Intrastriatal injection with malonic acid	Brain homogenates from the striatum	Spectrophotometry	Significant increase compared to vehicle controls
Mitochondrial dysfunction	Sumathi et al., 2018 [83]	India	Male albino rats weighing 250-300. Intrastriatal injection with quinolinic acid	Brain homogenates from the striatum	Spectrophotometry	Significant increase compared to vehicle controls
Mitochondrial dysfunction	Pérez-de la Cruz, 2005 [87]	Mexico and the United States of America	Male Wistar weighing 270–300 g	Whole brain homogenates. Brain synaptic vesicles exposed to quinolinic acid	4,5-dimethyl-thiazol-2-yl)-2,5-diphenyl-tetrazolium bromide -MTT-colorimetric assay	Significant increase compared to vehicle controls
Mitochondrial dysfunction	Colle et al., 2012 [88, 120]	Brazil	Male Wistar rats weighing 180-220 g.	Striatal slices incubated with 3-NPA, quinolinic acid, or both	4,5-dimethyl-thiazol-2-yl)-2,5-diphenyl-tetrazolium bromide -MTT-colorimetric assay	Significant increase compared to vehicle controls
NADH de-hydrogenase (complex I activity	Kumar et al., 2006 [61]	India	Male Wistar rats weighing 180-250 g. Intraperitoneal administration of 3-NPA	Whole brain homogenates	Spectrophotometry	Significant decrease compared to vehicle controls
NADH de-hydrogenase (complex I activity	Kumar & Kumar 2009 [62]	India	Male Wistar rats weighing 180-250 g. Intraperitoneal administration of 3-NPA	Whole brain homogenates	Spectrophotometry	Significant decrease compared to vehicle controls
NADH de-hydrogenase (complex I activity	Sandhir et al., 2010 [63]	India	Female Wistar rats weighing 200-250 g. Intraperitoneal administration of 3-NPA	Brain homogenates from the striatum.	Spectrophotometry	Non-significant differences compared to vehicle controls
NADH de-hydrogenase	Sandhir et al., 2014 [70]	India	Female Wistar rats weighing 180-200 g.	Brain homogenates	Spectrophotometry	Significant decrease compared to vehicle

(complex I activity			Intraperitoneal administration of 3-NPA	from the striatum.		controls
NADH de-hydrogenase (complex I activity	Binawade & Jagtap, 2013 [69]	India	Female Sprague-Dawley rats weighing 200-250 mg. Intraperitoneal administration of 3-NPA	Whole brain homogenates	Spectrophotometry	Significant decrease compared to vehicle controls
NADH de-hydrogenase (complex I activity	Thangarajan et al., 2014 [71] and 2016 [72]	India	Male albino rats weighing 250-300. Intraperitoneal administration of 3-NPA	Brain homogenates from the striatum	Spectrophotometry	Significant decrease compared to vehicle controls
NADH de-hydrogenase (complex I activity	Gupta & Sharma, 2014 [73]	India	Adult albino Wistar rats 3-5 months old weighing 200-250 g. Intraperitoneal administration of 3-NPA	Brain homogenates from the striatum	Spectrophotometry	Significant decrease compared to vehicle controls
NADH de-hydrogenase (complex I activity	Hariharan et al., 2014 [74]	India	Adult female Wistar rats weighing 200-250 g. Intraperitoneal administration of 3-NPA	Whole brain homogenates (except cerebellum)	Spectrophotometry	Significant decrease compared to vehicle controls
NADH de-hydrogenase (complex I activity	Kalonia et al., 2009 [80]	India	Male Wistar rats weighing 220-250 g. Intrastriatal injection with quinolinic acid	Brain homogenates from the cortex and striatum	Spectrophotometry	Significant decrease compared to vehicle controls
NADH de-hydrogenase (complex I activity	Sumathi et al., 2018 [83]	India	Male albino rats weighing 250-300. Intrastriatal injection with quinolinic acid	Brain homogenates from the striatum	Spectrophotometry	Significant decrease compared to vehicle controls
NADH de-hydrogenase (complex I activity	Purushothaman & Sumathi, 2022 [84]	India	Male albino rats weighing 250-300. Intrastriatal injection with quinolinic acid	Brain homogenates from the striatum	Spectrophotometry	Significant decrease compared to vehicle controls
NADH de-hydrogenase (complex I activity	Kalonia et al., 2010 [82]	India	Male Wistar rats weighing 250-300 g. Intrastriatal injection with malonic acid	Brain homogenates from the striatum	Spectrophotometry	Non-significant differences compared to vehicle controls
NADH de-hydrogenase (complex I activity	Askeland et al., 2018 [93]	Norway and the Czech Republic	Transgenic minipigs (Sus scrofa domesticus, Linnaeus) with the N-terminal part of human mutated huntingtin and their WT siblings	Frontal cortex, basal ganglia, and PBMC	Fluorometric method	Non-significant differences compared to wild-type
Succinate de-hydrogenase (SDH, complex II) activity	Túnez et al., 2004 [48]	Spain	3-month-old male Wistar rats). Intraperitoneal administration of 3-nitropropionic acid	Striatal and cortical synaptosomes	Colorimetric method	Significant decrease compared to vehicle controls
Succinate de-hydrogenase (SDH, complex II)	Túnez et al., 2006 [60]	Spain and Mexico	3-month-old male Wistar rats). Intraperitoneal administration of 3-	Whole brain homogenates	Colorimetric method	Significant decrease compared to vehicle controls

activity			nitropropionic acid			
Succinate de-hydrogenase (SDH, complex II) activity	Kumar et al., 2006 [61]	India	Male Wistar rats weighing 180-250 g. Intraperitoneal administration of 3-NPA	Whole brain homogenates	Spectrophotometry	Significant decrease compared to vehicle controls
Succinate de-hydrogenase (SDH, complex II) activity	Rosenstock et al., 2009 [121]	Brazil	1-month-old and 18-month-old male B6CBA/F1mice. Intraperitoneal administration of 3-NPA	Slices from the striatum, cortex, and corpus callosum	Immuno-histoche-mistry	Significant decrease compared to vehicle controls in both age groups
Succinate de-hydrogenase (SDH, complex II) activity	Kumar & Kumar 2009 [62]	India	Male Wistar rats weighing 250-300 g. Intraperitoneal administration of 3-NPA	Brain homogenates from the striatum, cortex, and hippocampus	Spectrophotometry	Significant decrease compared to vehicle controls
Succinate de-hydrogenase (SDH, complex II) activity	Sandhir et al., 2010 [63]	India	Female Wistar rats weighing 200-250 g. Intraperitoneal administration of 3-NPA	Brain homogenates from the striatum.	Spectrophotometry	Significant decrease compared to vehicle controls
Succinate de-hydrogenase (SDH, complex II) activity	Chang et al, 2011 [105]	Singapore	16-week-old male Sprague-Dawley rats weighing 290-350 g. Intraperitoneal administration of 3-NPA	Brain homogenates from the striatum, cortex, and hippocampus	Spectrophotometry	Significant decrease compared to vehicle controls
Succinate de-hydrogenase (SDH, complex II) activity	Tasset et al., 2011 [64]	Spain	3-month-old male Wistar rats weighing 220-250 g. Intraperitoneal administration of 3-NPA	Striatum and the rest of the brain homogenates.	Spectrophotometry	Significant decrease compared to vehicle controls
Succinate de-hydrogenase (SDH, complex II) activity	Bhateha et al., 2012 [66]	India	Male Wistar rats weighing 180-220 g. Intraperitoneal administration of 3-NPA	Whole brain homogenates	Spectrophotometry	Significant decrease compared to vehicle controls
Succinate de-hydrogenase (SDH, complex II) activity	Sandhir et al., 2014 [70]	India	Female Wistar rats weighing 180-200 g. Intraperitoneal administration of 3-NPA	Brain homogenates from the striatum.	Spectrophotometry	Significant decrease compared to vehicle controls
Succinate de-hydrogenase (SDH, complex II) activity	Binawade & Jagtap, 2013 [69]	India	Female Sprague-Dawley rats weighing 200-250. Intraperitoneal administration of 3-NPA	Whole brain homogenates	Spectrophotometry	Significant decrease compared to vehicle controls
Succinate de-hydrogenase (SDH, complex II) activity	Gupta & Sharma, 2014 [73]	India	Adult albino Wistar rats 3-5 months old weighing 200-250 g. Intraperitoneal administration of 3-NPA	Brain homogenates from the striatum	Spectrophotometry	Significant decrease compared to vehicle controls

Succinate de-hydrogenase (SDH, complex II) activity	Souza et al., 2014 [97]	Brazil	Male Wistar rats weighing 200-250 g. Intraperitoneal administration of 3-NPA	Brain homogenates from the striatum	Spectrophotometry	Significant decrease compared to vehicle controls
Succinate de-hydrogenase (SDH, complex II) activity	Hariharan et al., 2014 [74]	India	Adult female Wistar rats weighing 200-250 g. Intraperitoneal administration of 3-NPA	Whole brain homogenates (except cerebellum)	Spectrophotometry	Significant decrease compared to vehicle controls
Succinate de-hydrogenase (SDH, complex II) activity	Thangarajan et al., 2014 [71] and 2016 [72]	India	Male albino rats weighing 250-300. Intraperitoneal administration of 3-NPA	Brain homogenates from the striatum	Spectrophotometry	Significant decrease compared to vehicle controls
Succinate de-hydrogenase (SDH, complex II) activity	Jang & Chang 2016 [107]	South Korea	MaleC57BL/6 mice weighing 23-25 g). Intraperitoneal administration of 3-NPA	Striatal slices	Immunohistochemistry	Significant decrease compared to vehicle controls
Succinate de-hydrogenase (SDH, complex II) activity	Silva-Palacios et al., 2017 [77]	Mexico	Adult (9 months-old) and old (24 months-old) albino Wistar female rats. Intraperitoneal administration of 3-NPA	Brain homogenates from the striatum	Spectrophotometry	Significant decrease compared to vehicle controls
Succinate de-hydrogenase (SDH, complex II) activity	Kalonia et al., 2009 [80]	India	Male Wistar rats weighing 220-250 g. Intrastriatal injection with quinolinic acid	Brain homogenates from the cortex and striatum	Spectrophotometry	Significant decrease compared to vehicle controls
Succinate de-hydrogenase (SDH, complex II) activity	Sumathi et al., 2018 [83]	India	Male albino rats weighing 250-300. Intrastriatal injection with quinolinic acid	Brain homogenates from the striatum	Spectrophotometry	Significant decrease compared to vehicle controls
Succinate de-hydrogenase (SDH, complex II) activity	Purushothaman & Sumathi, 2022 [84]	India	Male albino rats weighing 250-300. Intrastriatal injection with quinolinic acid	Brain homogenates from the striatum	Spectrophotometry	Significant decrease compared to vehicle controls
Succinate de-hydrogenase (SDH, complex II) activity	Kalonia et al., 2010 [82]	India	Male Wistar rats weighing 250-300 g. Intrastriatal injection with malonic acid	Brain homogenates from the striatum	Spectrophotometry	Significant decrease compared to vehicle controls
Succinate de-hydrogenase (SDH, complex II) activity	Johri et al., 2012 [90]	United States of America	Transgenic R6/2 mice and their wild-type littermates	Muscle	Immunohistochemistry	Significant decrease compared to wild-type
Succinate de-hydrogenase (SDH, complex II) activity	Pinho et al., 2020 [101]	Portugal and the United Kingdom	11-week male transgenic R6/2 mice (B6CBA-Tg(HDexon1) 62Gpb/3J), and male wild-type (WT)	Whole brain homogenates, liver and muscle homogenates	Western blot	Non-significant differences in brain, liver, and muscle, compared to wild-type mice

			B6CBAF1/J mice			
Succinate de-hydrogenase (SDH, complex II) activity	Askeland et al., 2018 [93]	Norway and the Czech Republic	Transgenic minipigs (Sus scrofa domesticus, Linnaeus) with the N-terminal part of human mutated huntingtin and their WT siblings	Frontal cortex, basal ganglia, and PBMC	Fluorometric method	Non-significant differences compared to wild-type
Cytochrome c oxidoreduc-tase (QCCR, complex III),	Askeland et al., 2018 [93]	Norway and the Czech Republic	Transgenic minipigs (Sus scrofa domesticus, Linnaeus) with the N-terminal part of human mutated huntingtin and their WT siblings	Frontal cortex, basal ganglia, and PBMC	Fluorometric method	Non-significant differences compared to wild-type
Cytochrome oxidase (complex IV) activity	Kumar & Kumar 2009 [62]	India	Male Wistar rats weighing 250-300 g. Intraperitoneal administration of 3-NPA	Brain homogenates from the striatum, cortex, and hippocampus	Spectrophotometry	Significant decrease compared to vehicle controls
Cytochrome oxidase (complex IV) activity	Sandhir et al., 2010 [63]	India	Female Wistar rats weighing 200-250 g. Intraperitoneal administration of 3-NPA	Brain homogenates from the striatum.	Spectrophotometry	Significant decrease compared to vehicle controls
Cytochrome oxidase (complex IV) activity	Sandhir et al., 2014 [70]	India	Female Wistar rats weighing 180-200 g. Intraperitoneal administration of 3-NPA	Brain homogenates from the striatum.	Spectrophotometry	Significant decrease compared to vehicle controls
Cytochrome oxidase (complex IV) activity	Binawade & Jagtap, 2013 [69]	India	Female Sprague-Dawley rats weighing 200-250	Whole brain homogenates	Spectrophotometry	Significant decrease compared to vehicle controls
Cytochrome oxidase (complex IV) activity	Gupta & Sharma, 2014 [73]	India	Adult albino Wistar rats 3-5 months old weighing 200-250 g. Intraperitoneal administration of 3-NPA	Brain homogenates from the striatum	Spectrophotometry	Significant decrease compared to vehicle controls
Cytochrome oxidase (complex IV) activity	Hariharan et al., 2014 [74]	India	Adult female Wistar rats weighing 200-250 g. Intraperitoneal administration of 3-NPA	Whole brain homogenates (except cerebellum)	Spectrophotometry	Significant decrease compared to vehicle controls
Cytochrome oxidase (complex IV) activity	Thangarajan et al., 2014 [71] and 2016 [72]	India	Male albino rats weighing 250-300. Intraperitoneal administration of 3-NPA	Brain homogenates from the striatum	Spectrophotometry	Significant decrease compared to vehicle controls
Cytochrome oxidase (complex IV) activity	Kalonia et al., 2009 [80]	India	Male Wistar rats weighing 220-250 g. Intrastriatal injection with quinolinic acid	Brain homogenates from the cortex and striatum	Spectrophotometry	Significant decrease compared to vehicle controls
Cytochrome oxidase (complex IV)	Sumathi et al., 2018 [83]	India	Male albino rats weighing 250-300. Intrastriatal injection	Brain homogenates from the	Spectrophotometry	Significant decrease compared to vehicle controls

activity			with quinolinic acid	striatum		
Cytochrome oxidase (complex IV) activity	Purushothaman & Sumathi, 2022 [84]	India	Male albino rats weighing 250-300. Intrastriatal injection with quinolinic acid	Brain homogenates from the striatum	Spectrophotometry	Significant decrease compared to vehicle controls
Cytochrome oxidase (complex IV) activity	Kalonia et al., 2010 [82]	India	Male Wistar rats weighing 250-300 g. Intrastriatal injection with malonic acid	Brain homogenates from the striatum	Spectrophotometry	Non-significant difference compared to vehicle controls
Cytochrome oxidase (complex IV) activity	Lou et al., 2016 [100]	United States of America	9 week-old R6/2 mice (transgenic for the HD mutation)	Brain homogenates from the cortex and striatum	Spectrophotometry	Non-significant differences compared to wild-type mice
ATPase (complex V) activity	Sandhir et al., 2010 [63]	India	Female Wistar rats weighing 200-250 g. Intraperitoneal administration of 3-NPA	Brain homogenates from the striatum.	Spectrophotometry	Significant decrease compared to vehicle controls
ATPase (complex V) activity	Binawade & Jagtap, 2013 [69]	India	Female Sprague-Dawley rats weighing 200-250. Intraperitoneal administration of 3-NPA	Whole brain homogenates	Spectrophotometry	Significant decrease compared to vehicle controls
ATPase (complex V) activity	Sandhir et al., 2014 [70]	India	Female Wistar rats weighing 180-200 g. Intraperitoneal administration of 3-NPA	Brain homogenates from the striatum.	Spectrophotometry	Significant decrease compared to vehicle controls
ATPase (complex V) activity	Thangarajan et al., 2014 [71] and 2016 [72]	India	Male albino rats weighing 250-300. Intraperitoneal administration of 3-NPA	Brain homogenates from the striatum	Spectrophotometry	Significant decrease compared to vehicle controls
ATPase (complex V) activity	Sumathi et al., 2018 [83]	India	Male albino rats weighing 250-300. Intrastriatal injection with quinolinic acid	Brain homogenates from the striatum	Spectrophotometry	Significant decrease compared to vehicle controls
**PROTEINS AND ENZYMES PROTECTIVE AGAINST OXIDATIVE STRESS**						
**PARAMETER**	**AUTHOR, YEAR [REF]**	**COUNTRY**	**EXPERIMENTAL MODEL**	**TISSUE**	**METHOD**	**MAIN FINDINGS**
Total SOD activity	Túnez et al., 2004 [48]	Spain	3-month-old male Wistar rats). Intraperitoneal administration of 3-NPA	Striatal and cortical synaptosomes	Spectrophotometry	Significant increase compared to vehicle controls
Total SOD activity	Kumar & Kumar 2009 [62]	India	Male Wistar rats weighing 250-300 g. Intraperitoneal administration of 3-NPA	Brain homogenates from the striatum, cortex, and hippocampus	Spectrophotometry	Significant decrease compared to vehicle controls
Total SOD activity	Chang et al, 2011 [105]	Singapore	16-week-old male Sprague-Dawley rats weighing 290-350 g. Intraperitoneal	Brain homogenates from striatum, cortex and	Spectrophotometry	Significant decrease in brain homogenates compared to vehicle

			administration of 3-NPA	hippocampus, and plasma		controls. Non-significant differences in plasma
Total SOD activity	Gopinath et al., 2011 [65]	India	Male Wistar rats weighing 250-300 g. Intraperitoneal administration of 3-NPA	Brain homogenates from the striatum and plasma	Enzymatic assay. Spectro-photo-metry	Significant decrease compared to vehicle controls
Total SOD activity	Bhateha et al., 2012 [66]	India	Male Wistar rats weighing 180-220 g. Intraperitoneal administration of 3-NPA	Whole brain homogenates	Spectrophotometry	Significant decrease compared to vehicle controls
Total SOD activity	Denny-Joseph & Muralidhara, 2013 [68]	India	4-week-old male Wistar Intraperitoneal administration of 3-NPA	Brain homogenates from the striatum and cerebellum	Fluores-cence. Spectrophotometry	Significant decrease compared to vehicle controls
Total SOD activity	Sandhir et al., 2014 [70]	India	Female Wistar rats weighing 180-200 g. Intraperitoneal administration of 3-NPA	Brain homogenates from the striatum.	Spectrophotometry	Significant decrease compared to vehicle controls
Total SOD activity	Gupta & Sharma, 2014 [73]	India	Adult albino Wistar rats 3-5 months old weighing 200-250 g. Intraperitoneal administration of 3-NPA	Brain homogenates from the striatum	Spectrophotometry	Significant decrease compared to vehicle controls
Total SOD activity	Thangarajan et al., 2014 [71] and 2016 [72]	India	Male albino rats weighing 250-300. Intraperitoneal administration of 3-NPA	Brain homogenates from the striatum	Spectrophotometry	Significant decrease compared to vehicle controls
Total SOD activity	Badini et al., 2024 [78]	Iran	Male Wistar rats weighing 220-250 g. Intraperitoneal administration of 3-NPA	Whole brain homogenates	Spectrophotometry	Significant decrease compared to vehicle controls
Total SOD activity	Pérez-de la Cruz, 2005 [85]	Mexico and the United States of America	Male Wistar weighing 270–300 g. Intrastriatal injection of quinolinic acid.	Striatum	Spectrophotometry	Significant decrease compared to vehicle controls
Total SOD activity	Kalonia et al., 2009 [80]	India	Male Wistar rats weighing 220-250 g. Intrastriatal injection with quinolinic acid	Brain homogenates from the cortex and striatum	Spectrophotometry	Significant decrease compared to vehicle controls
Total SOD activity	Antunes-Wilhelm et al, 2013 [99]	Brazil	Male Wistar rats weighing 200–250 g. Intrastriatal injection with quinolinic acid	Brain homogenates from the striatum	Spectrophotometry	Significant decrease compared to vehicle controls
Total SOD activity	Sumathi et al., 2018 [83]	India	Male albino rats weighing 250-300. Intrastriatal injection	Brain homogenates from the	Spectrophotometry	Significant decrease compared to vehicle controls

			with quinolinic acid	striatum		
Total SOD activity	Purushothaman & Sumathi, 2022 [84]	India	Male albino rats weighing 250-300. Intrastriatal injection with quinolinic acid	Brain homogenates from the striatum	Spectrophotometry	Significant decrease compared to vehicle controls
Total SOD activity	Santamaría et al., 2001 [122]	México	Male Wistar rats weighing 250-300 g. Striatal injection with quinolinic acid or 3-NPA	Brain homogenates from the striatum	Enzymatic assay	Significant decrease with both toxins compared to vehicle controls
Total SOD activity	Kalonia et al., 2010 [82]	India	Male Wistar rats weighing 250-300 g. Intrastriatal injection with malonic acid	Brain homogenates from the striatum	Spectrophotometry	Significant decrease compared to vehicle controls
Total SOD activity	Santamaría et al., 2001 [122]	México	19-week and 35-week transgenic R6/1 mice	Brain homogenates from the striatum	Enzymatic assay	Significant increase at 19-week and decrease at 35-week compared with wild-type mice
Total SOD activity	Fox et al., 2007 [118]	United States of America and Australia	12-week transgenic R6/2 mice	Brain homogenates from the cortex and striatum	Enzymatic assay	Non-significant differences compared to wild-type mice
Total SOD activity	Dominah et al., 2017 [94]	United States of America	*STHdhQ7/Q7 and STHdhQ111/Q111* striatal cell lines. Incubation with chlorpyrifos (CPF)	Cellular cultures	Spectrophotometry	Significant increase compared to wild-type
Cu/Zn-SOD activity	Santamaría et al., 2001 [122]	México	Male Wistar rats weighing 250-300 g. Striatal injection with quinolinic acid or 3-NPA	Brain homogenates from the striatum	Enzymatic assay	Significant decrease with both toxins compared to vehicle controls
Cu/Zn SOD activity	Santamaría et al., 2001 [122]	México	19-week and 35-week transgenic R6/1 mice	Brain homogenates from the striatum	Enzymatic assay	Significant increase at 19-week and decrease at 35-week compared with wild-type mice
Cu/Zn SOD mRNA expresión	Szlachcic et al., 2015 [123]	Poland	Induced pluripotent stem cells (iPSCs) from fibroblasts of humans with HD	Cellular cultures	Real-time qPCR	Increased expression compared to controls
Mn SOD activity	Santamaría et al., 2001 [122]	México	Male Wistar rats weighing 250-300 g. Striatal injection with quinolinic acid or 3-NPA	Brain homogenates from the striatum	Enzymatic assay	Significant decrease with 3-NPA, but not with quinolinic acid, compared to vehicle controls
Mn-SOD activity	Santamaría et al., 2001 [122]	México	19-week and 35-week transgenic R6/1 mice	Brain homogenates from the striatum	Enzymatic assay	Non-significant differences at 19-week and 35-week compared with wild-type mice.
Mn-SOD activity	Pinho et al., 2020 [101]	Portugal and the United Kingdom	11-week male transgenic R6/2 mice (B6CBA-Tg(HDexon1) 62Gpb/3J), and male	Whole brain homogenates, liver and muscle	Mass spectrometry	Significant increase in brain, liver, and muscle compared to wild-type mice

			wild-type (WT) B6CBAF1/J mice	homogenates		
Mn-SOD protein	Peterson et al., 2022 [110]	Denmark	11 week male transgenic R6/2 mice, and young R6/2 males and young R62 mice backcrossed with healthy CBAxC57BL-6 hybrid females	Striatal and cortical synaptosomes	Fluorescence method	Significant increase compared to wild-type mice in striatal, but not in cortical synaptosomes
GPx activity	Túnez et al., 2006 [60]	Spain and Mexico	3-month-old male Wistar rats. Intraperitoneal administration of 3-NPA	Whole brain homogenates	Spectrophotometry	Significant decrease compared to vehicle controls
GPx activity	Gopinath et al., 2011 [65]	India	Male Wistar rats weighing 250-300 g. Intraperitoneal administration of 3-NPA	Brain homogenates from the striatum and plasma	Enzymatic assay. Spectro-photo-metry	Significant decrease compared to vehicle controls
GPx activity	Souza et al., 2014 [97]	Brazil	Male Wistar rats weighing 200-250 g. Intraperitoneal administration of 3-NPA	Brain homogenates from the striatum	Spectrophotometry	Significant decrease compared to vehicle controls
GPx activity	Tasset et al., 2011 [64]	Spain	Murine neuroblastoma N1E-115 cells incubated with 3-NPA	Murine neuroblastoma N1E-115 cells incubated with 3-NPA	Spectrophotometry	Significant decrease compared to vehicle controls
GPx activity	Sumathi et al., 2018 [83]	India	Male albino rats weighing 250-300. Intrastriatal injection with quinolinic acid	Brain homogenates from the striatum	Spectrophotometry	Significant decrease compared to vehicle controls
GPx activity	Purushothaman & Sumathi, 2022 [84]	India	Male albino rats weighing 250-300. Intrastriatal injection with quinolinic acid	Brain homogenates from the striatum	Spectrophotometry	Significant decrease compared to vehicle controls
GPx activity	Ribeiro et al., 2012 [113]	Portugal	Striatal cells expressing wild-type Htt (STHdhQ7/Q7 or wild-type cells; clone 2aA5) or homozygous mutant cells derived from knock-in mice, expressing FL-mHtt with 111 glutamines (STHdhQ111/ Q111cells; clone 109-1A),	Cellular cultures	Spectrophotometry	Significant increase compared to wild-type cells
GPx-1 expression	Szlachcic et al., 2015 [123]	Poland	Induced pluripotent stem cells (iPSCs) from fibroblasts of YAC128 HD transgenic mice	Cellular cultures	Real-time qPCR	Increased expression compared to wild-type
CAT activity	Túnez et al., 2006 [60]	Spain and Mexico	3-month-old male Wistar rats). Intraperitoneal	Whole brain homogenates	Spectrophotometry	Significant decrease compared to vehicle controls

			administration of 3-NPA			
CAT activity	Kumar & Kumar 2009 [62]	India	Male Wistar rats weighing 250-300 g. Intraperitoneal administration of 3-NPA	Brain homogenates from the striatum, cortex, and hippocampus	Spectrophotometry	Significant decrease compared to vehicle controls
CAT activity	Gopinath et al., 2011 [65]	India	Male Wistar rats weighing 250-300 g. Intraperitoneal administration of 3-NPA	Brain homogenates from the striatum and plasma	Enzymatic assay. Spectro-photo-metry	Significant decrease compared to vehicle controls
CAT activity	Bhateha et al., 2012 [66]	India	Male Wistar rats weighing 180-220 g. Intraperitoneal administration of 3-NPA	Whole brain homogenates	Spectrophotometry	Significant decrease compared to vehicle controls
CAT activity	Denny-Joseph & Muralidhara, 2013 [68]	India	4-week-old male Wistar Intraperitoneal administration of 3-NPA	Brain homogenates from the striatum and cerebellum	Fluores-cence. Spectrophotometry	Non. significant differences compared to vehicle controls
CAT activity	Shivasharan et al., 2012 [67]	India	Adult female Wistar rats weighing 190-200 g. Intraperitoneal administration of 3-NPA	Whole brain homogenates	Spectrophotometry	Significant decrease compared to vehicle controls
CAT activity	Thangarajan et al., 2014 [71] and 2016 [72]	India	Male albino rats weighing 250-300. Intraperitoneal administration of 3-NPA	Brain homogenates from the striatum	Spectrophotometry	Significant decrease compared to vehicle controls
CAT activity	Gupta & Sharma, 2014 [73]	India	Adult albino Wistar rats 3-5 months old weighing 200-250 g. Intraperitoneal administration of 3-NPA	Brain homogenates from the striatum	Spectrophotometry	Significant decrease compared to vehicle controls
CAT activity	Hariharan et al., 2014 [74]	India	Adult female Wistar rats weighing 200-250 g. Intraperitoneal administration of 3-NPA	Whole brain homogenates (except cerebellum)	Spectrophotometry	Significant decrease compared to vehicle controls
CAT activity	Khan et al., 2015 [75]	India	Adult female Wistar rats weighing 250-300 g. Intraperitoneal administration of 3-NPA	Brain homogenates from the striatum	Colorimetric method and spectrophotometry	Significant decrease compared to vehicle controls
CAT activity	Tasset et al., 2011 [64]	Spain	Murine neuroblastoma N1E-115 cells incubated with 3-NPA	Murine neuroblastoma N1E-115 cells incubated with 3-NPA	Spectrophotometry	Significant decrease compared to vehicle controls
CAT activity	Kalonia et al., 2009 [80]	India	Male Wistar rats weighing 220-250 g. Intrastriatal injection	Brain homogenates from the	Spectrophotometry	Significant decrease compared to vehicle controls

			with quinolinic acid	cortex and striatum		
CAT activity	Antunes-Wilhelm et al, 2013 [99]	Brazil	Male Wistar rats weighing 200–250 g. Intrastriatal injection with quinolinic acid	Brain homogenates from the striatum	Spectrophotometry	Significant decrease compared to vehicle controls
CAT activity	Sumathi et al., 2018 [83]	India	Male albino rats weighing 250-300. Intrastriatal injection with quinolinic acid	Brain homogenates from the striatum	Spectrophotometry	Significant decrease compared to vehicle controls
CAT activity	Purushothaman & Sumathi, 2022 [84]	India	Male albino rats weighing 250-300. Intrastriatal injection with quinolinic acid	Brain homogenates from the striatum	Spectrophotometry	Significant decrease compared to vehicle controls
CAT activity	Kalonia et al., 2010 [82]	India	Male Wistar rats weighing 250-300 g. Intrastriatal injection with malonic acid	Brain homogenates from the striatum	Spectrophotometry	Significant decrease compared to vehicle controls
GSH	Túnez et al., 2006 [60]	Spain and Mexico	3-month-old male Wistar rats). Intraperitoneal administration of 3-NPA	Whole brain homogenates	Spectrophotometry	Significant decrease compared to vehicle controls
GSH	Kumar et al., 2006 [61]	India	Male Wistar rats weighing 180-250 g. Intraperitoneal administration of 3-NPA	Whole brain homogenates	Spectrophotometry	Significant decrease compared to vehicle controls
GSH	Tasset et al., 2011 [64]	Spain	3-month-old male Wistar rats weighing 220-250 g. Intraperitoneal administration of 3-NPA	Striatum and the rest of the brain homogenates.	Spectrophotometry	Significant decrease compared to vehicle controls
GSH	Gopinath et al., 2011 [65]	India	Male Wistar rats weighing 250-300 g. Intraperitoneal administration of 3-NPA	Brain homogenates from the striatum and plasma	Enzymatic assay. Spectro-photometry	Significant decrease compared to vehicle controls
GSH	Bhateha et al., 2012 [66]	India	Male Wistar rats weighing 180-220 g. Intraperitoneal administration of 3-NPA	Whole brain homogenates	Spectrophotometry	Significant decrease compared to vehicle controls
GSH	Shivasharan et al., 2012 [67]	India	Adult female Wistar rats weighing 190-200 g. Intraperitoneal administration of 3-NPA	Whole brain homogenates	Spectrophotometry	Significant decrease compared to vehicle controls
GSH	Sandhir et al., 2014 [70]	India	Female Wistar rats weighing 180-200 g. Intraperitoneal administration of 3-NPA	Brain homogenates from the striatum.	Spectrophotometry	Significant decrease compared to vehicle controls
GSH	Thangarajan et al., 2014	India	Male albino rats weighing 250-300.	Brain homogenates	Spectrophotometry	Significant decrease compared to vehicle

	[71] and 2016 [72]		Intraperitoneal administration of 3-NPA	from the striatum		controls
GSH	Gupta & Sharma, 2014 [73]	India	Adult albino Wistar rats 3-5 months old weighing 200-250 g. Intraperitoneal administration of 3-NPA	Brain homogenates from the striatum	Spectrophotometry	Significant decrease compared to vehicle controls
GSH	Hariharan et al., 2014 [74]	India	Adult female Wistar rats weighing 200-250 g. Intraperitoneal administration of 3-NPA	Whole brain homogenates (except cerebellum)	Spectrophotometry	Significant decrease compared to vehicle controls
GSH	Khan et al., 2015 [75]	India	Adult female Wistar rats weighing 250-300 g. Intraperitoneal administration of 3-NPA	Brain homogenates from the striatum	Spectrophotometry	Significant decrease compared to vehicle controls
GSH	Badini et al.,, 2024 [78]	Iran	Male Wistar rats weighing 220-250 g. Intraperitoneal administration of 3-NPA	Whole brain homogenates	Spectrophotometry	Significant decrease compared to vehicle controls
GSH	Tasset et al., 2011 [64]	Spain	Murine neuroblastoma N1E-115 cells incubated with 3-NPA	Murine neuroblastoma N1E-115 cells incubated with 3-NPA	Spectrophotometry	Significant decrease compared to vehicle controls
GSH	Maksimović et al., 2001 [124]	Serbia	Rats. Intrastriatal injection with quinolinic acid	Striatum and hippocampus	Spectrophotometry	Significant decrease compared to vehicle controls
GSH	Tkác et al., 2001 [125]	United States of America	Female Fischer 344 rats weighing 150-200 g. Intrastriatal injection with quinolinic acid	Striatum	^{1}H-NMR-spectroscopy	Significant increase compared to wild-type
GSH	Kalonia et al., 2009 [80]	India	Male Wistar rats weighing 220-250 g. Intrastriatal injection with quinolinic acid	Brain homogenates from the cortex and striatum	Spectrophotometry	Significant decrease compared to vehicle controls
GSH	Sumathi et al., 2018 [83]	India	Male albino rats weighing 250-300. Intrastriatal injection with quinolinic acid	Brain homogenates from the striatum	Spectrophotometry	Significant decrease compared to vehicle controls
GSH	Purushothaman & Sumathi, 2022 [84]	India	Male albino rats weighing 250-300. Intrastriatal injection with quinolinic acid	Brain homogenates from the striatum	Spectrophotometry	Significant decrease compared to vehicle controls
GSH	Leipnitz et al. [86]	Brazil	30-days Wistar rats	Homogenates from the cerebral cortex incubated with quinolinic acid	Fluorescence method	Significant decrease compared to vehicle controls

GSH	Kalonia et al., 2010 [82]	India	Male Wistar rats weighing 250-300 g. Intrastriatal injection with malonic acid	Brain homogenates from the striatum	Spectrophotometry	Significant decrease compared to vehicle controls
GSH	Choo et al., 2005 [126]	United States of America	R6/2 transgenic HD mice and littermate wild-type controls	Brain homogenates from the cortex and striatum	Spectrophotometry	Significant decrease in mitochondria compared to wild-type
GSH	Lu et al., 2014 [21]	United States of America and Australia	14-week female N171-82Q HD mice and wild-type controls	Cortex and striatal slices and plasma	Chromogenic method	Non-significant changes in the brain, and a significant increase in plasma compared to wild-type
GSH	Fernández et al., 2023 [111]	Spain	2-months and 7-months R6/1 transgenic HC mice and wild-type controls	Primary cultures of chromaffin cells from the adrenal medulla	Spectrophotometry	Non-significant differences compared to age-matched wild-type mice, decrease with age in both groups.
GSH	Hong et al., 2015 [127]	United States of America and South Korea	Yeast artificial chromosome 128 (YAC 128) line of transgenic mice	Brain homogenates from the striatum	Fluorescence method	Significant decrease compared to wild-type
GSH	Chandra et al..2016 [91]	United States of America	BACHD founder mice expressing expanded human Htt with 97 mixed CAA–CAG repeats	Brain homogenates from the striatum	HPLC with electrochemical detection	Significant decrease compared to wild-type mice
GSH	Ribeiro et al., 2012 [113]	Portugal	Striatal cells expressing wild-type Htt (STHdhQ7/Q7 or wild-type cells; clone 2aA5) or homozygous mutant cells derived from knock-in mice, expressing FL-mHtt with 111 glutamines (STHdhQ111/ Q111cells; clone 109-1A),	Cellular cultures	Spectrofluorimetry	Significant increase compared to wild-type cells
GSH	Frederick et al., 2014 [114]	France, the United States of America, and Georgia	*STHdhQ7/Q7 and STHdhQ111/Q111* striatal cell lines, derived from neuronal precursor cells isolated from knock-in mice	Cellular cultures	Spectrophotometry	Significant decrease compared to wild-type cells
GSH	Dominah et al., 2017 [94]	United States of America	*STHdhQ7/Q7 and STHdhQ111/Q111* striatal cell lines. Incubation with chlorpyrifos (CPF)	Cellular cultures	Spectrophotometry	Significant decrease compared to wild-type
GSH	Pruccoli et al., 2021 [117]	Italy and the United Kingdom	Inducible rat pheochromocytoma (PC12) cells expressing	Cellular cultures	Fluorescence method with a	Significant increase in HD-Q74 but not in HD-Q23 cells

			the HTT gene with 23 or 74 glutamine repeats (HD-Q23 or HD-Q74)		multilabel plate reader	
GSSG	Kalonia et al., 2009 [80]	India	Male Wistar rats weighing 220-250 g. Intrastriatal injection with quinolinic acid	Brain homogenates from the cortex and striatum	Spectrophotometry	Significant increase compared to vehicle controls
GSSG	Kalonia et al., 2010 [82]	India	Male Wistar rats weighing 250-300 g. Intrastriatal injection with malonic acid	Brain homogenates from the striatum	Spectrophotometry	Significant increase compared to vehicle controls
GSSG	Fernández et al., 2023 [111]	Spain	2-months and 7-months R6/1 transgenic HC mice and wild-type controls	Primary cultures of chromaffin cells from the adrenal medulla	Spectrophotometry	Significant increase compared to age-matched wild-type mice, and increase with age in both groups.
GSSG	Lu et al., 2014 [21]	United States of America and Australia	14-week female N171-82Q HD mice and wild-type controls	Cortex and striatal slices and plasma	Chromogenic method	Significant increase compared to wild-type
GSSG	Hong et al., 2015 [127]	United States of America and South Korea	Yeast artificial chromosome 128 (YAC 128) line of transgenic mice	Brain homogenates from the striatum	Fluorescence method	Significant increase compared to wild-type
GSSG	Ribeiro et al., 2012 [113]	Portugal	Striatal cells expressing wild-type Htt (STHdhQ7/Q7 or wild-type cells; clone 2aA5) or homozygous mutant cells derived from knock-in mice, expressing FL-mHtt with 111 glutamines (STHdhQ111/ Q111cells; clone 109-1A),	Cellular cultures	Spectrofluorimetry	Significant increase compared to wild-type cells
GSH/GSSG ratio	Ooi et al., 2015 [46]	Singapore and Canada	Striatal cells expressing STHdhQ7/Q7(wild-type) and STHdhQ111/Q111(mutant for HD)	Cellular cultures	Luciferase assay and luminescence	Significant increase compared to wild-tyè
GR activity	Gopinath et al., 2011 [65]	India	Male Wistar rats weighing 250-300 g. Intraperitoneal administration of 3-NPA	Brain homogenates from the striatum and plasma	Enzymatic assay. Spectro-photo-metry	Significant decrease compared to vehicle controls
GR activity	Souza et al., 2014 [97]	Brazil	Male Wistar rats weighing 200-250 g. Intraperitoneal administration of 3-NPA	Brain homogenates from the striatum	Spectrophotometry	Significant increase compared to vehicle controls
GR activity	Sumathi et al., 2018 [83]	India	Male albino rats weighing 250-300. Intrastriatal injection with quinolinic acid	Brain homogenates from the striatum	Spectrophotometry	Significant decrease compared to vehicle controls

GR activity	Ribeiro et al., 2012 [113]	Portugal	Striatal cells expressing wild-type Htt (STHdhQ7/Q7 or wild-type cells; clone 2aA5) or homozygous mutant cells derived from knock-in mice, expressing FL-mHtt with 111 glutamines (STHdhQ111/ Q111cells; clone 109-1A),	Cellular cultures	Spectrophotometry	Significant increase compared to wild-type cells
GR mRNA and protein	Chandra et al..2016 [91]	United States of America	BACHD founder mice expressing expanded human Htt with 97 mixed CAA–CAG repeats	Brain homogenates from the striatum	Real-time PCR and Western blot	Significant decrease compared to wild-type mice
GST activity	Denny-Joseph & Muralidhara, 2013 [68]	India	4-week-old male Wistar Intraperitoneal administration of 3-NPA	Brain homogenates from the striatum and cerebellum	Fluorescence. Spectrophotometry	Significant decrease compared to vehicle controls in the cerebellum
GST activity	Shivasharan et al., 2012 [67]	India	Adult female Wistar rats weighing 190-200 g. Intraperitoneal administration of 3-NPA	Whole brain homogenates	Spectrophotometry	Significant decrease compared to vehicle controls
GST activity	Souza et al., 2014 [97]	Brazil	Male Wistar rats weighing 200-250 g. Intraperitoneal administration of 3-NPA	Brain homogenates from the striatum	Spectrophotometry	Non-significant changes compared to vehicle controls
GST activity	Sumathi et al., 2018 [83]	India	Male albino rats weighing 250-300. Intrastriatal injection with quinolinic acid	Brain homogenates from the striatum	Spectrophotometry	Significant decrease compared to vehicle controls
GST activity	Ribeiro et al., 2012 [113]	Portugal	Striatal cells expressing wild-type Htt (STHdhQ7/Q7 or wild-type cells; clone 2aA5) or homozygous mutant cells derived from knock-in mice, expressing FL-mHtt with 111 glutamines (STHdhQ111/ Q111cells; clone 109-1A),	Cellular cultures	Spectrophotometry	Non-significant differences compared to wild-type cells
Glutathione synthetase (GS) activity	Ribeiro et al., 2012 [113]	Portugal	Striatal cells expressing wild-type Htt (STHdhQ7/Q7 or wild-type cells; clone 2aA5) or homozygous mutant cells derived from knock-in mice, expressing FL-mHtt with 111 glutamines	Cellular cultures	Spectrofluorimetry	Significant decrease compared to wild-type cells

			(STHdhQ111/ Q111cells; clone 109-1A),			
Glucose-6-phosphate dihydrogen-ase (G6PD) and 6-phospho-gluconate dihydrogen-ase (6PGD) activities	Ribeiro et al., 2012 [113]	Portugal	Striatal cells expressing wild-type Htt (STHdhQ7/Q7 or wild-type cells; clone 2aA5) or homozygous mutant cells derived from knock-in mice, expressing FL-mHtt with 111 glutamines (STHdhQ111/ Q111cells; clone 109-1A),	Cellular cultures	Spectropho tometry	Significant decrease compared to wild-type cells
GADPH	Jang & Cho, 2016 [107]	South Korea	Male C57BL/6 mice weighing 23-25 g). Intraperitoneal administration of 3-NPA	Striatal slices	Immuno-histoche-mistry	Significant decrease compared to vehicle controls
Glycerophos-phocholine phosphodieste rase 1 (GPCPD1) expression	Chang et al., 2024 [24]	Taiwan	Heterozygous R6/2 transgenic mice and female control mice (B6CBAFI/J)	Brain homogenates from the striatum and cortex	Real-time quantita-tive PCRs (qPCRs) and immuno-histo-chemistry	Significant decrease compared to wild-type
Glycogen synthase kinase-3β (GSK-3β) expression	L'Episcopo et al., 2016 [23]	Italy, Sweden, Canada, and the United Kingdom	2.5–3 weeks and 48 weeks-old R6/2 transgenic mice and wild.type littermates.	Primary neuronal and primary astrocytic cultures	Lumines-cence spectropho tometry and western blot	Increased expression of GSK-3β and its active metabolite pGSK-3β-Tyr21 compared to wild-type
MAO-A mRNA and activity	Ooi et al., 2015 [46]	Singapore and Canada	Striatal cells expressing STHdhQ7/Q7(wild-type) and STHdhQ111/Q111(mutant for HD)	Cellular cultures	Luciferase assay and luminescence	Significant increase compared to wild-type
Thioredoxin-reductase (TRR) activity	Denny-Joseph & Muralidhara, 2013 [68]	India	4-week-old male Wistar Intraperitoneal administration of 3-NPA	Brain homogenates from the striatum and cerebellum	Fluores-cence. Spectropho tometry	Non-significant differences compared to vehicle controls
Periredoxin1 (Prx-1) mRNA expression	Szlachcic et al., 2015 [123]	Poland	Induced pluripotent stem cells (iPSCs) from fibroblasts of YAC128 HD transgenic mice	Cellular cultures	Real-time qPCR	Increased expression compared to wild-type
HO-1 mRNA expression	Colin-González et al., 2013 [98]	Mexico	Male Wistar rats weighing 270–310 g. Intrastriatal injection with quinolinic acid	Brain homogenates from the striatum	RT-PCR	Significant increase compared to vehicle controls
HO-1 mRNA and protein expression	Chandra et al..2016 [91]	United States of America	BACHD founder mice expressing expanded human Htt with 97 mixed CAA–CAG	Brain homogenates from the striatum	Real-time PCR and Western blot	Significant decrease compared to wild-type mice

repeats						
HO-1 and HO-2 protein expression	Colin-González et al., 2013 [98]	Mexico	Male Wistar rats weighing 270–310 g. Intrastriatal injection with quinolinic acid	Brain homogenates from the striatum	Western-blot. Chemiluminescence	Significant increase of HO-1, but not of HO-2, compared to vehicle controls
HO enzymatic activity	Colin-González et al., 2013 [98]	Mexico	Male Wistar rats weighing 270–310 g. Intrastriatal injection with quinolinic acid	Brain homogenates from the striatum	Spectrophotometry	Significant increase compared to vehicle controls
NADPH oxidase (NOX) activity	Dominah et al., 2017 [94]	United States of America	*STHdhQ7/Q7 and STHdhQ111/Q111* striatal cell lines. Incubation with chlorpyrifos (CPF)	Cellular cultures	Chemiluminescence	Significant increase compared to wild-type
Pyridoxal kinase (PDXK)	Sorolla et al., 2008 [12]	Spain	20-week-old transgenic Tet/HD94 mice and wild-type littermates	Brain homogenates from the cortex, striatum, and cerebellum	Spectrophotometry	Significant decrease compared to wild-type
Pyridoxal 5´-phosphate (PLP) and pyridoxal kinase (PDXK)	Sorolla et al., 2016 [22]	Spain	3- and 7-week old R6/1 transgenic mice and B6CBAF1/J b mice	Brain homogenates from the cortex, striatum, and cerebellum	Western blot and HPLC coupled to an electrospray ionization quadrupole time-of-flight mass spectrometry	Significant decrease compared to wild-type
Vitamin E	Gopinath et al., 2011 [65]	India	Male Wistar rats weighing 250-300 g. Intraperitoneal administration of 3-NPA	Brain homogenates from the striatum and plasma	HPLC	Significant decrease compared to vehicle controls
Vitamin E	Sumathi et al., 2018 [83]	India	Male albino rats weighing 250-300. Intrastriatal injection with quinolinic acid	Brain homogenates from the striatum	HPLC	Significant decrease compared to vehicle controls
Vitamin E	Purushothaman & Sumathi, 2022 [84]	India	Male albino rats weighing 250-300. Intrastriatal injection with quinolinic acid	Brain homogenates from the striatum	Spectrophotometry	Significant decrease compared to vehicle controls
Vitamin C	Chang et al, 2011 [105]	Singapore	16-week-old male Sprague-Dawley rats weighing 290-350 g. Intraperitoneal administration of 3-NPA	Plasma	Gas chromato-graphy time-of-flight mass spectrome-try -(GC/ TOFMS)	Significant decrease compared to vehicle controls.
Vitamin C	Gopinath et al., 2011 [65]	India	Male Wistar rats weighing 250-300 g. Intraperitoneal	Brain homogenates from the	Enzymatic assay	Significant decrease compared to vehicle controls

			administration of 3-NPA	striatum and plasma		
Vitamin C	Sumathi et al., 2018 [83]	India	Male albino rats weighing 250-300. Intrastriatal injection with quinolinic acid	Brain homogenates from the striatum	Enzymatic assay	Significant decrease compared to vehicle controls
Vitamin C	Purushothaman & Sumathi, 2022 [84]	India	Male albino rats weighing 250-300. Intrastriatal injection with quinolinic acid	Brain homogenates from the striatum	Spectrophotometry	Significant decrease compared to vehicle controls
Vitamin C	Rebec et al., 2002 [128]	United States of America	Transgenic HD (R6/2 strain), mice	Striatum	Voltam-metric recording	Progressive decrease during motor activity compared to wild-type mice
Total thiol	Shivasharan et al., 2012 [67]	India	Adult female Wistar rats weighing 190-200 g. Intraperitoneal administration of 3-NPA	Whole brain homogenates	Spectrophotometry	Significant decrease compared to vehicle controls
Non-protein thiol	Souza et al., 2014 [97]	Brazil	Male Wistar rats weighing 200-250 g. Intraperitoneal administration of 3-NPA	Brain homogenates from the striatum	Spectrophotometry	Significant decrease compared to vehicle controls
Non-protein thiol	Antunes-Wilhelm et al, 2013 [99]	Brazil	Male Wistar rats weighing 200–250 g. Intrastriatal injection with quinolinic acid	Brain homogenates from the striatum	Spectrophotometry	Significant decrease compared to vehicle controls
